# Evaluation of the Cochrane tool for assessing risk of bias in randomized clinical trials: overview of published comments and analysis of user practice in Cochrane and non-Cochrane reviews

**DOI:** 10.1186/s13643-016-0259-8

**Published:** 2016-05-10

**Authors:** Lars Jørgensen, Asger S. Paludan-Müller, David R. T. Laursen, Jelena Savović, Isabelle Boutron, Jonathan A. C. Sterne, Julian P. T. Higgins, Asbjørn Hróbjartsson

**Affiliations:** The Nordic Cochrane Centre, Rigshospitalet 7811, Blegdamsvej 9, 2100 Copenhagen, Denmark; School of Social and Community Medicine, University of Bristol, Canynge Hall, 39 Whatley Road, Bristol, BS8 2PS UK; The National Institute for Health Research Collaboration for Leadership in Applied Health Research and Care West (NIHR CLAHRC West) at University Hospitals Bristol NHS Foundation Trust, Bristol, UK; Methods of Therapeutic Evaluation of Chronic Diseases Team, Epidemiology and Biostatistics, Sorbonne Paris Cité Research Centre, L’Institut National de la Santé et de la Recherche Médicale, Unite Mixte de Recherche 1153, Paris, France; Research Unit for Evidence-Based Medicine, University of Southern Denmark, Odense, Denmark

**Keywords:** Cochrane, Systematic review, Bias, Tool, Comment, User practice, Randomized clinical trial

## Abstract

**Background:**

The Cochrane risk of bias tool for randomized clinical trials was introduced in 2008 and has frequently been commented on and used in systematic reviews. We wanted to evaluate the tool by reviewing published comments on its strengths and challenges and by describing and analysing how the tool is applied to both Cochrane and non-Cochrane systematic reviews.

**Methods:**

A review of published comments (searches in PubMed, The Cochrane Methodology Register and Google Scholar) and an observational study (100 Cochrane and 100 non-Cochrane reviews from 2014).

**Results:**

Our review included 68 comments, 15 of which were categorised as major. The main strengths of the tool were considered to be its aim (to assess trial conduct and not reporting), its developmental basis (wide consultation, empirical and theoretical evidence) and its transparent procedures. The challenges of the tool were mainly considered to be its choice of core bias domains (e.g. not involving funding/conflicts of interest) and issues to do with implementation (i.e. modest inter-rater agreement) and terminology. Our observational study found that the tool was used in all Cochrane reviews (100/100) and was the preferred tool in non-Cochrane reviews (31/100). Both types of reviews frequently implemented the tool in non-recommended ways. Most Cochrane reviews planned to use risk of bias assessments as basis for sensitivity analyses (70 %), but only a minority conducted such analyses (19 %) because, in many cases, few trials were assessed as having “low” risk of bias for all standard domains (6 %). The judgement of at least one risk of bias domain as “unclear” was found in 89 % of included randomized clinical trials (1103/1242).

**Conclusions:**

The Cochrane tool has become the standard approach to assess risk of bias in randomized clinical trials but is frequently implemented in a non-recommended way. Based on published comments and how it is applied in practice in systematic reviews, the tool may be further improved by a revised structure and more focused guidance.

**Electronic supplementary material:**

The online version of this article (doi:10.1186/s13643-016-0259-8) contains supplementary material, which is available to authorized users.

## Background

Since the early 1990s, the number of published systematic reviews of randomized trials, both Cochrane and non-Cochrane reviews, has steadily increased. The ideal of taking a systematic approach to identify, summarise and analyse comparable clinical trials as a basis for therapeutic decisions has become more widespread, and systematic reviews have had a huge impact on clinical research and practice.

However, one obstacle to the usefulness of a systematic review is the possibility that some of the included trials are biased due to flaws in their design, conduct, analysis or reporting. A meta-analysis of biased effect estimates will likely produce a biased pooled analysis with increased precision and greater credibility. Thus, for authors of a systematic review, it is paramount to adequately address the risk of bias in the included trials [1].

For this purpose, the Cochrane tool for assessing risk of bias in randomized clinical trials (i.e. the tool) was released in 2008 and updated in 2011. The tool is based on seven bias domains: sequence generation and allocation concealment (both within the domain of selection bias or allocation bias), blinding of participants and personnel (performance bias), blinding of outcome assessors (detection bias), incomplete outcome data (attrition bias), selective reporting (reporting bias) and an auxiliary domain: “other bias.” For each bias domain, the tool urges users to assign a judgement of “high,” “low” or “unclear” risk of bias and to document the basis for their judgements (e.g. with verbatim quotes). The bias domains of the tool were selected with the intention to cover all fundamental bias mechanisms in randomized trials [2].

Several years have passed since the release of the first version of the tool. Over this period, the tool has been used in numerous systematic reviews, the scientific debate on risk of bias has proceeded (for example, reflecting on the role of source of funding [3–6] or other “meta-biases” [7]) and research publications have analysed user experience [8] and inter-agreement rates [9–11]. Additionally, a complementary tool for assessing non-randomized trials has been developed [12].

Researchers from the original development team and members of the Cochrane Bias Methods Group are planning a revision of the tool. To evaluate the tool and to provide a better basis for the revision, we intended (1) to identify, summarise and analyse published comments on the strengths and challenges of the tool and (2) to describe and analyse how the tool is used in both Cochrane and non-Cochrane reviews.

## Methods

This study involved a review of published comments on the Cochrane tool for assessing risk of bias in randomized clinical trials and an observational study of how the tool is used in systematic reviews (please refer to Additional file 1 for the study’s PRISMA checklist).

### Review of published comments

We sought publications that explicitly commented on the tool. We defined “major comments” as longer comments with a substantial reflection (typically ≥100 words of text) on the strengths or weaknesses of the tool, for example, in the form of an editorial. We also included “minor comments,” which we defined as shorter comments without a substantial reflection (typically <100 words of text) on the strengths or weaknesses of the tool, for example, in the form of minor elements of a discussion in a publication. We excluded “peripheral remarks” on the tool, which we defined as remarks that were implicit or short and tangential. If an author had several publications included with similar comment contents, only the publication with the most detailed comment was considered major.

We searched PubMed, The Cochrane Methodology Register and Google Scholar for publications from the start of 2008 to the end of 2014. No language restriction was applied, and Google Translate was used for non-familiar languages. The search strategy was developed iteratively (see Additional file 2).

One author (LJ) decided on inclusion of publications and categorised them as “major comments” and “minor comments” (and “peripheral remarks”). A second author (AS) checked the categorisation. Two authors (LJ and AS) extracted data independently. Any disagreements were solved by discussion and by consulting a third author (DL or AH).

The following information was extracted: publication year, publication type, tool version considered (i.e. 2008 or 2011) and the exact wording of the comment.

Comments from the included publications were categorised according to whether they expressed “strengths,” “challenges” or “suggestions” and summarised into broader themes (each addressing a similar type of topic). We noted the numerical distribution of comparable comments, but our main intention was a qualitative mapping of the themes addressed and a categorisation according to whether they addressed a core design feature of the tool or an issue related to implementation.

### Observational study of how the tool is used in systematic reviews

One author (DL) identified 100 Cochrane reviews (or Cochrane review updates) from PubMed in reverse chronological order from 31.12.2014 until 20.11.2014 (see Additional file 2). The same author manually identified 100 non-Cochrane reviews from PubMed in reverse chronological order from 31.12.2014 until 22.12.2014. A second author (AS) checked the inclusion. We defined a non-Cochrane review as a self-declared systematic review with at least one included randomized clinical trial. We excluded any non-Cochrane review that was also published as a Cochrane review.

Three authors (AS, DL and LJ) extracted data independently: intervention type (pharmacological or non-pharmacological); inclusion of meta-analyses; number of trials and how many trials were categorised as “high,” “unclear” and “low” risk of bias; the method used for judging risk of bias (or quality) and how it was implemented; the type and frequency of both standard and non-standard domain use; the use of merging or splitting of standard domains (e.g. merging blinding domains or splitting for different outcomes); the use of the “other bias” domain; how risk of bias assessments were incorporated into statistical analysis using sensitivity analyses; whether risk of bias judgements were explicitly mentioned in the abstract, discussion or conclusion; and whether The Grading of Recommendations Assessment, Development and Evaluation (short GRADE) had been incorporated. We compared differences in proportions between Cochrane and non-Cochrane reviews using Fisher’s exact test. In cases where Cochrane or non-Cochrane reviews included both randomized clinical trials and non-randomized clinical trials, we disregarded the non-randomized trials.

## Results

### Review of published comments

**Fig. 1:**
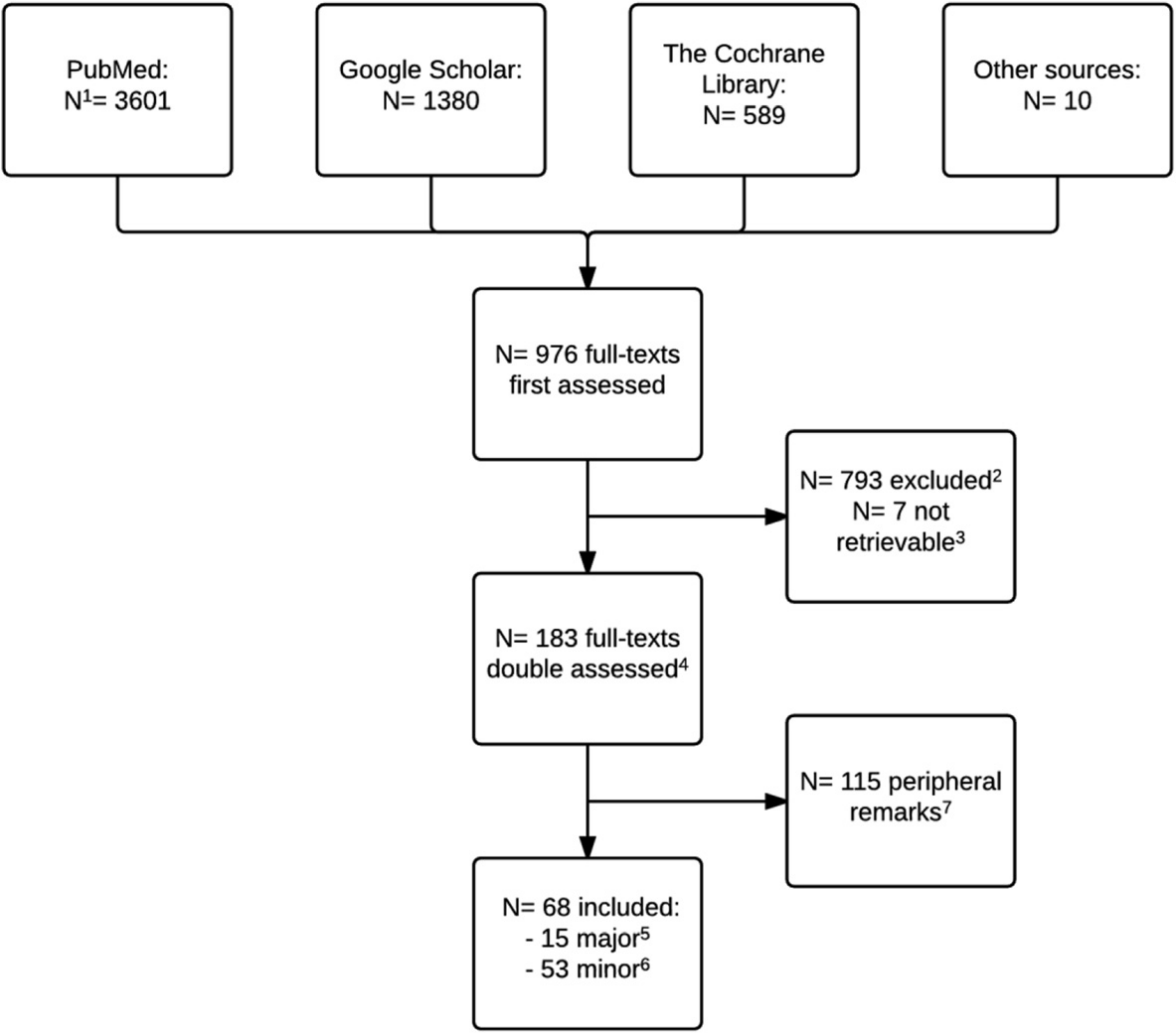
Flowchart of the inclusion of comments on the Cochrane risk of bias tool for randomized clinical trials—evaluation of the Cochrane tool for assessing risk of bias in randomized clinical trials. ^1^N= the number of records/comments screened for inclusion. ^2^Of the 976 full-texts assessed, 793 full-texts did not comment on the Cochrane risk of bias tool for randomized clinical trials (i.e. the tool). ^3^Seven records (ordered through The Royal Danish Library) were not retrievable and therefore not assessed. ^4^183 publications were independently assessed by two authors to check type, categorisation and commentary. ^5^Major comments were defined as longer comments with a substantial reflection (typically ≥100 words of text) on the strengths or challenges of the tool. ^6^Minor comments were defined as shorter comments without a substantial reflection (typically <100 words of text) on the strengths or challenges of the tool. ^7^Peripheral remarks (defined as implicit or short and tangential) were excluded

We read 976 full text publications of which we excluded 908 (Fig. 1). Thus, we included 68 publications, of which we categorised 15 as “major comments” and 53 as “minor comments” (Tables 1 and 2).

**Table 1:** Characteristics of published comments on the Cochrane risk of bias tool for randomized clinical trials—evaluation of the Cochrane tool for assessing risk of bias in randomized clinical trials

Publication characteristics	Number of comments: 68 (100 %)
Publication category	
Major^a^	15 (22 %)
Minor^b^	53 (78 %)
Publication type	
Comment/editorial/letter^c^	6 (9 %)
Survey/qualitative case study	33 (49 %)
Experimental/observational study	23 (33 %)
Other	6 (9 %)
Tool version considered/applied	
2011	54 (79 %)
2008	6 (9 %)
Not specified	8 (12 %)
Year of publication	
2008–2010	9 (13 %)
2011	10 (15 %)
2012	8 (12 %)
2013	14 (20 %)
2014	27 (40 %)

^a^Major comments were defined as longer comments with a substantial reflection (typically ≥100 words of text) on the strengths or challenges of the Cochrane risk of bias tool for randomized clinical trials (i.e. the tool)

^b^Minor comments were defined as shorter comments without a substantial reflection (typically ≤100 words of text) on the strengths or challenges of the tool.

^c^Comments, editorials and letters (to the editor) were defined as such if self-declared

**Table 2:** Selected key points of major comments on the Cochrane risk of bias tool for randomized clinical trials: strengths, challenges and suggestions

First author^a^	Category	Theme	Key point
Armijo-Olivo	Strengths		None mentioned
	Challenges	• Implementation (1.) • Overall risk of bias (2.) • Bias domains (3.)	1. “…the large number of trials classified as high or unclear RoB [risk of bias] casts doubts about the discrimination power of the RoB [risk of bias] tool to […] explain variability of treatments effects across studies…” 2. “…the overall assessment of the RoB [risk of bias] may not be useful to determine quality of individual trials.” 3. “…other methodological factors could be important for evaluating RoB and could be considered for inclusion in the RoB [risk of bias] tool after careful empirical evidence testing.”
	Suggestions	• Guidelines (1.)	1. “Improved guidelines to apply the RoB [risk of bias] tool and revisions to the tool for different health areas are needed.”
Bero	Strengths		None mentioned
	Challenges	• Bias domains (1.)	1. “The current Cochrane risk of bias tool is insufficient to assess bias related to study funding sources.”
	Suggestions	• Funding (1.)	1. “…the Cochrane risk of bias tool should include funding source as a standard item because: 1. Funding source fits the definition of bias, 2. There is empirically-based evidence of bias related to funding source, 3. The observed bias related to funding source cannot be captured by the risk of bias criteria currently assessed with the risk of bias tool, 4. Risks of bias are not mutually exclusive, 5. Bias may be related to funding source even when all studies are industry-funded.”
Boutron	Strengths	• Aims (1. 2.) • Improvement (3.) • Transparency (1.)	1. “…the tool aims at being completely transparent, with a separation of the facts and reviewers’ judgments. This aim is particularly important because reviewers, editors, and readers can challenge the author on the judgment.” 2. “…the tool is intended to assess the risk of bias related to the design, conduct, and analyses of the trial and not the quality of reporting.” 3. “This tool has been an important step forward in the assessment of the risk of bias in systematic reviews and meta-analyses.”
	Challenges		None mentioned
	Suggestions		None mentioned
De Bruin	Strengths		None mentioned
	Challenges	• Implementation (1.)	1. “…many do assess methodological quality, but very few incorporate them [/risk of bias assessments] in their analyses.”
	Suggestions	• Guidelines (1.)	1. “…systematic reviewers could consider adapting the risk-of-bias tool to the literature…”
Hartling	Strengths		None mentioned
	Challenges	• Implementation (1.) • Overall risk of bias (2.) • Special situations (3.)	1. “Low agreement between reviewers suggests the need for more specific guidance regarding interpretation and application of the Risk of Bias (ROB) tool…” 2. “The majority of trials in the sample were assessed as high or unclear risk of bias…This raises concerns about the ability of the ROB [risk of bias] tool to detect differences across trials that may relate to biases in estimates of treatment effects.” 3. “…trials with different design features (e.g., crossover) or hypotheses (e.g., equivalence, non-inferiority), and those examining non-pharmacological interventions appear to create more ambiguity for risk of bias assessments.”
	Suggestions	• Guidelines (1.)	1. “There is a need for more detailed guidelines to apply […] the ROB [risk of bias] tool and […] further testing with the modified tool is warranted.”
Hróbjartsson	Strengths	• Aims (1.) • Background (1.)	1. “The risk of bias tool provides a standardised approach, based on items selected on both theoretical and empirical grounds, and following broad consultations with clinical research methodologists.”
	Challenges	• Bias domains (2.) • Implementation (1.)	1. “The risk of bias tool is a comparatively recent development that still likely needs refinement.” 2. “It is not clear that the risk of bias tool in its present version addresses this problem [of funding] adequately.”
	Suggestions		None mentioned
Ivers	Strengths		None mentioned
	Challenges	• Bias domains (1.) • Implementation (2.) • Overall risk of bias (3.)	1. “The risk of bias tool does not capture all sources of methodological bias and poor reporting interferes with the assessment of many domains.” 2. “While the overall risk of bias assessment using the Cochrane Risk of Bias Tool has been shown to differentiate effect sizes (i.e. higher risk of bias studies usually have larger effect sizes), 10 studies at high risk of bias may still offer valuable knowledge…” 3. “…assigning trials with high risk of bias in a single domain a status of high risk of bias overall may be arguable.”
	Suggestions		None mentioned
Jefferson	Strengths	• Aims (1.)	1. “The real strength of the risk of bias tool appears not to be in the final judgements it enables, but rather in the process it helps facilitate: critical assessment of a clinical trial.”
	Challenges	• Bias domains (1. 3.) • Implementation (2.)	1. “The current Cochrane risk of bias tool is not adequate for the task as it does not reliably identify all types of important biases, and nor does it organise and check the coherence of large amounts of information.” 2. “We found the Cochrane risk of bias tool to be difficult to apply to clinical study reports…[since]…its use lends itself to a checklist approach (in which each design item is sought and, if found, eliminated from the bias equation rather than with thought and consideration).” 3. “Many of the variables we found to be important when assessing the trial (e.g. date of trial protocol, date of un-blinding, date of participant enrolment) are simply not captured in the risk of bias tool…”
	Suggestions		None mentioned
Katikireddi	Strengths		None mentioned
	Challenges	• Implementation (1.)	1. “…reviewers are struggling to understand and/or operationalize current guidance on how to conduct and incorporate critical appraisal [/risk of bias] within synthesis.”
	Suggestions	• Guidelines (1.) • Research (1.)	1. “Further research is required to establish the relative importance of different forms of bias and their likely impact […] and also to clarify how critical appraisals should be incorporated into SR [systematic review] findings.”
Morissette	Strengths	• Aims (1.)	1. “The Cochrane 'Risk of bias' tool differs from other quality appraisal tools because it questions the degree to which a study’s results should be believed…”
	Challenges	• Implementation (1.)	1. “The results of our review provide no clear guidance as to whether risk of bias assessments should be completed in a blind or un-blind manner.”
	Suggestions	• Research (1.)	1. “…we encourage further research in this area [of blind vs. un-blind risk of bias assessment] and recommend using all of the important components of the Cochrane 'Risk of bias' tool.”
Moustgaard	Strengths		None mentioned
	Challenges	• Implementation (1.)	1. “No characterization of subjective vs. objective outcomes relevant to risk of performance bias is given explicitly in the Cochrane Handbook nor did we find it in the methodological articles or the clinical trial reports we reviewed.”
	Suggestions		None mentioned
Roseman	Strengths		None mentioned
	Challenges	• Bias domains (1.)	1. “…inclusion of risk of bias from conflicts of interest could reflect mechanisms through which industry involvement can influence study outcomes that are not fully captured by the current domains of the risk of bias tool.”
	Suggestions	• Funding (1.)	1. “…we recommend that the Cochrane Collaboration reconsider its position that trial funding and trial author-industry financial ties not be included in the risk of bias assessment.”
Savović	Strengths	• Aims (3.) • Background (1.) • Transparency (2.)	1. “…[the tool has] a standardized approach to bias assessments…” 2. “…[the tool has] transparency provided by requesting quotes…” 3. “…[the tool provides] a platform to encourage critical thinking.”
	Challenges	• Bias domains (1.) • Implementation (2.)	1. “Some of the items that authors have included (such as sample size calculations and funding source) are explicitly discouraged in the Cochrane Handbook guidance. While there is evidence that some factors are empirically associated with effect estimates, such as single versus multicentre design, early stopping of trials and funding source [14-16], the extent to which these should be considered alongside the main bias domains is still a topic of debate.” 2. “The main purpose of this evaluation was to identify potential problems with the RoB [risk of bias] that can be rectified, and we suspect that users who encountered problems are more likely to have responded. This speculation is based on the high proportion of respondents who reported having problems with some aspects of the RoB tool, especially with individual RoB domains.”
	Suggestions	• Guidelines (1.)	1. “It is important that guidance and training materials continue to be developed for all aspects of the tool…”
Sterne	Strengths		None mentioned
	Challenges	• Bias domains (1.)	1. “The current RoB [risk of bias] tool does not work well for assessment of selective reporting.”
	Suggestions	• Funding (1.)	1. “…the Cochrane risk of bias tool should not include funding source as a standard item.”
Vale	Strengths		None mentioned
	Challenges	• Implementation (1. 2.) • Bias domains (2.)	1. “The Cochrane Handbook states that because the ability to measure the true bias (or even the true risk of bias) is limited, then the possibility to validate a tool to assess that risk is also limited. Nevertheless, authors of Cochrane systematic reviews are required to use the Cochrane risk of bias tool.” 2. “Assessing risk of bias was particularly difficult for the more subjective domains [i.e. ‘selective outcome reporting’ and ‘other bias’].”
	Suggestions		None mentioned

Major comments were defined as longer comments with a substantial reflection (typically ≥100 words of text) on the strengths or challenges of the Cochrane risk of bias tool for randomized clinical trials (i.e. the tool)

^a^See Additional file 2 for references

The strengths of the tool were addressed in five “major comments” relating to three themes: aims, developmental basis and transparency. The comments praised the tool for aiming to assess conduct (and not reporting), being based on theoretical and empirical evidence and on broad consultation and facilitating transparent assessment of bias.

The challenges of the tool were addressed in 15 “major comments” relating to four themes: choice of the core bias domains, implementation, overall risk of bias and special situations. The comments on choice of core bias domains expressed concern whether the chosen domains comprehensively address all threats to validity (for example, five comments reflected on including funding as an independent bias domain). Comments on implementation pointed to difficulties in the subjective interpretation of the tool and expressed concerns about modest inter-observer agreement, difficulty in assessing selective reporting of outcomes, terminological ambiguity (i.e. of the terms subjective/objective) and the low proportion of reviews using risk of bias assessments as a basis for sensitivity analyses. The comments on overall risk of bias expressed concern about the challenges in assigning an overall risk of bias to a trial based on risk of bias of single domains to the trial as such. A single comment regarded the special situation where the tool was used to assess risk of bias based on clinical study reports (and not clinical trial publications).

Specific suggestions to improve the tool were included in nine “major comments” relating to three themes: improved guidelines, further research and the inclusion of funding as a bias domain. The comments on guidelines suggested that updated and improved guidance and more training options for users were needed. The comments on research suggested further methodological research (for example, blind versus non-blind risk of bias assessments). The comments on funding suggested that funding/conflicts of interest should be incorporated into the tool as a specific bias domain.

All themes addressed in the “major comments” were represented in the “minor comments” (see Additional file 2). Additional themes addressed only in the “minor comments” included graphical representation, external validity and non-randomized designs. Specifically, (i) one comment praised the tool for its graphical representation of risk of bias assessments, (ii) one comment criticised that the tool does not address external validity (and only focuses on internal validity) and (iii) one comment noted that non-randomized trials should be included in Cochrane reviews and should be addressed in risk of bias assessments. The latter two suggestions are inconsistent with the aim of the tool, which is to assess only bias (i.e. internal validity) in randomized clinical trials. Such comments help to unveil the assumptions and basic structure of the tool but would be difficult to implement without significantly changing the tool.

Other comments reflected concerns about the implementation of the tool. An example is the suggestion for improved guidelines for how to assess selective outcome reporting. Also, improved training options and more detailed guidelines aimed to improve agreement rates address the implementation of the tool. Such suggestions are easier to implement while keeping the fundamental structure of the tool intact.

### Analysis of user patterns in systematic reviews

All Cochrane reviews assessed risk of bias using the Cochrane risk of bias tool (100/100, 100 %) (Tables 3 and 4). Most of the non-Cochrane reviews assessed risk of bias (80/100, 80 %), with the Cochrane tool being the most frequently used (31/80, 39 %). Other tools and scales used to assess risk of bias included the Jadad Quality Assessment Scale (19/80, 24 %) [13] and the Physiotherapy Evidence Database (short PEDro) scale (5/80, 6 %) [14] (Table 4).

**Table 3:** Characteristics of included Cochrane and non-Cochrane reviews—evaluation of the Cochrane tool for assessing risk of bias in randomized clinical trials

Publication characteristics	100 Cochrane reviews (100 %)	100 non-Cochrane reviews (100 %)	*P* value*
Intervention			
Pharmacologic	55 (55 %)	29 (29 %)	0.020
Non-pharmacologic	45 (45 %)	71 (71 %)	0.061
Review has ≥1 meta-analysis			
Yes	85 (85 %)	45 (45 %)	0.0065
Included trials			
Number of randomized clinical trials in total	1242	1249	
-Low^a^ risk of bias	74 (6 %)	25 of 424^e^ (6 %)	1.00
-Unclear^b^ risk of bias	407 (33 %)	226 of 424^e^ (53 %)	0.0001
-High^c^ risk of bias	761 (61 %)	173 of 424^e^ (41 %)	0.0001
Reviews with ≥1 low risk of bias trial and ≥1 high risk of bias trial	26 (26 %)	6 of 18^f^ (33 %)	0.60
Reviews with ≥1 low risk of bias trial and ≥1 high or unclear risk of bias trial	32 (32 %)	8 of 18^f^ (44 %)	0.47
Number of randomized clinical trials included^d^ in a review			
-One to five	39 (39 %)	38 (38 %)	1.00
-Six to ten	23 (23 %)	26 (26 %)	0.75
->Ten	38 (38 %)	36 (36 %)	0.89

*P values were calculated with Fisher’s two-tailed exact test

^a^If a trial had all standard domains (not including the “other bias” domain) judged as “low” risk of bias, we defined the trial as “low risk of bias”

^b^If a trial had at least one of the standard domains (not including the “other bias” domain) judged as “unclear” risk of bias and no domains judged as “high” risk of bias, we defined the trial as “unclear risk of bias.” The judgement of at least one standard risk of bias domain (not including the “other bias” domain) as “unclear” was found in 1103 of 1242 included randomized clinical trials (89 %)

^c^If a trial had at least one of the six standard domains (not including the “other bias” domain) judged as “high” risk of bias, we defined the trial as “high risk of bias”

^d^We only included systematic reviews with one or more randomized clinical trials included in their analyses

^e^It was only possible to assess whether a trial was judged as “low,” “unclear” or “high” risk of bias in 18 non-Cochrane reviews (which provided information on risk of bias judgements for all six standard domains (not including the “other bias” domain) for individual trials via a “risk of bias graph/summary” or “characteristics of studies” section)

^f^The 18 non-Cochrane reviews included 424 randomized clinical trials in total

**Table 4:** User patterns of risk of bias implementations in Cochrane and non-Cochrane reviews—evaluation of the Cochrane tool for assessing risk of bias in randomized clinical trials

Risk of bias implementation	100 Cochrane reviews (100 %)	100 non-Cochrane reviews (100 %)	*P* value*
Risk of bias assessment in reviews			
Any risk of bias (or quality) assessment	100 (100 %)	80 (80 %)	0.30
Cochrane risk of bias tool	100 (100 %)	31 of 80 (39 %)	0.0002
Jadad scale	0 (0 %)	19 of 80 (24 %)	0.0001
PEDro scale	0 (0 %)	5 of 80 (6 %)	0.019
Own construct or other scale	0 (0 %)	25 of 80 (31 %)^c^	0.0001
Descriptive use of risk of bias assessment			
Explicit mentions risk of bias in abstract	80 (80 %)	18 of 31^d^ (58 %)	0.42
Explicit mentions risk of bias in discussion/conclusion	89 (89 %)	25 of 31^d^ (81 %)	0.76
Explicit mentions risk of bias in both abstract and discussion/conclusion	73 (73 %)	15 of 31^d^ (48 %)	0.31
Sensitivity and subgroup analyses based on risk of bias			
Review planned (in *methods*) to do sensitivity analyses	70 (70 %)	8 of 80 (10 %)	0.0001
Review performed sensitivity analyses	19 (19 %)	11 of 80 (14 %)	0.55
Based on overall risk of bias	2 of 19 (11 %)	9 of 11 (82 %)	0.015
Based on individual risk of bias domains	9 of 19 (47 %)	2 of 11 (18 %)	0.45
Unclear what analyses were based on	8 of 19 (42 %)	0 of 11 (0 %)	0.077
Review performed, but did not plan sensitivity analyses	1 of 19 (5 %)	8 of 11 (72 %)	0.0084
Review performed subgroup analyses^a^	2 (2 %)	0 of 80 (0 %)	0.50
Review planned, but did not perform analyses	50 of 70 (71 %)	5 of 8	0.52
Due to insufficient data^b^	41 of 50 (82 %)	3 of 5	0.73
No explanation provided	9 of 50 (18 %)	2 of 5	0.33
GRADE			
Review incorporated GRADE	64 (64 %)	4 of 80 (5 %)	0.0001

*P values were calculated with Fisher’s two-tailed exact test

^a^All subgroup analyses were based on “low” versus “high” risk of bias

^b^“Insufficient data” was due to few trials included in the review or few trials judged as “low risk of bias”

^c^15 non-Cochrane reviews made their own risk of bias construct/tool, eight incorporated two constructs/tools and the following constructs/tools (/methods) were used 18 times in total: CASP (×2), CEBM, Chalmers, CONSORT (×2), CTAM, Downs and Black criteria (×2), Evidence-based medicine toolkit, GRADE (×2), Methods Guide for Effectiveness and Comparative Effectiveness Reviews, MOOSE (×2), Newcastle Ottawa, QUOROM and STROBE

^d^31 of 100 non-Cochrane reviews used the Cochrane risk of bias tool for randomized clinical trials (i.e. the tool) and were compared to the 100 Cochrane reviews that used the tool for randomized clinical trials

The majority of Cochrane reviews included one or more meta-analyses (85/100, 85 %). According to the information reported in their methods section, most of the Cochrane reviews had planned to perform sensitivity analyses based on risk of bias (70/100, 70 %). One fifth of the Cochrane reviews reported to have performed sensitivity analyses (19/100, 19 %). Few reviews based sensitivity analyses on an overall risk of bias (2/19, 11 %). Most reviews based sensitivity analyses on individual bias domains (9/19, 47 %) or did not state what sensitivity analyses were based on (8/19, 42 %). The majority of the Cochrane reviews who did not conduct the planned analyses reported that the lack thereof was due to insufficient data (41/50, 82 %), either because there were few trials included in the review or few trials with “low” risk of bias. The remaining reviews did not explain why they did not perform the planned analyses (9/50, 18 %) (Tables 3 and 4).

One tenth of the non-Cochrane reviews that had any risk of bias assessment reported plans for sensitivity analyses based on risk of bias assessments (8/80, 10 %). One in seven of all the non-Cochrane reviews reported to have performed sensitivity analyses based on risk of bias or quality assessments (11/80, 14 %). In nine reviews, the sensitivity analyses were based on an overall risk of bias (9/11, 82 %) (Table 4).

Two Cochrane reviews performed subgroup analyses (both with “low” versus “high” risk of bias) (2/100, 2 %). None of the non-Cochrane reviews performed subgroup analyses based on risk of bias.

Most Cochrane reviews explicitly commented on risk of bias assessments in the discussion and/or conclusion (89/100, 89 %), although fewer incorporated this information into the abstract (80/100, 80 %). Most of the non-Cochrane reviews that applied the Cochrane tool and some of the non-Cochrane reviews that applied non-Cochrane tools explicitly commented on risk of bias assessments in the discussion and/or conclusion (Cochrane tool: 25/31, 81 %; non-Cochrane tools: 12/49, 24 %) and more than half incorporated this information into the abstract (Cochrane tool: 18/31, 58 %; non-Cochrane tools: 30/49, 61 %). No significant differences were found between the non-Cochrane reviews that used the Cochrane tool versus the non-Cochrane reviews that used other risk of bias tools when comparing the use of risk of bias results in the abstract and discussion/conclusion.

The majority of Cochrane reviews (64/100, 64 %) and few non-Cochrane reviews (4/80, 5 %) incorporated GRADE in their overall assessment of confidence in the results (Table 4).

The majority of Cochrane reviews applied all standard domains (59/100, 59 %). Only few Cochrane reviews explicitly assessed risk of bias on an outcome level (i.e. differentiating between subjective versus objective outcomes) (12/100, 12 %). Most Cochrane reviews (88/100, 88 %) performed one risk of bias assessment without making it clear whether this assessment concerned a single outcome, a group of outcomes or the trial as a whole. A similar pattern was seen for non-Cochrane reviews (Table 5).

**Table 5:** Use of risk of bias and risk of bias domains in the Cochrane and non-Cochrane reviews that applied the Cochrane risk of bias tool for randomized clinical trials

Use of risk of bias and risk of bias domains	100 Cochrane reviews (100 %)	31 non-Cochrane reviews (100 %)^f^	*P* value*
Use of risk of bias			
Summarises risk of bias on an outcome level^a^	12 (12 %)	2 (6 %)	0.73
Unclear what level risk of bias was summarised on^b^	88 (88 %)	29 (94 %)	0.88
Use of risk of bias standard^c^ domains			
Review uses the 2011 tool version	100 (100 %)	26 (84 %)	0.65
Review uses all standard^c^ domains	59 (59 %)	16 (52 %)	0.73
-Sequence generation	100 (100 %)	30 (97 %)	1.00
-Allocation concealment	100 (100 %)	30 (97 %)	1.00
-Blinding of patients and care providers	62 (62 %)	21 (68 %)	0.87
-Blinding of outcome assessors	65 (65 %)	20 (65 %)	1.00
-Incomplete outcome data	99 (99 %)	29 (94 %)	0.88
-Selective reporting	87 (87 %)	25 (81 %)	0.88
Merging and splitting of standard^c^ domains			
Review merges two standard^c^ domains	37 (37 %)	8 (26 %)	0.53
-Merges risk of bias domains on an outcome level^d^	6 of 37 (16 %)	0 of 8 (0 %)	0.57
-Does not merge risk of bias domains on an outcome level	31 of 37 (84 %)	8 of 8 (100 %)	0.79
Review splits a standard^c^ domain into two or more domains^e^	18 (18 %)	7 (23 %)	0.62

*P values were calculated with Fisher’s two-tailed exact test

^a^One or more domains were separately assessed for more than one outcome or groups of outcomes (i.e. subjective versus objective outcomes)

^b^Review has a singular risk of bias assessment despite more than one outcome included in the review. No review based its risk of bias assessment on a singular or primary outcome

^c^The six standard domains (not including the “other bias” domain) included in the Cochrane risk of bias tool for randomized clinical trials (i.e. the tool)

^d^i.e. merges blinding of patients and care providers with blinding of outcome assessors into one blinding domain and evaluates blinding for subjective/objective or explicit (≥2) outcomes.

^e^i.e. splits blinding of patients and care providers into blinding of personnel and blinding of patients or splits incomplete outcome data into assessment of intention-to-treat and assessment of dropouts.

^f^31 of 100 non-Cochrane reviews used the Cochrane risk of bias tool for randomized clinical trials (i.e. the tool) and were compared to the 100 Cochrane reviews that used the tool for randomized clinical trials

One third of the Cochrane reviews merged standard bias domains (37/100, 37 %), most often merging “performance bias” and “detection bias” into a single blinding bias domain (31/37, 84 %) (predominantly done in updates of reviews that had originally used the 2008 version of the tool in which the domains were merged (21/31, 68 %)). Approximately one fifth of the Cochrane reviews split a standard bias domain into separate sub-entities (18/100, 18 %), for example, blinding (within the performance bias domain) was split into blinding of personnel and blinding of patients or incomplete outcome data (i.e. attrition bias) was split into assessment of intention-to-treat and assessment of dropouts. Again, a similar pattern was seen for non-Cochrane reviews (Table 5).

A minority of Cochrane reviews added non-standard bias domains to the tool (11/100, 11 %). “Baseline imbalance” (6/11, 55 %) and “funding/conflicts of interest” (5/11, 45 %) were the most used. A similar pattern was found for non-Cochrane reviews (Table 6). The majority of Cochrane reviews used the “other bias” domain option for the same purpose (73/100, 73 %). “Baseline imbalance” (33/73, 45 %) and “funding/conflicts of interest” (23/73, 32 %) were also the most used “other biases.” Most non-Cochrane reviews that used the Cochrane tool included the “other bias” domain (17/31, 55 %), but none of the non-Cochrane reviews reported what specific items were considered as “other biases” (Table 6).

**Table 6:** Use of additional non-standard domains and the “other bias” domain in the Cochrane and non-Cochrane reviews that applied the Cochrane risk of bias tool for randomized clinical trials

Use of additional domains and “other bias”	100 Cochrane reviews (100 %)	31 non-Cochrane reviews (100 %)^c^	*P* value*
Additional domains			
Any additional domain(s)	11 (11 %)	6 (19 %)	0.37
-Adds “baseline imbalance”	6 of 11 (55 %)	2 of 6 (33 %)	1.00
-Adds “funding” or “conflicts of interest”	5 of 11 (45 %)	1 of 6 (17 %)	0.62
-Adds “intention to treat”	2 of 11 (18 %)	2 of 6 (33 %)	0.62
-Adds “compliance”	2 of 11 (18 %)	1 of 6 (17 %)	1.00
-Adds “follow up”	3 of 11 (27 %)	2 of 6 (33 %)	1.00
-Adds “timing of outcome assessment”	2 of 11 (18 %)	1 of 6 (17 %)	1.00
-Adds “overall risk of bias”	1 of 11 (9 %)	4 of 6 (67 %)	0.14
-Adds other additional domain^a^	6 of 11 (55 %)	2 of 6 (33 %)	1.00
Other bias^b^			
Includes the “other bias” domain	73 (73 %)	17 (55 %)	0.41
-Used for “baseline imbalance”	33 of 73 (45 %)	0 of 17 (0 %)	0.0059
-Used for “funding” or “conflicts of interest”	23 of 73 (32 %)	2 of 17 (12 %)	0.24
-Used for “intervention differed between groups”	16 of 73 (22 %)	0 of 17 (0 %)	0.069
-Used for “unclear reporting by trial publication author”	15 of 73 (21 %)	0 of 17 (0 %)	0.12
-Used for “trial design”	11 of 73 (15 %)	0 of 17 (0 %)	0.20

*P values were calculated with Fisher’s two-tailed exact test

^a^All of the following other additional domains appeared ones in review samples: Cochrane reviews: “co-interventions avoided or similar,” “confounding variables,” “definition of incomplete response,” “definition of local recurrence,” “method of follow up” and “size”; non-Cochrane reviews: “co-intervention” and “double blinding”

^b^“Other bias”—comments were interpreted and categorised (e.g. the “other bias” comment “There *were baseline differences between groups*.” was categorised as “baseline imbalance”). The five most used “other bias”—categories are listed

^c^31 of 100 non-Cochrane reviews used the Cochrane risk of bias tool for randomized clinical trials (i.e. the tool) and were compared to the 100 Cochrane reviews that used the tool for randomized clinical trials

Very few of the randomized clinical trials included in the Cochrane reviews had all standard domains judged as “low” risk of bias (74 of 1242 trials, 6 %). Most had at least one standard domain judged as “unclear” risk of bias (407 of 1242 trials, 33 %) or as “high” risk of bias (761 of 1242 trials, 61 %). A similar pattern was found for the non-Cochrane reviews (Table 3).

Thus, only a few reviews could conduct sensitivity analyses based on overall risk of bias, e.g. the Cochrane reviews with at least one trial with all standard domains judged as “low” risk of bias and at least one trial with one bias domain judged as “high” risk of bias (26/100, 26 %) (or as “high”/“unclear” risk of bias (32/100, 32 %)). A similar pattern was found for the non-Cochrane reviews (Table 3).

## Discussion

Published comments about the Cochrane risk of bias tool considered it to be an important step forward but highlighted some challenges including its omission of funding/conflicts of interest and its modest inter-agreement rates. Suggestions for improvement included more explicit guidelines and training options. The tool was used in 100 % of Cochrane reviews and in 31 % of non-Cochrane reviews in a sample published towards the end of 2014. Often the tool was implemented in a non-recommended way. Also, 70 % of Cochrane reviews planned to use the risk of bias assessment as basis for sensitivity analyses, but only 19 % of Cochrane reviews conducted such analyses, in many cases, because there were few trials with “low” risk of bias.

### Strengths and weaknesses

We are not aware of other reviews of published comments on the Cochrane risk of bias tool. Our study complements previous studies of user experience [8] and inter-observer variance [9–11].

It is challenging to search for published comments as not all are indexed in standard databases. However, we focused on “major comments,” which are more reliably identified. It is reasonable to assume that the threshold for publishing a comment pointing out a problem with the tool (and maybe suggesting an improvement) is lower than for publishing a comment praising the tool. Thus, we consider the qualitative summary of the expressed themes as more interesting than the quantitative distribution of the themes. The analyses of how the tool was used were based on samples of representative and contemporary Cochrane and non-Cochrane reviews, enabling both a description and comparison between the two types of reviews.

### Other similar studies

Based on feedback from focus groups and an online survey, Savović and colleagues concluded that users of the Cochrane tool identified positive experiences and perceptions of the tool and that revisions and associated guidance as well as improved provision of training may improve implementation [8]. Several studies have analysed the assessment of risk of bias in systematic reviews [10–15]. Hartling and colleagues and Armijo-Olivo and colleagues concluded unsatisfactory agreement rates by users of the tool and suggested the need for more detailed guidance in assessing the risk of bias [9, 15]. Comments made by the authors of all three studies are included in our study.

Hopewell and colleagues [16] studied assessment of risk of bias in Cochrane and non-Cochrane reviews indexed in The Database of Abstracts of Reviews of Effects (DARE) [17] and published in 2012. They reported that all reviews incorporated some kind of assessment of risk of bias, even though Cochrane reviews more often specified which tool was used. Also, the Cochrane tool was used more often in Cochrane reviews (and the Jadad scale was used less often). A low proportion of reviews incorporated sensitivity analyses based on risk of bias in their conclusion.

Our study confirms and expands on the findings of Hopewell and colleagues. We found that all 100 Cochrane reviews in our sample used the Cochrane risk of bias tool, but that only one in five Cochrane reviews conducted sensitivity analyses based on risk of bias assessments, despite the fact that seven in ten had planned to do so.

### Mechanisms and implications

Based on the degree of implementation, the tool has proven successful. All Cochrane reviews and a fair proportion of non-Cochrane reviews used the tool in 2014. However, the tool is often used in ways not recommended.

Firstly, both Cochrane and non-Cochrane reviews implemented non-standard domains, either as fully new domains or incorporated into the “other bias” function. Approximately one in six Cochrane reviews added “intervention differed between groups” under “other bias,” though this problem is intended to be addressed under “performance bias.” Furthermore, a similar proportion of Cochrane reviews added “unclear reporting” under “other bias,” although the tool specifically addresses conduct and not reporting (unclear reporting would normally result in contacting trial authors for clarification). Thus, there seems to be a widespread uncertainty as to the scope of what the tool seeks to evaluate. Adding bias domains and using the “other bias” option are primarily intended for special situations, for example, when assessing crossover trials. Thus, better guidance as to what is meant by “bias,” “bias domain” and the basic purpose of the tool is warranted.

Secondly, only a minority of reviews used the risk of bias assessments as a basis for sensitivity analyses. This problem seems to be a result of few trials having a “low” risk of bias, although sensitivity analyses may be based on “unclear” versus “high” risk of bias. Only 6 % of the trials included in our review sample had been classified as “low” risk of bias for all domains. It is unclear whether such a low proportion (also found by e.g. Hartling and colleagues [9] and Hopewell and colleagues [16]) is a fair reflection of the “true” risk of bias in trials or whether the tool as currently applied is too sensitive (or authors simply do not use all sources of information as recommended and possibly opt for “unclear” based on the published report). A better guideline on how to move from the level of individual bias domains to an overall risk of bias is warranted.

Thirdly, most reviews based their risk of bias assessment on a singular risk of bias assessment despite including more than one outcome and several reviews (mostly updates) merged “blinding of participants and personnel” and “blinding of outcome assessor” into a single blinding bias domain. The latter was recommended in the 2008 version of the tool, but not in the updated 2011 version [18]. Hopefully, the merging of blinding associated bias domains will be addressed when the reviews in question are updated (again).

Fourthly, risk of bias is very often assessed based on incomplete or missing information. The judgement of at least one risk of bias domain as “unclear” was found in 1103 of 1242 included randomized clinical trials (89 %). Though “unclear” may be a reasonable option in some trials, this large proportion is a considerable problem. In many cases, the uncertainty can be resolved by contacting trial authors (who are often able to provide the information) or by searching publicly available trial registers. Occasionally, one may access trial protocols, internal company study reports or reports by drug regulation agencies (such as the United States’ Food & Drug Administration) to facilitate better risk of bias judgements [19]. Improved guidelines on how to access and acquire the relevant information for assessing risk of bias are warranted.

Furthermore, low inter-rater agreement rates for risk of bias assessors are a potential problem for users of systematic reviews. Readers may consider whether a review’s conclusion would have been different if other reviewers had assessed the risk of bias in the included trials. It is prudent to check the risk of bias assessments in a review. Fortunately, the tool has a configuration that facilitates such checking. Studies assessing between-rater agreement for complex assessment procedures often have modest agreement rates [20], which in some cases may be improved with training [21]. The Cochrane tool is no exception. Disagreement seems to occur when terminology is used inconsistently (e.g. for blinding [22]), when judgements are based on insufficient information or when the intervention is more complex (e.g. in non-pharmacological trials [9]). In addition, reviewers often encounter problems when assessing the domains “incomplete outcome data” and “selective outcome reporting” [8]. Clarified terminology, revised structure, better training options and guidance will hopefully improve agreement rates. It will be interesting to read the result of a forthcoming study on the impact of training [23].

Funding/conflicts of interest is also a challenge for the tool. It is widely believed that industry funding and other conflicts of interest are associated with higher estimates of treatment effects in randomized trials [24]. It is more controversial whether this association is appropriately accounted for by adding “funding/conflicts of interest” as an independent bias domain. Adding a domain would go against the logic structure of the tool, which is based on core bias domains that reflect fundamental, independent bias mechanisms. An alternative option would be to address the issue within the existing bias domains (for example, under risk of selective outcome reporting), while paying careful attention to any clinical or methodological differences between industry funded and non-funded trials, such as selection of control groups. The problem with the latter option is that detailed information on trial conduct is often missing. It is notable that 5 % of Cochrane reviews added funding as a separate domain and that 32 % incorporated funding into the “other bias” function. Clearly, more work is needed on this issue.

A general tension exists between bias in randomized trials as defined mechanistically in the tool, and as defined empirically based on results from meta-epidemiological studies. Several design features of randomized clinical trials have been reported in meta-epidemiological studies to be associated with exaggerated treatment effects, such as sample size [25], development country status [26], single centre status [27] and stopping a trial early [28]. The list of potential bias domains selected purely on empirical grounds will quickly become quite large and involve a risk of spurious inclusion of bias domains that are secondary in nature (and thus, in principle, explainable by the core bias domains). However, an open question is whether a pragmatic and careful selection of a few empirically defined bias domains that are simple to assess (such as sample size or single centre status) may act as proxy measures and supplement a risk of bias tool based on mechanistically defined core bias domains.

## Conclusions

Based on published comments, the Cochrane tool for assessing risk of bias in randomized clinical trials is regarded as an important step forward but challenged by how to deal with the risk of bias associated with funding/conflicts of interest and modest inter-rater agreement. The tool is used in a very high proportion of Cochrane reviews and in many non-Cochrane reviews, but often in a non-recommended way, for example, by incorporating additional bias domains. The tool has become the standard approach to assess risk of bias in randomized clinical trials. Its implementation may be further improved by a revised structure, further research and more focused guidance.

## Additional file

Additional file 1: PRISMA 2009 Checklist. (DOCX 179 kb) Additional file 2: Appendices - Evaluation of the Cochrane tool for assessing risk of bias in randomized clinical trials. (DOCX 229 kb)

## Abbreviations

DARE: The Database of Abstracts of Reviews of Effects
GRADE: The Grading of Recommendations Assessment, Development and Evaluation
PEDro: Physiotherapy Evidence Database
