# Supplementary material for: Evaluation of the Cochrane tool for assessing risk of bias in randomized clinical trials: overview of published comments and analysis of user practice in Cochrane and non-Cochrane reviews
Source: Syst Rev. 2016 May 10;5:80. doi: 10.1186/s13643-016-0259-8 (PMC4862216; doi:10.1186/s13643-016-0259-8)
Supplement: Additional file 2: — Appendices - Evaluation of the Cochrane tool for assessing risk of bias in randomized clinical trials. (DOCX 229 kb) [file 13643_2016_259_MOESM2_ESM.docx]

**Appendices**

**Appendix 1: Review of published comments: Search strategy for identifying the comments on the Cochrane risk of bias tool for randomized clinical trials**

The search strategy was developed iteratively with keywords (and synonyms): risk (chance, likely*, probab*), bias (qualit*, valid*, (systematic) error, risk of bias, RoB), tool (instrument, assess*, examin*, score, apprais*), Cochrane (CDSR, CCRBT, ROBT, systematic, review, (meta-)analys*), editor* (comment*, letter* (to the editor) and correspond*).

It was challenging to strike an appropriate balance between search sensitivity and specificity. Test search strings often included what we considered too many or too few publications. The main search string was:

“(bias) AND (tool OR assess*) AND (Cochrane)”

To increase the sensitivity, we performed additional searches (with the identified keywords and synonyms in the most beneficial combinations). We used the ”sorted by relevance”-function in PubMed and Google Scholar, and stopped screening, evaluating and assessing publications when no “major/minor comments” (or “peripheral remarks”) had been identified in a substantial amount of the sorted publications (>100 references). Furthermore, we retrospectively searched additional comments from the most active commenters. Many were members of The Cochrane Bias Methods Group so we searched additional comments from the remainder of the Group assuming they would be interested in commenting on the Cochrane risk of bias tool for randomized clinical trials. Most of the assessed full-texts did not comment on the tool (but were systematic reviews that used the tool or publications that described/explained i.e. the basis of the tool). We decided that a full text assessment of approximately 1000 publications would constitute a fairly thorough search (we assessed 976 full-texts).

**Appendix 2: Analysis of use of the Cochrane risk of bias tool for randomized clinical trials: selection of Cochrane and non-Cochrane reviews**

1: We included 100 Cochrane reviews via PubMed with the following search string:

”The Cochrane database of systematic reviews”[Journal] AND (systematic[sb] AND (”0001/01/01”[PDAT]: ”2014/12/31”[PDAT]))

15 Cochrane reviews were excluded to reach 100 Cochrane reviews: 12 Cochrane reviews did not include any randomized clinical trials in their analysis, two had been withdrawn and one did not involve a clinical intervention. About half of the included Cochrane reviews were 1^st^ edition Cochrane reviews (45/100, 45%) and about half were updates of previous published Cochrane reviews (55/100, 55%). Most updates were 2^nd^ edition Cochrane reviews (33/55, 60%), some were 3^rd^ edition (16/55, 29%) and few were 4^th^ (5/55, 9%) and 5^th^ (1/55, 2%) editions.

2: We included 100 non-Cochrane reviews from PubMed with the following search string:

(((systematic[sb]) AND (”0001/01/01”[PDAT]: ”2014/12/31”[PDAT])) AND (systematic[Title/Abstract] OR systematical[Title/Abstract] OR systematically[Title/Abstract])) NOT ”The Cochrane database of systematic reviews”[Journal]

65 non-Cochrane reviews were excluded to reach 100 non-Cochrane reviews: 57 non-Cochrane reviews did not include any randomized clinical trials and eight non-Cochrane reviews were not retrievable through The Royal Danish Library. All of the included non-Cochrane reviews were 1^st^ edition reviews (100%) and none were updates of previous published reviews or published as Cochrane reviews.

**Appendix 3: Comments on the Cochrane risk of bias tool for randomized clinical trials**

Table I: Major comments on the Cochrane risk of bias tool for randomized clinical trials

| **Reference** | **First author** | **Key findings from comments** | **Selected commentaries from references** | **Wordcount** |
| --- | --- | --- | --- | --- |
| 1 | Armijo-Olivo | Strengths:  …  Challenges:  Many “high/unclear risk” trials  Insufficient tool domains  Poor risk of bias agreement  Suggestions:  Need for improved guidelines and empirical evidence supporting additional domains  Perform risk of bias for (main) outcomes | “Can we trust risk of bias results reported in Cochrane reviews? Can we trust assessments using the risk of bias tool?”...”The large number of trials classified as high or unclear risk of bias casts doubts about the discrimination power of the risk of bias tool.”...”In addition, the items included in the risk of bias may be insufficient to represent the construct of interest: “Risk of bias”. Other items not considered in this tool may need to be added to provide a more comprehensive evaluation.”...”Empirical evidence supports the evaluation of randomization, allocation concealment and blinding of clinical trials, all of which are included in the risk of bias tool. While there is insufficient evidence to support other domains being included, other methodological factors could be important for evaluating risk of bias and could be considered for inclusion in the risk of bias tool after careful empirical evidence testing.”...”Poor agreement [using the risk of bias tool] was not only demonstrated at the trial level but also at the meta-analysis level.”...”Improved guidelines to apply the risk of bias tool and revisions to the tool for different health areas are needed. In addition, empirical evidence supporting additional items for the risk of bias tool needs to be developed.”…“The majority of Cochrane reviews analyzed did not clearly specify the outcome used for risk of bias assessments. This directly reduces reproducibility of risk of bias assessment for outcome-dependent domains of the tool. Cochrane reviewers should report risk of bias assessments separately for each outcome analyzed, or at least for the main outcomes of the review.” | 259 |
| 2 | Bero | Strengths:  …  Challenges:  The tool does not include funding  Suggestions:  Include funding in the tool | “The current Cochrane risk of bias tool is insufficient to assess bias related to study funding sources.”...”Those opposed to adding funding source to the Cochrane risk of bias tool argue that all mechanisms of bias can be identified, perfectly measured, and incorporated quantitatively in the results of meta-analysis.”...”The Cochrane risk of bias tool should include funding source as a standard item because: 1. Funding source fits the definition of bias, 2. There is empirically-based evidence of bias related to funding source, 3. The observed bias related to funding source cannot be captured by the risk of bias criteria currently assessed with the risk of bias tool, 4. Risks of bias are not mutually exclusive, 5. Bias may be related to funding source even when all studies are industry-funded.” | 128 |
| 3 | Boutron | Strengths:  Aim of transparency  Based on empirical evidence  An important step forward in risk of bias assessment  Challenges:  Reliabilty: risk of bias assessment is intertwined with quality of reporting  Interrater variability is high  Suggestions:  Integrate Akl’s blinding assessment approach in the tool | “[The tool] aims at being completely transparent, with a separation of the facts and reviewers’ judgments. This aim is particularly important because reviewers, editors, and readers can challenge the author on the judgment.”...”[The tool] domains were chosen on the basis of empirical evidence demonstrating potential for bias or exaggeration of treatment effects [5], [6], [7], [8] and [9].”...”This tool has been an important step forward in the assessment of the risk of bias in systematic reviews and meta-analyses. Assessing the risk of bias with the risk of bias tool and evaluating the quality of studies included in systematic reviews raizes some challenges mainly related to reliability. The assessment of the risk of bias of a trial is intertwined with the quality of reporting.”...”Akl et al provide a useful tool to improve the assessment of the blinding status. They validated their tool by contacting authors of the published trials. Of course, this is only a preliminary step in evaluating the risk of bias because it cannot help determine whether blinding was efficient and evaluate the risk of bias when blinding is not implemented.”...”Furthermore, the validation relying on contacting authors can be debated [20]. However, the authors’ approach is interesting and could be used for other items of the risk of bias tool. This assessment could be helpful to improve interrater agreement and transparency when assessing the risk of bias with the risk of bias tool.” | 234 |
| 4 | De Bruin | Strengths:  …  Challenges:  The tool seems to be mostly based on non-behavioural trials  Risk of bias assessments are insufficiently included in analysis even when done  Suggestions:  Evaluate the success of non-conventional blinding measures | “This paucity of research on risk of bias in HBC [health behaviour change] trial is in turn reflected in widely used instruments for assessing the risk of bias, such as the Cochrane risk-of-bias tool (Higgins et al., 2011), which seems to be mostly based on evidence from non-behavioural trials.”...”...the question–behaviour effect [...] is one of these sources of bias that seems specific to the evaluation of educational, psychological or behavioural (i.e. non-pharmacological) interventions, and may not be adequately captured by commonly used risk-of-bias tools.”...”Given that a previous meta-analysis of HBC trials in which one of the authors was involved (Dr. Viechtbauer, in de Bruin et al., 2010) revealed that attrition may not be random (i.e. patients doing better were more likely to dropout), differential attrition might indeed be an important source of bias in HBC trials.”...”…systematic reviewers could consider adapting the risk-of-bias tool to the literature under review: some common measures like blinding participants to group assignment might not be possible, but alternative or additional measures might be taken to reduce the risk of bias in these trials and it may be worthwhile considering (or evaluating the success of) these.”…“Based on a survey of 200 meta-analyses, they arrive at the conclusion that many do assess methodological quality, but very few incorporate them in their analyses. Johnson and colleagues were particularly interested in whether methodological quality was used in moderator analyses (‘an interactive approach’), e.g. when examining whether a particular behaviour change technique or modality of intervention delivery explains heterogeneity in effect sizes. They find that this is rarely reported by the meta-analyses reviewed, but when it is reported the moderator results remained significant in higher quality studies, or were present among higher but not lower quality studies. Johnson and colleagues also make the important argument that instead of discarding trials that score lower on certain methodological quality criteria (i.e. assuming that this affects the evidence-base), it is much more informative to include all trials in the meta-analyses and actually establish whether and how methodological quality influences trial results.” | 337 |
| 5 | Hartling | Strengths:  The tool is based on methods – not reporting  The tool is based on empirical evidence  Challenges:  Low agreement between reviewers  Many “high/unclear risk” trials – The tool might not be sensitive enough and the “unclear” category might become the default  No differences in effect sizes across risk of bias categories (high, unclear and low)  Non-parallel design and non-pharmacological intervention creates ambiguity in risk of bias assessment  Consensus-rating is better than two-reviewer rating  Suggestions:  Need for renewed guidelines (regarding interpretation and application), decision rules, transparency and additional testing.  Make a “living database” with examples and consensus from experts | “Low agreement between reviewers suggests the need for more specific guidance regarding interpretation and application of the Risk of Bias tool (RoB) or possibly re-phrasing of items for clarity.”...”Examination of study-level variables and their association with inter-rater agreement identifies areas that require specific guidance in applying the Rob [Risk of Bias] tool. For example, nature of the outcome (objective vs. subjective), study design (parallel vs. other), and trial hypothesis (efficacy/superiority vs. other).”...”Low agreement between pairs of reviewers indicates the potential for inconsistent application and interpretation of the RoB [Risk of Bias] tool across different groups and systematic reviews.”...”Post hoc analyses showed that disagreement most often arose from interpretation of the tool rather than discrepancies in the information that was extracted from studies.”...”The majority of trials in the sample were assessed as high or unclear risk of bias for many domains, likely due to inadequate reporting at the study level. This raizes concerns about the ability of the RoB [Risk of Bias] tool to detect differences across trials that may relate to biases in estimates of treatment effects.”...”No statistically significant differences were found in effect sizes (ES) across high, unclear and low risk of bias categories.”...”the low agreement raizes concerns and points to the need for clear and detailed guidance in terms of applying the RoB [Risk of Bias] tool.”...”...agreement for some domains may be better in classic parallel trials of pharmacological interventions, whereas trials with different design features [...] and those examining nonpharmacological interventions appear to create more ambiguity for risk of bias assessments.”...”...the consensus rating is a more meaningful measure of agreement (as opposed to reliability between two reviewers), as these ratings are the ones reported in systematic reviews.”...”Of particular note is that 99 percent of this sample had overall risk of bias assessments as high or unclear. This is similar to three of the four other samples that had more than 90 percent assessed as high or unclear risk of bias overall.”...”If the vast majority of trials are assessed as high or unclear risk of bias, the tool may not be sufficiently sensitive to differences in methodology that might explain variation in treatment effect estimates across studies, or study methodology as a potential explanation for heterogeneity in meta-analyses.”...”While the focus of the RoB [Risk of Bias] tool is intended to be on methods rather than reporting, reviewers regularly indicate that they rely on the trial reporting to make their assessments.”...”We do not intend for our results to suggest that reviewers abandon existing tools for other tools unless these have shown greater reliability and validity. Rather, our results underscore the need for reviewers and review teams to be aware of the limitations of existing tools and to be transparent in the process of risk of bias/quality assessment. Detailed guidelines, decision rules, and transparency are needed so that readers and end-users of systematic reviews can see how the tools were applied. Further, pilot testing and development of review-specific guidelines and decision rules should be mandatory and reported in detail.”...”There is a need for more detailed guidelines to apply both the RoB [Risk of Bias] tool and the NOS, as well as revisions to the tools to enhance clarity. Additional testing should occur after further revisions to the tool and when expanded guidelines are available.”...”A final caveat to note is that the RoB [Risk of Bias] tool has undergone some revisions since we initiated the study. These are detailed in the most recent version of the Cochrane Handbook11 but were not incorporated into our research. The changes affected primarily the blinding and the “other sources of bias” domains. This does not impact the general findings from our research; however, further testing with the modified tool is warranted.”…”The developers of the tool aimed to distinguish between actual methods of conducting the trials vs. reporting. Furthermore, the choice of components for inclusion in the tool was based on the empirical evidence demonstrating their association with effect estimates.” | 637 |
| 6 | Hróbjartsson | Strengths:  The tool has a standardized approach, based on theoretical and empirical grounds  Challenges:  Cochrane review authors are often reluctant regarding overall risk of bias assessment and incorporation of risk of bias in analyses’  Risk of confounded risk of bias evidence base  Based on observational studies  Unclear if funding, centre-status, early stopping and country are unique biases  Unclear if the tool addresses funding adequately  Suggestions:  … | “The risk of bias tool provides a standardized approach, based on items selected on both theoretical and empirical grounds, and following broad consultations with clinical research methodologists.”...”The risk of bias tool is a comparatively recent development that still likely needs refinement. The modest inter-rater agreement rates[5] will hopefully be improved by modifications to the questions and enhanced training courses. However, authors of Cochrane Reviews tend to be reluctant in designating an overall risk of bias for each trial or outcome and also reluctant to incorporate the risk of bias assessment in analyses and conclusions.”...”risk of bias assessment has more fundamental challenges.”...” The risk of confounding in [meta-epidemiologic studie] comparisons is pronounced, as compared trials may differ for other reasons.”...”The assessment of risk of bias is to a large extent based on common sense and theoretical considerations, with an empirical basis of observational studies with a considerable risk of confounding.”...”the empirical evidence underlying the assessment of risk of bias in trials – an assessment necessary for ensuring that biased trials do not lead to biased systematic reviews – is based on observational studies.”...”An increasing number of meta-epidemiological studies report associations between a trial characteristic and exaggerated treatment effects: funding status,[7] number of centres participating in a trial,[8] early stopping of a trial,[9] and developing country status.[10] For many of these characteristics it is unclear whether they represent a unique bias, confounding, publication bias, spurious findings, or a combination of these and/or other unknown factors. It is, nonetheless, helpful to be aware of such associations, sometimes called meta-bias.[11]”...”Funding status is a major concern in randomized trials. The exaggerated effects reported for industry trials [7] may to some extent be explained as a result of publication bias or other characteristics included in the risk of bias tool, for example selective outcome reporting. However, companies that stand to gain financially by a positive result have substantial conflicts of interest when they control the planning, funding, conduct, and reporting of a trial. It is not clear that the risk of bias tool in its present version addresses this problem adequately.” | 344 |
| 7 | Ivers | Strengths:  …  Challenges:  The tool does not capture all sources of methodological bias  Poor reporting interferes with assessment of risk of bias domains  One domain with “high” risk leads to overall “high” risk  The tool only catches risk of bias and not other methodological flaws  Suggestions:  Use sensitivity analyses for baseline and follow up | “The risk of bias tool does not capture all sources of methodological bias and poor reporting interferes with the assessment of many domains.”...”…we found that nearly half of RCTs [randomized clinical trials] focusing on diabetes had at least one domain at high risk of bias…[and]…that poor follow-up, baseline imbalances and blinding were the most common sources of high risk of bias.”...”…sensitivity analyses may be used to explore the risk of bias related to loss of follow-up, and risk of baseline imbalances in [quality improvement] trials may be reduced through restricted randomisation techniques, especially when trials are cluster-randomized with relatively few clusters.”...”...it is possible that studies at low risk of bias have important flaws with respect to methodology and/or reporting (and vice versa), and it is possible that using other scales to assess study quality could have led to different results.14 While the overall risk of bias assessment using the Cochrane Risk of Bias tool has been shown to differentiate effect sizes (ie, higher risk of bias studies usually have larger effect sizes),10 studies at high risk of bias may still offer valuable knowledge...”...”Furthermore, we acknowledge that assigning trials with high risk of bias in a single domain a status of high risk of bias overall may be arguable.”…”Some important components of methodological quality do not relate to bias (eg, reporting of a sample size calculation). Thus, it is possible that studies at low risk of bias have important flaws with respect to methodology and/or reporting (and vice versa), and it is possible that using other scales to assess study quality could have led to different results.” | 265 |
| 8 | Jefferson | Strengths:  Facilitates the process of critical assessment of a clinical trial  Challenges:  The tool does not identify all important study design bias-variables, nor organizes data or checks data-coherence  Each domain may have more than one source of bias application  The tool is difficult to apply due to its checklist approach and relative subjectivity  Risk of bias assessment is time consuming  Suggestions:  Need for further tool development  The tool should be developed so that Cochrane reviews may rely on clinical study reports as the basic unit of analysis | “The current Cochrane risk of bias tool is not adequate for the task as it does not reliably identify all types of important biases, and nor does it organize and check the coherence of large amounts of information.”...”...each [the tool domain] may have more than one source of bias application...”...”In addition, the current Cochrane risk of bias tool was introduced after the production of our review of published articles, making the comparison, had we had the data to undertake it, more difficult to interpret and possibly unfair.”...”We found the Cochrane risk of bias tool to be difficult to apply to clinical study reports. We think this is not because the tool was constructed to assess journal publications but, as with all list-like instruments, its use lends itself to a checklist approach (in which each design item is sought and, if found, eliminated from the bias equation rather than with thought and consideration).”...”Many of the variables we found to be important when assessing the trial (eg, date of trial protocol, date of unblinding, date of participant enrolment) are simply not captured in the risk of bias tool when used in a routine way or to review publications. We were also often unsure how to judge the risk of bias when bias itself can actually or potentially be measured with reviewers’ access to full clinical study reports and individual participant data. If, for example, the original trial protocol is available, one can judge whether reporting bias occurred. Reviewers need not guess at bias (ie, make a judgement of ‘risk’) but can judge bias directly. However, even with individual participant data, some forms of bias, such as attrition bias, may still be difficult to quantify, and one can only judge the risk (ie, potential) of bias.”...”While the judgements of ‘low’ or ‘high’ risk of bias may imply certainty, particularly when based on the reading of a full clinical study report, we found ourselves often in lengthy debate and discussion over the proper level of risk of bias before arriving at a consensus. We found the risk of bias judgements themselves to carry a high level of subjectivity, in which different judgements can be justified in different ways. The real strength of the risk of bias tool appears not to be in the final judgements it enables, but rather in the process it helps facilitate: critical assessment of a clinical trial.”…”Reviewing the complete clinical study reports and our assessment of bias was very time consuming.”...”…the current Cochrane risk of bias tool does not sufficiently identify possible faults with study design, and nor does it help to organize and check the coherence of large amounts of information that are found in clinical study reports. Our experience suggests that more detailed extraction sheets that prompt reviewers to consider additional aspects of study may be needed. Until a more appropriate guide is developed, we offer our custom extraction sheets to Cochrane reviewers and others interested in assessing risk of bias using clinical study reports and encourage further development.”…”As evidence of reporting bias in industry trial publication mounts, 8 16–21 we believe Cochrane reviews should increasingly rely on clinical study reports as the basic unit of analysis.” | 533 |
| 9 | Katikireddi | Strengths:  …  Challenges:  Risk of bias should be outcome-specific, informed by empirical evidence and the direction and magnitude of bias  Reviewers struggle to understand and operationalize current guidance on how to conduct and incorporate risk of bias assessment into synthesis of data  Some review authors do not consider risk of bias for individual outcomes  Suggestions:  Need for research on bias importance and impact, and for optimized guidelines  Risk of bias assessment should be incorporated in study synthesis and conclusions | “These [/the tools] judgements should be outcome-specific and ideally informed by empirical evidence of bias, the likely direction of bias and the likely magnitude of bias. However, this may be difficult to achieve in practice, given the acknowledged evidence gap in the relative importance of different domains of bias.4”...”Incorporation of risk of bias assessments into synthesis is crucial to ensure that SR conclusions are based on the best available evidence. Failure has serious implications for evidence-informed policy and practice.”...”Assessing risk of bias using scoring systems continues, despite the Cochrane Collaboration's recommendations to avoid their use.”...”Approaches to incorporating risk of bias assessments into the findings of SRs [systematic reviews] are (arguably appropriately) varied but frequently lack transparency. Lastly, some SRs that investigate multiple outcomes continue to ignore the potential for risk of bias to differ across outcomes.”...”...many Cochrane SRs [systematic reviews] selectively followed some but not all of the recently published guidance.”...”Half of the reviews published in the sample from the Cochrane library and leading general medical journals did not incorporate findings of critical appraisal into their review.”...”Our findings not only show that risk of bias summary scores are still frequently used but also suggest that there is confusion about how best to incorporate critical appraisals into SR [systematic reviews] findings.”...”reviewers should clearly report findings from the most robust studies, either as a sensitivity analysis or in the primary analysis.”...”Given that the study design and type of intervention are closely related, reviewers should ideally go further and consider whether an ‘intervention selection bias’ is inadvertently introduced by focusing only on higher quality studies.”...”Early tools for critical appraisal were appealing because they were simple to use and resulted in a score which allowed ranking of studies by risk of bias, facilitating incorporation into the synthesis process.17 ,19 ,35 Unfortunately, this simplicity came to be regarded as a source of weakness, as well as a strength, and these tools have been replaced by more complex guidance intended to address some of their limitations.4 ,21 ,36 We recognize the need for this development but take the view, supported we believe by this study, that reviewers are struggling to understand and/or operationalize current guidance on how to conduct and incorporate critical appraisal within synthesis. Further research is required to establish the relative importance of different forms of bias and their likely impact6, 37 and also to clarify how critical appraisals should be incorporated into SR findings 38, 39. However, to ensure that SRs [systematic reviews] really do direct decision-makers to the best available evidence, there is an urgent need to make guidance more understandable to the diverse reviewers involved.”…”Lastly, some SRs [systematic reviews] that investigate multiple outcomes continue to ignore the potential for risk of bias to differ across outcomes.” | 456 |
| 10 | Morissette | Strengths:  The risk of bias terminology “high/low” risk  The tool focuses on bias instead of study quality and is transparent in its assessments  Challenges:  Might take longer than other tools  Suggestions:  Need for further research on the tool i.e. if blind or non-blind risk of bias assessment is best | “...if blinded assessments are associated with less bias, then this approach needs to be part of the systematic review process.”...”Potential confounding factors that we were unable to examine included the assessors’ level of experience with risk of bias assessments.”...”The risk of bias deals with the extent to which a study's results are believed to be true and attempts to focus on how the study was actually done.”...”...the risk of bias terminology overcomes rating a study as being 'low quality' when it may not be feasible (or appropriate) to conduct a particular methodological component (e.g. blinding).”...”...we encourage further research in this area and recommend using all of the important components of the Cochrane 'Risk of bias' tool.”…”The Cochrane 'Risk of bias' tool differs from other quality appraisal tools because it questions the degree to which a study’s results should be believed, is based on other documents above and beyond the study report (e.g. study protocol), and the risk of bias appraisal decisions are recorded to ensure transparency (Hartling 2009).” | 168 |
| 11 | Moustgaard | Strengths:  …  Challenges:  Lack of explicit characterization and classification of subjective and objective outcomes for risk of bias assessment – also in relation to co-interventions  Central methodological terms are understood differently  High interrater variability  Suggestions:  The Cochrane Handbook should explain what is meant by objective and subjective outcomes | “The classification of outcomes as subjective or objective is central to formal and informal assessments of the risk of bias in individual trials.”...”The terms “subjective outcomes” and “objective outcomes” occur repeatedly in the Cochrane Handbook in connection with assessment of risk of bias in clinical trials, but we neither found explicit characterization of the terms (The Cochrane Handbook was included in the review because of an explicit description of the category “patient reported outcomes”) nor is “subjective outcome” or “objective outcome” defined in the online Cochrane Glossary.”...”Many of the passages in the Cochrane Handbook that include the terms “subjective” or “objective” outcomes concern the risk of detection bias, that is, systematic differences between groups in how outcomes are determined.”...” No characterization of subjective vs. objective outcomes relevant to risk of performance bias is given explicitly in the Cochrane Handbook nor did we find it in the methodological articles or the clinical trial reports we reviewed.”...”all outcomes may potentially be affected by cointerventions and other differences in basic care and the distinction between subjective and objective outcomes thus seems less relevant when discussing risk of bias because of cointerventions. Still, these reflections are speculative, and in the context of performance bias, the meaning of “subjective” and “objective” outcomes remains somewhat obscure.”...”The Cochrane risk of bias tool has been found to have a moderate interobserver agreement rate [21]. Interobserver agreement will hopefully improve once assessors become more familiar with the tool, are offered better training, and the tool is refined. One likely contributing factor to the modest agreement rates is the different interpretations of what is meant by subjective and objective outcomes.”...”It should also be noted that some outcomes that have generally been considered “objective” may in fact leave considerable room for judgment by the assessor, for instance, blood pressure, ultrasonic measurements, radiographic outcomes, and so on.”...”Our study indicates that central methodological terms may be understood differently by different readers, and we suggest that authors of clinical trial reports explicitly explain the intended meaning of the terms “subjective” and “objective” outcomes (as well as other terms that may be considered ambiguous). Journal editors might contribute to greater clarity by requiring that authors provide such explicit explanations.”…”We also suggest that adding an explicit clarification of the intended meaning of ‘‘subjective’’ and ‘‘objective’’ outcomes in future versions of the Cochrane Handbook might further strengthen its important role in guiding review authors.” | 399 |
| 12 | Roseman | Strengths:  The Cochrane Handbook mentions that funding should be collected  Challenges:  Inconsistent and non-specific recommendations for including funding in the tool  The Cochrane Collaboration should reconsider its position that trial funding and trial author-industry financial ties not be included in the risk of bias assessment  Most Cochrane reviews in 2010 did not report funding  Suggestions:  Cochrane reviews should report the funding sources of all included trials  Include conflicts of interest in the risk of bias assessment | “The 2008 edition of the [Cochrane] handbook suggested that potential bias related to the influence of trial sponsors could be considered in an optional “other sources of bias” domain of the risk of bias tool.37 In contrast, the 2011 edition specifies that this information should not be incorporated in the risk of bias assessment.38 Both versions of the handbook mention that review authors may consider extracting data on trial author-industry financial ties but do not specify if and where this information should be reported.33, 34.”...”Given the well documented influence of industry funding of drug trials on their conduct, interpretation, and reporting,1 4 5 6 7 8 9 10 12 the Cochrane handbook and the PRISMA statement should be updated to require authors of systematic reviews and meta-analyses to report the funding sources of all included trials or to report that trial funding sources were not disclosed.”...”...if the funding source of included trials is only partially reported, readers might assume that the funding sources of other trials were available but not recorded, leaving them unsure as to how to interpret potential bias related to the funding sources of those trials.”...”Beyond study funding, consumers of research consider conflicts of interest from trial author-industry financial ties and employment as relevant to appraising the likelihood of bias in trials, 45 46 47.”...”...we recommend that the Cochrane Collaboration reconsider its position that trial funding and trial author-industry financial ties not be included in the risk of bias assessment.”...”One concern might be that including conflicts of interest from included trials in the risk of bias assessment could result in “double counting” of potential sources of bias. However, ratings in the risk of bias table are not summed to a single score, and inclusion of risk of bias from conflicts of interest could reflect mechanisms through which industry involvement can influence study outcomes6 that are not fully captured by the current domains of the risk of bias tool.”...”Inclusion of conflicts of interest from included trials in the risk of bias assessment would encourage a transparent assessment of whether industry funded trials and independently conducted trials reach similar conclusions. It would also make it explicit when an entire area of research has been funded by industry and would benefit from outside scrutiny. Coding trial funding sources can be complex, and it may not always be clear to what degree different funders played a part in a given study.”...”A reasonably simple system would be to code trial funding as pharmaceutical industry, non-industry (for example, public granting agency, private not for profit granting agency), combined pharmaceutical industry and non-industry, non-industry with study drug supplied by pharmaceutical industry, no study funding, or not reported.22.”...”Most Cochrane reviews of drug trials published in 2010 did not report information on trial funding sources or trial author-industry financial ties, including employment, from included trials...”...”...Cochrane and the PRISMA statement should require reviews to report conflicts of interest from included trials in a way that is consistent across reviews, and Cochrane should include this information as part of risk of bias assessment.” | 505 |
| 13 | Savović | Strengths:  The tool has a standardized approach, ensures transparency, is flexible and is a platform for critical thinking  Challenges:  Difficult to assess some risk of bias domains - especially incomplete outcome data and selective reporting  Wide approach to the “other bias” domain  Increased workload and complexity compared to past practice  Suggestions:  Need for better training and guidance materials for judgements and on how to incorporate risk of bias assessments into Cochrane reviews  Graphical representation should be for individual outcomes  Need for more research into empirical foundation of domains | “The domains reported to be the most difficult to assess were [...] incomplete outcome data and selective reporting of outcomes. There was wide variation in how review authors had approached the ‘other bias’ domain, with a lack of clarity over what additional items should be considered here. Some of the items that authors have included (such as sample size calculations and funding source) are explicitly discouraged in the Cochrane Handbook guidance. While there is evidence that some factors are empirically associated with effect estimates, such as single versus multicentre design, early stopping of trials and funding source [14-16], the extent to which these should be considered alongside the main bias domains is still a topic of debate. The evaluation highlighted a need for more and better training and guidance materials, such as algorithms or similar structured guidance for reaching domain-level judgements, as well as guidance on how to incorporate risk of bias assessments into meta-analyses and review conclusions. Recommendations for changes or further developments were made based on identified needs and many have already been incorporated into the new edition of the Cochrane Handbook, while other developments are underway. As suggested by evaluation participants, an online bank of worked examples for risk of bias assessments will be incorporated into future versions of the Cochrane Handbook or made available online.”...”…the main purpose of this evaluation was to identify potential problems with the risk of bias tool that can be rectified, and we suspect that users who encountered problems are more likely to have responded.”...”…it is possible that the number of people incorrectly applying these concepts may be higher as authors may be unaware of their misunderstandings.”...”It is important that guidance and training materials continue to be developed for all aspects of the tool.”...”...44% or more of respondents had difficulty with assessing each of the individual risk of bias domains.”...”…clearer guidance, ideally based on empirical evidence, is needed on how to deal with studies at high risk of bias in meta-analyses, other syntheses of evidence across studies and drawing conclusions.”…”Specific benefits described included: having a standardized approach to bias assessments; the transparency provided by requesting quotes; the flexibility of the tool; […] providing a good framework for consideration of the risk of bias; and providing a platform to encourage critical thinking.”…”The main drawbacks described which were also addressed in the survey, included: the increased workload and complexity as compared with past practice.”…”For example, several participants raized the issue that risk of bias assessments present a particular problem when updating systematic reviews. Adopting the new tool in an updated review requires review authors to reassess the risk of bias of studies included in the original review, which they were often unwilling to do, and Cochrane Review Groups were not resourced to do this on behalf of authors.”…” Participants also suggested that graphical displays of risk of bias assessments across studies should be prepared separately for individual outcomes measured in the review rather than at the study level, as individual outcomes can be judged to be at higher or lower risk of bias using the tool.”…“More empirical evidence is needed to further inform considerations of what methodological aspects are most important in assessing risk of bias. There is a particular need for assessment of the influence of participant attrition on effect estimates, and on separate contributions to bias from blinding of patients and caregivers versus blinding of outcome assessors.” | 563 |
| 14 | Sterne | Strengths:  The tool does not include funding  Challenges:  The tool does not work well for assessment of selective reporting  Suggestions:  Transparent inclusion of “conflict of interest” | “Little evidence that: – Trial methods are more likely to be flawed if a trial is industryfunded. – Fraud is more likely if a trial is industry-funded.”...”The problems are (mainly) with selective reporting of outcomes, non-reporting of whole studies, and choice of comparator.”...”The current risk of bias tool does not work well for assessment of selective reporting.”...”The fight to access all trial data is of fundamental importance to the Collaboration and to evidence-based health care, but the Cochrane risk of bias tool should not include funding source as a standard item.”…”Transparent and structured assessments of conflict of interest in included trials should be a routine component of Cochrane systematic reviews. Such assessments relate mainly to the context in which the review results should be interpreted. It is vital to know whether most of the information comes from a company with a commercial interest in the intervention of interest, but this is not in itself a reason to dismiss the accumulated evidence. It may nonetheless be a reason to search particularly hard for unreported or selectively reported evidence and to carefully scrutinize the chosen comparators.” | 182 |
| 15 | Vale | Strengths:  …  Challenges:  The tools ability to measure the true bias is limited  Assessing risk of bias is difficult for the more subjective domains  Many “high/unclear risk” trials  Lack of risk of bias-guidance for trials with inadequate information  The tool relies on reporting  Suggestions:  … | “The Cochrane Handbook4 states that because the ability to measure the true bias (or even the true risk of bias) is limited, then the possibility to validate a tool to assess that risk is also limited. Nevertheless, authors of Cochrane systematic reviews are required to use the Cochrane risk of bias tool.”...”...our study has gone some way towards validating the Cochrane risk of bias tool, since access to the additional information and data enables us to get closer to the true risk of bias of individual studies.”...”Assessing risk of bias was particularly difficult for the more subjective domains [...] discrepancies were more common for attrition bias...”...”...we applied the risk of bias assessment to overall survival—an objective outcome that is commonly well reported—rather than considering all possible outcomes as is recommended. Our results could have therefore represented an optimistic view of the reliability of the risk of bias assessments using published information alone, particularly in relation to incomplete outcome data and selective outcome reporting.”...”…even with additional information, around a third of the included studies were classified as unclear risk of bias. Clearly, forms purposely designed to collect specific information would help reviewers reach appropriate judgments regarding risk of bias, in particular for those trials with inadequate information published.”…”The vast majority of Cochrane (and other) systematic reviews is based on information extracted from the publications of eligible studies. Therefore, most risk of bias assessments are similarly based on trial publications. However, trial quality is not necessarily well represented in publications.” | 251 |

Table II: Minor comments on the Cochrane risk of bias tool for randomized clinical trials

| **Reference** | **First author** | **Key findings from comments** | **Selected commentaries from references** | **Wordcount** |
| --- | --- | --- | --- | --- |
| 16 | Abou-Setta | Strengths: …  Challenges: Many “high/unclear risk” trials Sensitivity/specificity of the tool  Suggestions: … | ”The domain most often assessed as 'low' risk was 'incomplete outcome data'; 'blinding' was most often assessed as 'high' risk.”…”…97% of trials were assessed as 'unclear' or 'high' risk which raizes questions regarding the sensitivity/specificity of the risk of bias tool.” | 42 |
| 17 | Akl | Strengths: …  Challenges: The Cochrane handbook does not provide specific guidance on how to make “blinding” judgments Due to poor reporting, many unclear risk  Suggestions: The use of “probably yes” and “probably no” can enhance the assessment of blinding | ”The Cochrane risk of bias tool requires quoting what was reported to have happened in the study and then assigning a judgment relating to the risk of bias for each risk of bias item.”...”Although the Cochrane handbook suggests supplementing an ambiguous quote with either a “probably done” or a “probably not done” statement, it does not provide specific guidance on how to make this judgment.”...”We invite the Cochrane Collaboration [...] to consider using our structured [blinding] approach.”...”...the use of “probably yes” and “probably no” can enhance the assessment of blinding...” | 88 |
| 18 | Anette-Blümle | Strengths: …  Challenges: Risk of bias assessment is complicated and often based on incomplete reporting The tool is very difficult to validate  Suggestions: … | ”To estimate the methodological quality of studies instruments such as the Cochrane Risk of Bias tool were developed. But it can only be appraized what is reported.”...”The evaluation tool that the Cochrane Collaboration recommends is neither a scale nor a checklist. It is an instrument by which the risk of bias of individual methodological aspects can be evaluated. Since it is impossible to determine the actual risk of bias in a study, it is very difficult to validate an assessment tool.” | 81 |
| 19 | Armijo-Olivo  (2^nd^ included publication by this author) | Strengths: Appropriate that the tool comprehensively evaluates randomization and blinding  Challenges: The tool might not be able to adequately discriminate the comparative quality of included studies Need for futher validation of the tool The information needed in the tool is often poorly reported  Suggestions “Validity and reliability of data collection methods” and “confounders” could be considered in the tools “other bias” Review groups should customize the tool for specific topics | ”Because The tool is designed only for evaluation of RCTs, the overall quality grades are weighted heavily on the judgements assigned to the two randomization domains (sequence generation and allocation concealment), as well as the domain relating to blinding. Given the crucial importance of these three elements to the risk of bias among RCTs [4], it seems appropriate that The tool comprehensively evaluates randomization and blinding, and that the scores of these domains constitute approximately 50 % of the final grade assigned by this tool.”...”The EPHPP [Effective Public Health Practice Project] grade takes into account the validity and reliability of data collection methods. This item is not directly assessed by The tool but could be considered in the context of other bias. Similarly, the presence of confounders is specifically evaluated by the EPHPP tool, but could be considered under the ‘other sources of bias’ domain of The tool. Conversely, The tool evaluates the completeness of follow-up and selective outcome reporting in much greater depth than the EPHPP.”...”This raizes the question of whether the tools [are] able to adequately discriminate the comparative quality of the included studies.”...”It is possible that this small sample of RCTs covering a single topic area were all of similar quality. We encourage other teams that work with The tool to test it under different scenarios and disciplines, to assess a range of studies of varying quality and topic areas. This would provide further evidence of its validity, a requirement if recommendations for clinical practice through Cochrane systematic reviews continue to be based on its assessment of study quality.”...”To address the subjective nature of judging several of The tool domains, we suggest that research groups fine-tune The tool guidelines to make them specific to the types of study under analysis.”…”Poor quality of reporting should theoretically affect our ability to complete both tools. However, we argue that the effect was pronounced with The tool, due to the greater depth of information required to assess each domain.” | 331 |
| 20 | Armijo-Olivo  (3^rd^ included publication by this author) | Strengths: …  Challenges: The [tools] evidence base is restricted and incomplete  Suggestions: … | ”…the [tools] evidence base is restricted and incomplete. Therefore, we recommend that research evidence be expanded to different health areas regarding the association among methodological factors (items used in quality tools) and their link to treatment estimates, especially those that involve complex interventions such as allied health areas and physical therapy.”...”Many of the tools used to evaluate methodological quality and risk of bias of health research have several items linked to reporting instead of conduct.7” | 78 |
| 21 | Armijo-Olivo  (4^th^ included publication by this author) | Strengths: …  Challenges: Need for guidelines in applying and interpreting the results of the tool  Suggestions: All items included should be based on empirical evidence | ”...quality of reporting is commonly used as a proxy for trial quality, which has complicated the construct of “quality”...”...”The number of items across quality tools is large; 130 and 48 items have been used by tools in general health and PT research”...”Some items are subjective, confusing, and lack a clear definition (e.g., subjects appropriate to study questions, discussion of bias resulting from non-blinding assessment).”...”The risk of bias tool is recommended by The Cochrane Collaboration. Some groups within the Collaboration have developed their own tools and have not yet adopted the risk of bias approach (e.g. Cochrane Bone, Joint and Muscle Trauma Group). Other Cochrane groups have modified the risk of bias tool for their own purposes (i.e. Cochrane Back Review Group, Cochrane Renal Review Group). The risk of bias tool was developed more recently than many of the other tools; current research [9,13] recommends further testing of its psychometric properties and validation of the tool in a wide range of research fields. Additional guidelines will help users in applying and interpreting the results of the risk of bias tool.”...”The Jadad scale [37] is the most frequently cited tool in health sciences research despite criticisms regarding its lack of responsiveness [8] and applicability to other health research areas such as PT and rehabilitation [5].”...”Finally, items assessing the methodological quality (or internal validity) of RCTs should be based on empirical evidence of their association with treatment effects.” | 237 |
| 22 | Armijo-Olivo  (5^th^ included publication by this author) | Strengths: …  Challenges: The tool is not the gold standard Need for guidelines in applying and interpreting the results of the tool Many domains/components are yet to be investigated, so the evidence base is incomplete  Suggestions: … | ”There is no agreement regarding which tools are optimal to accurately determine trial quality. Most tools have not been developed using scientifically rigorous methods, lack reliability and/or have not been fully validated [15]. In addition, the use of different tools for evaluating the quality of primary research can lead to different end results [17-19]. Thus, a clinical trial may be rated on a quality scale disparate by different measurement tools. This discrepancy in the evaluation of the quality of research may skew interpretation, reporting, and as a result, could potentially impact recommendations for clinical care. Finally, the tools include different items, some of which relate more to the detail of reporting rather than methodological quality.”...”As a result of these shortcomings with existing tools and methods for quality assessment, there has been a shift in the traditional scoring approach to the assessment of trial quality. Instead of examining trial quality with tools that have not been validated and often use composite scores, the assessment of 'risk of bias’ was proposed in 2008 by the Cochrane Collaboration, one of the most important and influential groups working on evidence based practice worldwide [20].”...”The risk of bias tool includes...'other sources of bias’ (for example, early stopping for benefit, design-specific features such as adequate wash-out period in cross-over trials). Other methodological components within the risk of bias tool as well as other components that have traditionally been used to determine trial quality in health research have not been investigated; hence the evidence base is limited and incomplete. Furthermore, recent research [5,19] recommends further testing of the risk of bias tool to gain a better understanding of its psychometric properties, as well as to validate the tool in a wider range of research fields. Additional information will help users in applying and interpreting the results of the risk of bias tool.” – | 305 |
| 23 | Armstrong | Strengths: …  Challenges: The potential complexity of studies warrants investigation into factors beyond those explored in the Cochrane 'Risk of bias' tool  Suggestions: … | ”…for all included studies we will use a series of questions drawn from the Effective Public Health Practice tool for quality assessment to assess study internal and external validity (Effective Public Health Practice Project n.d). This approach is important as the potential complexity of studies warrants investigation into factors beyond those explored in the Cochrane 'Risk of bias' tool. Complexities include the increased likelihood that these studies will be prone to bias; and the importance of assessing and reporting on the external validity, fidelity and sustainability of interventions.” | 88 |
| 24 | Bafeta | Strengths: …  Challenges: Risk of bias domains are poorly reported  Suggestions: … | ”The risk of bias domains were poorly reported…”...”We found that more multicentre trials were at low risk of bias for the risk of bias domains of sequence generation and allocation concealment than single centre trials. After adjusting for each domain of the risk of bias tool as well as overall risk of bias, we obtained similar estimates with slightly wider confidence intervals.” | 62 |
| 25 | Barkhordaria | Strengths: …  Challenges: The subjective nature of the tool  Suggestion: … | ”Clearly, one among the major drawbacks of the Cochrane Risk of Bias tool is the subjective nature of its assessment protocol. In an effort to correct for this inherent weakness of the instrument, the Cochrane group produced detailed criteria for making judgments about the risk of bias from each individual item [16]. Moreover, Cochrane recommended that judgments be made independently by at least two people, with any discrepancies resolved by discussion [16]. This approach to increase the reliability of measurement in research synthesis protocols is akin to that described by us [19,20] and by AHRQ [21].” | 96 |
| 26 | Berkman | Strengths: The tool continues to evolve and improve  Challenges: Need for criteria for risk of bias consensus Different studies employ different definitions of the risk of bias approach which may be an important source of heterogeneity  Suggestions: … | ”...different studies employed different definitions of the approach to protecting against a bias.”...”The Cochrane tool continues to evolve and improve with respect to operationalizing criteria to adequately measure bias categories. A number of studies have evaluated the reliability and validity of the Cochrane tool and suggested improvements in the criteria and approach to assessing risk of bias in general.67, 69 These variations and ongoing improvements suggest that the different tools used to assess risk of bias are themselves a source of heterogeneity.” | 82 |
| 27 | Bero  (2^nd^ included publication by this author) | Strengths: …  Challenges: …  Suggestions: The tool should include funding Further empirical research on the association of industry funding with research outcomes is needed | ”I have argued that funding source should be considered a risk of bias [in the tool].”…”Further empirical research on the association of industry funding with research outcomes is needed in order to understand possible mechanisms for the observed industry bias in the context of different types of test drug or study design. In the meantime, the potential for industry bias should not be ignored.” | 64 |
| 28 | Buchberger | Strengths: The tool strengths include ease of use, short turnaround time, high transparency of the assessment and an easy to understand graphical representation of the results  Challenges: Other types of bias not included e.g. post hoc changes, intervention prior to randomization, insensitive instruments and fraud  Suggestions: … | ”The risk of bias (RoB) tool from the Cochrane Collaboration is an instrument for the assessment of the bias potential in controlled trials. Its strengths include ease of use, short turnaround time, high transparency of the assessment and an easy to understand graphical representation of the results.”...”The discussion on the inclusion of this bias in the risk of bias tool is ongoing [17, 18].”...”…the risk of bias tool of the Cochrane Collaboration stands out with high transparency, recognition of the principal types of bias, easy to use with only seven items and the most understandable graphical presentation of results.” | 99 |
| 29 | Cho | Strengths: …  Challenges: Many “high/unclear risk” trials The tool does not consider blinding exceptions  Suggestions: ... | ”When the tool is used to assess quality, allocation concealment, sequence generation and blinding items must all be satisfied for an article to be assessed with a lower risk of bias.”...”...studies that make blinding impossible are categorized as high-quality articles in the Jadad scale if they score two or more. However, the van Tulder scale and The tool do not consider such exceptions. We look forward to a consensus for new assessment tools that correct for these limitations in future discussions.” | 81 |
| 30 | Chung | Strengths: The tool is the most commonly used tool  Challenges: …  Suggestions: ... | ”...the two most commonly used tools being Cochrane risk of bias tool (44.8%) and Jadad Scale (36.3%).”...”tools for assessing risk of bias of primary studies Cochrane risk of bias tool 90 (44.8) Jadad Scale 73 (36.3) Others 26(12.9) Not reported 9(4.5) Two or more tools used 3 (1.5) Majority of MA reported potential sideeffects of CHM (86.6%).”...our data showed that risk of bias is more likely to be taken into account in conclusion formulation when there are a higher number of authors.” | 82 |
| 31 | Crocetti | Strengths: …  Challenges: The tool is subjective and not validated High disagreement  Suggestions: ... | ”Registered trials were 68 % less likely to have a high risk of bias for sequence generation, compared with nonregistered trials.”...”…the domain-based, risk-assessment tool developed by the Cochrane Collaboration is not a validated instrument. Even with prespecified definitions, we found a large amount of disagreement among reviewers, in that 53 % of studies required adjudication. These results [...] reflect the amount of subjective judgment inherent in assessment of some of the domains of the Cochrane Collaboration, risk-of-bias tool.” | 74 |
| 32 | Da Costa | Strengths: …  Challenges: Low risk of bias inter-agreement, perhaps due to poor training  Suggestions: Better training of reviewers | ”The risk of bias tool has been widely embraced by the systematic review community.”...”The selection of domains of bias was based on empirical evidence and theoretical considerations…”...”We recently found that the reliability of the risk of bias tool might be improved by intensive standardized training of raters.”…”…no formal evaluation of such a training intervention has been performed. We therefore aim to investigate whether training of raters, with objective and standardized instructions on how to assess risk of bias, can improve the within and between pairs of rater reliability of the Cochrane risk of bias tool.” | 95 |
| 33 | De Bruin | Strengths: …  Challenges: Risk of bias assessment is interrelated and impacts trial feasibility and external validity  Suggestions: tools (ie. the tool) should value alternative blinding strategies | ”Other sources of bias: Contamination and inappropriate administration, Stopping early or late for benefit: sample size adjustments, Scientific malpractice: the fabrication of data, the wilful distortion of data or results, and questionable research practices…”...”...we suggest that creative approaches to blinding and alternative strategies to blinding should be studied (do they work and under what conditions?), and that tools for assessing the risk of bias should consider valuing alternative strategies where blinding is not an option.”...”...it may not be realistic to capture...specific [blinding] strategies in general risk of bias assessment tools.” | 90 |
| 34 | Dechartres | Strengths: …  Challenges: The overall risk of bias on treatment outcomes is challenging and has not been validated Domains may not have the same risk of bias weight and may be associated with one another  Suggestions: Further research is needed to explore the overall risk of bias rating | ”Our results raize questions about the overall risk of bias, summarizing risk of bias across domains, as currently defined. The [CC] risk of bias tool includes methodological characteristics or domains shown to be associated individually with treatment outcomes in meta-epidemiologic studies.”...”Further research is needed to explore whether one can obtain a simple measure of the overall risk of bias for a given trial and, if so, how.”...”We also recommend assessing the influence on treatment outcomes of each domain of the risk of bias tool separately rather than summarizing these domains into an overall risk of bias.” | 96 |
| 35 | Faggion | Strengths: A domain-based tool (e.g. the tool) is preferable  Challenges: Susceptibility to bias may differ among domains  Suggestions: ... | ”...susceptibility to bias may differ among domains.”...”Authors should clearly state the objective of the assessment (risk of bias versus quality) and report separately and in detail other assessments related to overall quality. Authors should report in detail whether the methodological quality results were taken into account when MA was performed.”...”Because the assessment of the validity of a study may involve subjectivity (Higgins et al. 2011a), a domain-based tool (e.g. Cochrane's risk of bias tool) should be preferred. Domain-based tools have the flexibility of allowing authors to judge whether a specific threatened domain may in fact bias the estimate.” | 98 |
| 36 | Gordon | Strengths: The tool does not use risk of bias scores  Challenges: …  Suggestions: ... | ”...we share with the Cochrane Collaboration methodologists a reluctance to provide a risk of bias score that, by its nature, must make questionable assumptions about the relative extent of bias associated with individual items and fails to consider the context of the individual items.”...”...the risk of bias should be considered in the context of other limitations. If, for instance, reviewers find themselves in a close-call situation with respect to two quality issues.” | 72 |
| 37 | Hartling  (2^nd^ included publication by this author) | Strengths: …  Challenges: Risk of bias due to blinding varies depending on the targeted individuals  Suggestions: ... | “Researchers applying the risk of bias tool found it easier to assess blinding as three separate domains rather than a single item.”…”This study provides evidence that risk of bias due to blinding varies depending on the targeted individuals. Risk of bias due to 'other' sources is different from inappropriate influence of study sponsor.” | 53 |
| 38 | Hartling  (3^rd^ included publication by this author) | Strengths: The tool is a favourable approach  Challenges: Clear and consistent guidelines are needed  Suggestions: Funding as a separate domain | ”The risk of bias tool is a more favorable approach for assessing the internal validity of studies.”...”The factor that was most influential for risk of bias was the potential for inappropriate influence of study sponsors. We included this variable within the “other sources of bias” domain; however, users of the risk of bias tool may consider examining this variable separately due to the complex nature of funding source and its influence on the design, conduct, and reporting of trials.”…”Clear and consistent guidelines are needed for other users of the risk of bias tool.” | 93 |
| 39 | Hartling  (4^th^ included publication by this author) | Strengths: …  Challenges: “Small study bias” [is only included in the tools other bias domain]  Suggestions: ... | ”The final domain within the Cochrane tool includes an assortment of study characteristics that may lead to biased results, including factors associated with specific designs (eg, cross-over trials, cluster trials), blocked randomization in unblinded trials, and baseline imbalances.41,42 Sample size is not included in the tool; however, some evidence suggests “small study bias,” whereby trials with few participants may be associated with exaggerated effect estimates.” | 65 |
| 40 | Hartling  (5^th^ included publication by this author) | Strengths: The tool is appropriate for assessing internal validity  Challenges: Substantial tool agreement variation  Suggestions: Careful training and clear guidelines are required when applying the tool | ”...the risk of bias tool may be more appropriate for assessing a trial’s internal validity.”...”…the results provide some preliminary validation on the usefulness of the risk of bias tool to identify studies that may exaggerate treatment effects.”...”We found substantial variation in agreement across domains of the risk of bias tool.”...”There was low correlation between overall assessments using the risk of bias tool compared with [...] the Jadad scale and the Schulz approach.”...”Overall risk as assessed by the risk of bias tool differentiated effect estimates, with more conservative estimates for low risk studies. Careful training and clear guidelines are required when applying the tool.” | 101 |
| 41 | Hayden | Strengths: The tool does not use a summated risk of bias scores  Challenges: …  Suggestions: ... | ”In line with the Cochrane Risk of Bias tool for intervention studies (15) and the QUADAS-2 (Quality Assessment of Diagnostic Accuracy Studies) tool for diagnostic studies (16), we recommend against the use of a summated score for overall study quality.” | 40 |
| 42 | Hempel | Strengths: The tools strict criterias  Challenges: …  Suggestions: … | ”Where possible, criteria should be selected accordingly, particularly for critical appraisal instruments with very strict criteria, such as the Cochrane Risk of Bias tool [34,35], that routinely result in very imbalanced distributions - given that the number of studies with the rare expression of the moderator has pronounced implication for the statistical power and can only be compensated for statistically with a very large number of trials to ensure sufficient power.” | 71 |
| 43 | Higgins | Strengths: The tool is one of the most comprehensive tools  Challenges: The tool takes longer to complete than other tools The reliability of the tool has not been extensively studied  Suggestion: … | ”We believe our risk of bias tool is one of the most comprehensive approaches to assessing the potential for bias in randomized trials included in systematic reviews or meta-analyses.”...”The tool takes longer to complete than other tools.”...”The reliability of the tool has not been extensively studied.” | 46 |
| 44 | Hopewell | Strengths: The majority of systematic reviews uses the tool  Challenges: …  Suggestions: ... | “The majority (n=86/105; 82 %) of Cochrane reviews reported using the Cochrane risk of bias tool; five reported using more than one tool.”...”… Cochrane reviews are more likely to assess individual methodological components.”...”…it is unclear to what extent such restrictions should include all methodological components at high risk of bias…” | 49 |
| 45 | Ijaz | Strengths: The tool might be applicable for all study designs  Challenges: …  Suggestions: … | ”The idea of using the Cochrane risk of bias tool for all RCTs, CCTs, quasi-randomized trials, CBA studies, cohort studies, and all studies with a concurrent control group and a prospective follow-up seems logical [19]. The tool allows addition of new relevant domains of bias such as adjustment for confounding and except for the selection bias domain, which would probably be at high risk for all studies except RCTs with adequate allocation concealment, the rest applies in the same way to all studies of a prospective controlled design.” | 88 |
| 46 | Jo | Strengths: The tool is validated  Challenges: …  Suggestions: … | ”Validated quality assessment tools for RCTs include the Jadad scale, the van Tulder scale, The Cochrane tool, Newell's scale, the Scottish Intercollegiate Guidelines Network, and the National Institute for Health and Clinical Excellence.”...”...we [...] found by using the van Tulder scale and the tool that the quality was significantly higher for articles that had received funding.” | 56 |
| 47 | Johnson | Strengths: …  Challenges: Many best-evidence meta-analyses and those following Cochrane conventions have very small samples because they use methodological deficits as exclusion criteria  Suggestions: … | “…the Cochrane handbook recommends a risk of bias assessment that overlaps considerably with the Downs and Black (1998) and the Newcastle–Ottawa (Wells et al., 2000) inventories in its emphases on individual biases.”...”those using risk of bias nearly always left the items unscaled, consistent with Cochrane advice. None used both risk of bias and another instrument.”...”Furthermore, many best-evidence meta-analyses and those following Cochrane conventions have very small samples because they use methodological deficits as exclusion criteria.” | 76 |
| 48 | Kirkham | Strengths: The tool raizes awareness of outcome reporting bias  Challenges: …  Suggestions: … | ”Review authors will need to use their judgment regarding the potential for outcome reporting bias.”…”Adoption of the new Cochrane risk of bias tool, 11 which includes a judgment of the risk of selective outcome reporting, should also help to raize awareness of outcome reporting bias.”…”…consider the potential for bias caused by unpublished studies.” | 52 |
| 49 | Kirkham  (2^nd^ included publication by this author) | Strengths: The adoption of the tool helps to raize awareness of outcome reporting bias  Challenges: …  Suggestion: … | “The adoption of the new Cochrane risk of bias tool, which includes a judgment of the risk of selective outcome reporting for included studies, should also help to raize awareness of outcome reporting bias. By looking at only Cochrane systematic reviews, we suspect that our study underestimates bias due to changes in outcome specification during the systematic process. Cochrane reviews are not only monitored by a CRG but also the Cochrane Handbook provides guidelines which offer some protection against this type of bias [4].” | 84 |
| 50 | Lee | Strengths: …  Challenges: The van Tulder scale is more comprehensive than the tool because it includes 11 elements  Suggestion: Assess trials with multiple risk of bias scales | ”The van Tulder scale is more comprehensive because it includes 11 elements; the addition of the tool, which adds an assessment of the risk of study bias, increases the reliability of the results through the use of three complementary assessment tools. Therefore, the results of the evaluation by these methods can differ from each other. However, this study did not show the significant increase in the scores assessed by all of the assessment methods as well as the number of high-quality RCTs.” | 82 |
| 51 | Liu | Strengths: …  Challenges: In cases where a Cochrane risk of bias tool is used, reporting is sometimes incomplete  Suggestions: ... | ”In order to improve assessment of risk of bias, we recommend that the most recent version of the Cochrane risk of bias tool be used by SR and MA authors. Reviewers should continue to update their knowledge according to the latest Cochrane Collaboration Handbook versions and other developing methodology and to clearly state which version of the or handbook was used in their reviews.”...”In reviews published after 2008 in Chinese journals, Cochrane risk of bias tools were not always used. In cases where a Cochrane risk of bias tool was used, reporting was sometimes incomplete.” | 95 |
| 52 | Lundh | Strengths: …  Challenges: The current Cochrane Handbook recommendations are not clear about the usage of risk-of-bias assessments  Suggestions The tool revision should improve recommendations for assessing attrition bias and the usage of the risk-of-bias assessments | ”The Cochrane Handbook is currently being updated to ensure a more homogenous methodology in its reviews [43]. This revision is based on the acknowledgement of the discrepancies in assessment of methodological quality between the review groups [44], and it will involve introduction of a detailed risk-of-bias tool to be used in all reviews. The tool will also address bias in selective outcome reporting [45,46]. Finally, we suggest that the revision should improve recommendations for assessing attrition bias and the usage of the risk-of-bias assessments, as the current recommendations are not clear about this.” | 93 |
| 53 | Lundh  (2^nd^ included publication by this author) | Strengths: …  Challenges: The tool is unable to catch bias introduced by funding   Suggestions: Further methodological research should focus on how industry bias is handled | ”...our data suggest that industry sponsorship should be treated as bias-inducing and industry bias should be treated as a separate domain.”...”Interestingly, the AMSTAR tool for methodological quality assessment of systematic reviews includes funding and conflicts of interest as a domain (Shea 2007). Methods for reporting, assessing and handling industry bias and other biases in future systematic reviews must be developed. Specifically, further methodological research should focus on how industry bias is handled in Cochrane reviews.” | 75 |
| 54 | Manchikanti | Strengths: Once the learning curve has passed, reviewers will not only become comfortable but will start appreciating various insights of the tool  Challenges: …  Suggestions: ... | ”…once the learning curve has passed, reviewers will not only become comfortable but will start appreciating various insights of this instrument [the tool].” | 23 |
| 55 | O’Connor | Strengths: …  Challenges: The risk of bias might differ between different outcomes  Suggestions: Risk of bias should be judged for every outcome [proposedly with the tool] | ”Risk of bias should be judged for every outcome reported and the judgment reached may differ among different outcomes within the same experiment. Assessment of the risk of detection bias for a particular outcome often requires content expertize.”...”To assess internal validity, for each of the risk domains, we would propose following the approach proposed by the Cochrane Collaboration (Higgins et al. 2011) [ie. using the tool].” | 66 |
| 56 | Oremus | Strengths: …  Challenges: …  Suggestions: The tool reliability should be assessed in raters with different experience levels | ”While the Cochrane Collaboration has stated that quality scales and scale scores are inappropriate means of ascertaining study quality, 33 our results are relevant because many researchers continue to use the Jadad Scale and NOS in their systematic reviews. Indeed, our work suggests an area of future research. The Cochrane Collaboration has proposed a ‘risk of bias’ tool to assess the quality of RCTs.33 The reliability of the risk of bias tool should be assessed in raters with different levels of experience.” | 81 |
| 57 | Robertson | Strengths: The tool proved useful for demonstrating conservative effect estimates  Challenges: …  Suggestion: More tool levels and further validation could improve inter-rater agreement | “We achieved fair agreement between reviewers for risk of bias assessment of non-randomized studies. Although the process was time consuming, using a modified version of the Cochrane (risk of bias) tool proved useful for demonstrating conservative effect estimates in our systematic review. We suggest including more risk of bias levels and further validation could improve inter-rater agreement.” | 57 |
| 58 | Robinson | Strengths: Guidance is available on assessing the risk of bias of individual studies with the tool  Challenges: Little guidance addresses how to incorporate risk of bias information from an existing Cochrane review into a new Cochrane review  Suggestions: … | ”Guidance is available on assessing the risk of bias of individual studies, though little guidance specifically adldresses how to incorporate this information from existing reviews into a new systematic review [with the tool].” | 33 |
| 59 | Roseman | Strengths: The tool includes an optional “other bias” domain, which could be used to include conflicts of interests information  Challenges: …  Suggestions: Funding and COI [conflicts of interest] should be assessed as risk of bias | ”Currently, the Cochrane Collaboration's Risk of Bias tool includes an optional “other sources of bias” domain, 63 which meta-analysts could use to include information on COIs [conflicts of interests]. We recommend that the Cochrane Collaboration consider formalizing the requirement to assess potential bias from COIs [conflicts of interest].” | 48 |
| 60 | Sargeant | Strengths: The tool can be adapted to veterinary medicine [The tool] is a useful resource for reviewers  Challenges: …  Suggestions: … | ”The Cochrane ‘Risk of Bias'; tool could be easily adapted for use in veterinary medicine.”...”A useful validation and reviewer training strategy are to have all of the reviewers conduct risk of bias on a selection of articles that pass relevance screening. Poor agreement between assessors may lead to revisions or rewording of the risk of bias questions or may lead to additional training of reviewers in critical appraisal of the literature. There is an entire section of the Cochrane Review Handbook devoted to assessing the risk of bias (Higgins and Green, 2011), which is a useful resource for reviewers.” | 99 |
| 61 | Savović  (2^nd^ included publication by this author) | Strengths: Most authors likes the risk of bias standardized approach and find the workload acceptable  Challenges: Some find the wording describing judgments of risk of bias to be unclear  Suggestions: … | “Most respondents thought that risk of bias assessments were better than past approaches to trial quality assessment. Most authors liked the standardized approach (81%) and the ability to provide quotes to support judgments (74%). About a third of participants did not like the increased workload, and found the wording describing judgments of risk of bias to be unclear. Most authors (75%) thought availability of training materials was sufficient...”…”…respondents identified positive experiences and perceptions of the risk of bias tool. Revisions of the tool and associated guidance, and improved provision of training, may improve implementation.” | 94 |
| 62 | Shamliyan | Strengths: …  Challenges: …  Suggestions: Future tools should evaluate quality at both the study and hypothesis levels Future tools should grade the quality of a study as it applies to various subgroups when applicable | “Future tools [ie. the tool] should evaluate quality at both the study and hypothesis levels. One study can examine more than one risk factor with different degree of bias [86], [87] and [88] examined estimates in racial, ethnic, or other subgroups can have a greater degree of bias compared with the total study estimates [89], [90], [91], [92], [93] and [94].”...”Future appraisal tools should grade the quality of a study as it applies to various subgroups when applicable. We concluded that numerical scores are meaningless when examining the quality of studies in systematic reviews [95].” | 95 |
| 63 | Shrier | Strengths: …  Challenges: Risk of Bias tool may lead to double-counting of bias, and inappropriate inferences  Suggestions: … | ”Risk of Bias tool may lead to double-counting of bias, and inappropriate inferences.”...”…[the] current Risk of Bias tool [is] appropriate for observational studies with slight modifications.” | 25 |
| 64 | Sinha | Strengths: The tool has changed with blinding of participants and personnel assessed separately from blinding of outcome assessment adding clarity for reviewers  Challenges: Many “unclear” risk trials  Suggestions: … | “Many studies had an unclear risk of bias.”...”The tool has changed over time, with biases related to blinding of participants and personnel assessed separately from blinding of outcome assessment in the current tool, 5 adding clarity for reviewers.” | 37 |
| 65 | Turner | Strengths: …  Challenges: Consideration should be given to the type of outcome (i.e. objective or subjective outcome) when assessing bias  Suggestions: Further studies are needed to explore the role and effect of small study effect, reporting bias, higher risk of bias in single centre studies, or factors related to the selection of the participants, treatment administration and care providers’ expertize | ”...the Cochrane risk of bias tool suggests blinding of participants and personnel, and blinding of outcome assessment be assessed separately. Moreover consideration should be given to the type of outcome (i.e. objective or subjective outcome) when assessing bias…”...”...there has been some criticism of the Cochrane risk of bias tool [109] concerning its ease of use and reliability [110,111]...”…”…[Further studies are needed to explore the role and effect of] small study effect, reporting bias, higher risk of bias in single centre studies, or factors related to the selection of the participants, treatment administration and care providers’ expertize.” | 96 |
| 66 | Voss | Strengths: The tool represents an accepted standard for systematic reviews  Challenges: …  Suggestions: It would be informative to explore how different ways of deriving a summary score might influence results Need to develop and test a gold standard tool that is applicable across a broad range of epidemiological study designs and addresses all major aspects of internal and external validity | “...the Cochrane Collaboration Risk of Bias tool represents an accepted standard for systematic reviews.”...”It would be informative to explore how different ways of deriving a summary score and more or less rigorous cut-off values might influence the results.”...”Our study emphasizes the importance of quality appraisal in systematic reviews, and the need to develop and test a gold standard tool that is applicable across a broad range of epidemiological study designs and addresses all major aspects of internal and external validity.” | 80 |
| 67 | Wells | Strengths: …  Challenges: …  Suggestions: The next major step is to extend the existing Cochrane Risk of Bias tool so that it can assess the risk of bias to non randomized trials | ”The next major step is to extend the existing Cochrane Risk of Bias tool so that it can assess the risk of bias to NRS included in a review. This initiative involves detailed consideration of how the assessment of each bias domain may need to be customized to study designs.”...”Fundamental research into a number of particular topics for systematic reviews of NRS is critical. Topics such as the assessment of publication bias, and the need and justification for comprehensive searching require empirical evaluation. Empirical research is currently underway on selective analysis reporting related to RCTs.” | 95 |
| 68 | Zeng | Strengths: The tool is widely accepted and recommended  Challenges: …  Suggestion: … | ”For primary research, the Collaboration’s recommended tool for assessing risk of bias of RCT is neither a scale nor a checklist; it is a domain-based evaluation, in which critical assessments are made separately for different domains (5,12). The “Cochrane Collaboration’s tool” is very widely accepted and recommended. Additionally, the Modified Jadad Scale and PEDro Scale are also suitable tools for RCT.” | 61 |

Table III: Peripheral remarks on the Cochrane risk of bias tool for randomized clinical trials

| **Reference** | **First author** | **Selected commentaries from references** |
| --- | --- | --- |
| 69 | Bala | ”We conclude that RCTs published in higher impact journals were, on average, less prone to risk of bias compared with those published in lower impact journals in their reported design, conduct, and analysis. There remains a substantial room for improvement among RCTs published in all Core Clinical Journals. Journal editors and reviewers in higher and lower impact journals should insist on comprehensive explicit reporting of methodological (concealment of allocation, blinding, and loss to follow-up) and statistical issues (effect estimates and confidence intervals), including reporting of subgroup analyses (prespecified subgroup analyses and test of interaction). Users of the medical literature should critically appraize each trial regardless of the reputation of the publishing journal.” |
| 70 | Balk | ”We conclude that more research is necessary to better understand the utility of this new translation tool to reduce the risk of language bias in systematic review. However, in the meantime, it may be worthwhile for EPCs to devote the small amount of resources and effort necessary to try Google Translate to include non-English articles. It will be important, however, to recognize that extraction of these articles is more prone to error than extraction of typical English language articles.” |
| 71 | Bassler | ”In our prior work, we repeatedly referred to the bias associated with stopping RCTs early for efficacy. We now see this as a misleading characterization. As we will describe below, the overestimates that occur in trials stopped early for benefit are largely the result of random error. There is a bias, in addition, a bias that stopping early creates, but it is a relatively minor contributor to the overestimation. There is also potential for bias at the level of the meta-analysis, but this is due to factors other than bias at the level of the individual study.”...”In our view, the danger of overestimation will be particularly high when all the following three conditions exist: tRCTs have a relatively small number of events (e.g. <200), there is a substantial difference (e.g. a ratio of RR < 0.7) in the RRs between the tRCTs and the nontruncated RCTs; the tRCTs have a substantial (>20%) weight in the meta-analysis. When these conditions exist, systematic reviews should offer sensitivity analyses with and without inclusion of tRCTs. When the three conditions do not exist, and RCTs have instituted safeguards against bias, and yield precize estimates that are consistent across trials,25,26 authors of systematic reviews and meta-analyses can, regardless of the presence of tRCTs, have a high level of confidence in the results.” |
| 72 | Berger | ”Zambon et al. [1] appraized the quality of a series of meta-analyses, and found that “Internal validity appeared largely robust, as most (50.5 %) reviews were at low risk for bias.” To conclude that there is a low risk of bias, a comprehensive review is required, so that all potential biases are considered. Otherwize, we might as well notice that some raindrops have missed us as we run through the rain, and conclude, on that basis, that therefore we must be dry. This wishful thinking provides a false sense of security that interferes with required reforms, and is potentially quite harmful.”...”It is rather unnerving, given that randomized trials are the worst possible design except for all the rest [2], that fully half of the reviews could not meet even these minimal requirements, but what about the ones that did? We are told nothing about how well or poorly the trials were randomized, or even if they truly were randomized at all. The risk of bias depends critically on the precize methods of randomization [3], and not every trial that is labeled as randomized actually is [4]. Nor are we told how successful the masking effort was; the risk of bias is clearly high if masking is unsuccessful, and the effort should never be confused with completion of the mission (Section 1.8 of [3]). Beyond that, nothing is mentioned of the myriad numbers of other potential biases, including improper enrichment, improper surrogate endpoints, changing endpoints, post-randomization exclusions, and analyses whose validity is predicated on untenable assumptions.”...”Half of the meta-analyses should be dismissed out of hand, because they were unable to meet even the barest minimal requirements of validity. The other half are at a rather high risk of bias if we know nothing more about them than that they used the words “randomized” and “masked”, without qualification. And, unfortunately, the risk of bias in future trials and systematic reviews can only be expected to increase if we see more articles that turn a blind eye and contort themselves to somehow find something to praize. Valid trials need to address and rule out all the aforementioned potential biases, and valid appraisals of trial quality and internal validity need to do the same [5].” |
| 73 | Berger  (2^nd^ peripheral remark by this author) | ”Foley et al. [1] lamented the fact that allocation concealment is described inadequately in two-thirds of the studies”...”Foley et al. [1] also pointed out that allocation concealment continues to be misunderstood by many investigators”...”When the methodology is flawed, it sets a dangerous precedent. Hence, the record needs to be set straight, even when the right conclusions are found. In this case, the issue is what it takes to establish the concealment of future allocations. Is it enough to follow the Schulz and Grimes [3] definition?”...”here are two threats, including both (1) direct observation of the allocation sequence and (2) prediction of future allocations based on knowledge of the past ones [4] ([5]; Sections 2.5–2.6).”...”In fact one-third of the trials met only an appalling low standard of pseudo allocation concealment, but it is highly doubtful that even 10% reported and/or operated with true allocation concealment. Of course, we have no way to know for sure, because Foley et al. [1] did not enumerate the trials considered and the description of allocation concealment in each. What we do know for sure is that allocation concealment remains misunderstood even by those who would try to explain it to others. We know that the relevant literature continues to be ignored when it is convenient for authors to do so. And we know that the vast majority of trials cannot meet even so low a standard as the wink and the nod required, along with keeping a straight face, when reporting that the trial had or did or was blessed by allocation concealment. We know that in the future we all need to do better. Patients are depending on us to do so.” |
| 74 | Bialy | ”None of the studies had an overall low risk of bias. Most studies had a low risk of bias for the domain of incomplete outcome data (89%), while 63%, 55% and 46% of trials had low risk of bias for sequence generation, other sources of bias, and blinding of outcome assessors, respectively.”...”We examined risk of bias among a sample of 208 trials included in 24 neonatal systematic reviews. The majority of trials had an overall high risk of bias; none had an overall low risk of bias. This is consistent with other studies that have shown few trials to have low risk of bias overall (12,16,20–22). ”...”Selective reporting of outcomes was a major contributing factor to the number of trials assessed overall as high risk. Further, trials that were at high or unclear risk of bias for selective outcome reporting were associated with an overestimate of treatment effects by 47%.”...”For the majority of other domains, we did not find an association between risk of bias and effect estimates. This may be explained by the fact that most of the outcomes in our analysis were objective (i.e. 15/25 reviews included mortality in their primary outcome, Table 4). A recent study by Savovic et al. that combined seven meta-epidemiological studies involving 234 meta-analyses and 1973 trials found an exaggeration of treatment effects among studies with inadequate or unclear random-sequence generation, allocation concealment and blinding 32. However, they also showed that the bias was driven largely by trials reporting subjective outcomes with little influence by trials with objective and mortality outcomes. Another explanation for our findings may be related to insufficient power to detect differences; in particular, there were often few trials in the low risk of bias category.” |
| 75 | Chaimani | ”Based on the Cochrane Risk of Bias tool, 19 we sought information regarding the random sequence generation, allocation concealment, and blinding of patients and outcome assessors.” META-epidemiologic study. |
| 76 | Chase | ”While meta-analysis is a powerful tool to overcome the variation among studies and arrive at an answer to a particular scientific question (e.g., does a particular intervention alleviate the symptoms of a dizease?), it is less powerful in its ability to detect publication bias and the selective presentation of analyses. In the biomedical sciences, such biases not only slow the progression of science, but they could also result in bringing ineffective or harmful substances to clinical trial, creating considerable financial and health costs. Thus, it is important to understand just how rampant these biases are.”...”With increasing numbers of humans afflicted with neurological disorders, millions of animals sacrificed in the name of research, and billions of dollars spent on health care, it is imperative that biomedical scientists take action to alleviate these biases. Tsilidis and colleagues advocate a number of such actions, including the development of standard reporting protocols, preregistration of experimental design, and provisioning of raw data to the broader community, all of which should allow more efficient development of dizease interventions from animal models to clinical trials.” |
| 77 | Chess | ”It is important to note the difference between methodological quality and reporting quality. Our study is designed to evaluate the methodological conduct of studies; however poor reporting can innately make this task difficult. While it is imperative to decipher between reporting and methodology, it can be tempting to draw similar conclusions from both. This will ultimately hamper a true risk of bias assessment and must not be carried out.” |
| 78 | Chiappelli | ”Cochrane organization, now established world-wide and across all continents: the premier entity for generating and disseminating systematic reviews, and establishing the fundamental research synthesis methodology, including the risk of bias assessment tool.”...”Most of the scales available for this purpose are derived, or expanded from the original JADAD scale [9], and are often limited to rating subject randomization, blinding, and drop-out. These domains are hardly representative of the vast number of criteria that establish the standards of research methodology, design and data analysis. Other available instruments often suffer from fundamental flaws of reliability, including most importantly standardization of the readers as noted by Hartling and collaborators in a 2012 AHRQ report (“Inter-rater variability resulted more often from different interpretation of the tool rather than different information identified in the study reports…”) [10]. The report further states in no uncertain terms the “need to determine inter-rater reliability and validity in order to support the uptake and use of individual tools that are recommended by the systematic review community…” (p. 2). In brief, current trends demand an articulated research program for validating these tools, because of the fundamental importance and relevance of sound assessments of research quality to the process of obtaining the best available evidence...” |
| 79 | Christensen | ”There is empirical evidence that OA trials may be affected by selection and detection bias [11]. Allegedly, few patients noted the taste of fish oil during 12 weeks of taking such capsules three times per day. We argue that a fishy taste in the mouth might certainly cause detection bias. Assessment of the trial reporting in terms of risk of bias, the use of random assignment, and subsequent concealment of allocation would qualify as adequate (that is, low risk of selection bias); it seems reasonable that at baseline the patients in the study groups were similar with respect to prognostic factors. The reporting of double-blinding supports a low risk of performance bias as the authors state that the manufacturer provided both the Phytalgic® and placebo capsules and that it claimed that they were identical and indistinguishable. We argue, however, that it might be difficult to hide the taste of fish oil during a 3-month trial, probably as difficult as it is to hide the taste of ginger [5]. Finally, deviations from protocol and loss to follow-up often lead to the exclusion of patients after they have been allocated to treatment groups, and this may introduce attrition bias [12]. We are concerned about the fact that the trial registration was done after study completion (April 2008). Thus, we would categorize the risk of attrition bias as being at best unclear as there is a possibility that some patients were excluded from the analyses. Although the authors performed their analyses according to the intention-to-treat principle on what they claim is the correct sample size, we worry about the fact that the attrition rate was 10% (4/40) in the placebo group, whereas only 2% (1/41) withdrew from Phytalgic®.” |
| 80 | Chung | ”There are various types of qualitative assessment tools for RCTs including Campell, Moher, Chalmers, Jadad, van Tulder, Newell's, and Cochrane. The interesting point in this study is that by using three different tools, we found differences in the qualitative analysis outcomes of RCTs. The assessment of the quality of trials remains controversial, and there is no consensus on highly accurate and valid tools (17). However, in this study, efforts were made to overcome such limitations by using three different tools: the Jadad scale, van Tulder scale, and the tool. These are representative assessment tools used most commonly nationwide and worldwide. In particular, the Jadad scale has advantages in the simplicity of the assessment questions and ease of assessment performance, but it does not include assessment items for the most important item of RCT assessment: concealment of allocation. Therefore, additional analyses were performed using the van Tulder scale and the tool to supplement in this regard.” |
| 81 | Chung  (2^nd^ peripheral remark by this author) | ”Some generic quality issues are applicable to all SRs. For example, a comprehensive and transparent search strategy, with adequate justifications for inclusion or exclusion of specific studies, is needed to ensure an unbiased selection of studies for SRs and to improve understanding of how the SR was conducted. Furthermore, searching for unpublished data and comparing them with published data could shed some insights on the potential impact of publication bias (175). There is an underlying suspicion of publication bias against studies having either null or negative outcomes (176). It is important to note that there are no reliable methods to measure publication bias. Studies have shown that the most frequently used method to assess publication bias (funnel plots) can be misleading (177–179). Quality assessment of the primary studies is essential for the evaluation of validity and the overall strength of the conclusions in an SR.”...”Without quality assessments, the validity of the included primary studies is unclear and the impact of the potential biases in the primary studies on the conclusions of an SR cannot be assessed.” |
| 82 | Da Costa | ”The Cochrane Collaboration recommends a component approach to the assessment of trial quality and risk of bias, rather than the use of quality scales such as PEDro [12]. It was wondered whether the approach chosen might affect the conclusions of Cochrane reviews. To examine this, the review of transcutaneous electrostimulation for osteoarthritis of the knee was revisited [13]. The PEDro scores were available for 15 of the 16 trials included in the meta-analysis, with scores ranging from three to eight. These 15 trials contributed 17 comparisons. The Cochrane review had identified only one trial of clearly high quality [14], with adequate generation of random sequence, concealment of allocation, and blinding of study participants and therapists. This trial received a high PEDro score of eight; however, other trials that were also highly scored by PEDro lacked adequate concealment of allocation and blinding of patients or therapists [[15], [16], [17], [18], [19]]. A high PEDro score does thus not mean that the trial was adequately randomized and blinded.”...”The Cochrane meta-analysis was repeated [13] using the scores from the PEDro database to identify high-quality trials. Fig. 1 shows the summary effect sizes for pain for all trials, for trials of higher quality using different PEDro thresholds, and for the high-quality trial identified in the original review [13]. With the PEDro scale, the beneficial effect of electrostimulation became more prominent as the quality of the trials increased. In contrast, the estimate from the high-quality trial was close to zero, indicating little benefit of electrostimulation. The previous review concluded that the evidence was “inconclusive, hampered by the inclusion of only small trials of questionable quality” [13]. It seems likely that based on the results from the PEDro scale, many reviewers would conclude with the contrary opinion that there was robust evidence from high-quality trials that electrostimulation had a clinically relevant, beneficial effect on pain in osteoarthritis of the knee.”...”Greenland [20] described quality scoring as “perhaps the most insidious form of subjectivity masquerading as objectivity in meta-analysis”: the effects of quality dimensions that are important in a given study and context are diluted or confounded by the summary quality score, sometimes to the point that quality effects are no longer evident, or that effects are reversed, as in our example. The PEDro scale and many other quality scales include items that are not in fact related to the methodology and extent bias was avoided in a trial, but to the quality of reporting. Furthermore, items that are important for some interventions or outcomes may not be relevant in other situations but will receive the same consideration. For example, blinding of study participants will be crucial for pain but irrelevant for all-cause mortality.”...”the PEDro database's inappropriate emphasis on the use of summary scores from a quality scale makes the database less useful than it might be. It is likely that bias is introduced into systematic reviews and meta-analyses when these scores are used as the main criteria on which the inclusion or exclusion of trials is based. We suggest that the use of summary scores should be discouraged, and that the PEDro database be restricted to presenting the scores for individual items of the scale. The addition of items, for example on the prevention of differential expertize bias, and the removal of items not related to the risk of bias, might further enhance the value of this important initiative.” |
| 83 | Davey | ”It is clear that the numbers of studies eligible for meta-analyses are typically very small for all medical areas, outcomes and interventions covered by Cochrane reviews. This highlights the particular importance of suitable methods for the meta-analysis of small data sets.” |
| 84 | Dechartres | ”Results were robust after adjustment for RCT sample size, risk of bias as assessed by the risk of bias tool, variance of the log OR with consideration of publication bias, and funding source.”...”Sensitivity analyses adjusted for the domains of the risk of bias tool showed consistent results, with single-center status still significantly associated with larger treatment effect size.” |
| 85 | Doi | ”Da Costa et al. [1] suggest that the scoring of trials using quality scales be discontinued and try to provide evidence against such use by citing an example of a scale widely used in trials of physiotherapy. They repeated a Cochrane meta-analysis on electrostimulation for pain [2] using the scores from the PEDro database to identify higher vs. lower quality trials and pooled summary effect sizes for pain for all trials, trials of higher quality using different PEDro thresholds, and trials with adequate randomization, allocation concealment, and blinding. They note that with the PEDro scale, the beneficial effect of electrostimulation became more prominent as the quality of the trials increased. In contrast, the estimate from a single study that had adequate randomization, allocation concealment, and blinding (Garland 2007 in Fig. 1) was close to zero, indicating little benefit of electrostimulation. The implication they derived from this seemingly contradictory result was that quality effects were reversed, and although some of the blame was attributed to the characteristics of the PEDro scale, a large amount of criticism was leveled at the use of summary scores[1].”...”In reality, stratification of studies by quality score also stratifies them by precision and effect sizes. Thus, intermediate quality studies are likely to fall on different sides of the quality threshold with the use of different thresholds. If these studies happen to have a larger effect size or higher precision, this will markedly affect the magnitude and direction of the pooled effect under different quality thresholds depending on which side of the threshold a cutoff assigns such studies.”...”This sort of meta-analysis is thus amenable to serious bias from redistribution of precision and effect sizes depending on how the intermediate quality studies get assigned.”...”The subsequent variation in precision and effect sizes in the high- and low-quality groups was what led to discrepancies, and in the latter study too, there was effectively no quality effect assessed [6]. We conclude therefore that what da Costa et al. [1] report is not PEDro's bias but rather a bias in their interpretation of effects.” |
| 86 | Dossing | ”The Cochrane Collaboration's Handbook for Systematic Reviews of Interventions provides guidance to authors to critically review trial outcome using the risk of bias (risk of bias) assessment tool.5 The risk of bias tool requires authors to evaluate the well-established strengths and limitations of RCTs, including sequence generation, allocation concealment, blinding of participants, personnel and outcome assessors, loss to follow-up with failure to apply the intention-to-treat (ITT) principle and selective outcome reporting.5 7 In the course of meta-epidemiological studies, other sources of bias in RCTs have been identified, such as significant discrepancies favouring intervention in single (rather than multicentre) trials, in trials with small (rather than large) sample sizes and in using subjective (rather than objective) outcome measures.3 8–11 Most recently, funding source has become a distinct possibility as a source of bias, with for-profit organisation funding likely favouring prointervention results,12–14 however there is an on-going debate as to whether funding should be regarded as a risk of bias item.15 16”...”This study may point to potential bias and disadvantages in the handling of missing data in RCTs, otherwize known for having a low risk of bias compared with other study designs.62 SI has been criticized on a theoretical level, but its implication on efficacy outcomes in RA trials is uncharted. Accordingly, this study may provide empirical evidence that can support or contradict existing critics. Regardless of our findings one should always be careful when interpreting results from trials where data are missing and consider the reasons for missing data and potential impact on effect estimates.7 63” |
| 87 | Duclos | ”Other sources of reporting bias may include design-specific risks of bias, early stopping, baseline imbalance, blocking of experimental units in unblinded studies and differential diagnostic activity.”...”After carefully reviewing each study for potential bias, an overall assessment of the evidence for bias and the likely direction(s) and magnitude of the bias(es) should be made. If many of the studies that constitute the evidence base have a high likelihood of substantial bias, any conclusions should be treated with circumspection. Studies at high risk for bias may be excluded if the results are deemed unreliable.” |
| 88 | Duvendack | ”Bias assessment in quasi-experiments is complicated by the nature of the validity assumptions, for example unconfoundedness or exogeneity (Morgan and Winship 2007). There are a large number of existing tools to assess risk of bias, many of which enable integrated assessment of experimental and quasi-experimental designs 6 . The tools mostly rely on the assessment of group comparability in terms of observable covariates. Although some of the tools include vague questions about statistical validity, none provide further guidance to assess selection (and placement) bias and statistical analysis comprehensively. Operationalisation of existing tools to assess quasi-experimental designs used in development (including RDD, IV, PSM, DID) may therefore lead to simplistic and inappropriate study classifications. Adequate assessment of selection bias in quasi-experiments requires analysis of the methods of counterfactual identification to address selection bias (that is not just whether the study used random allocation), among other factors including file-drawer effects and the use of appropriate specification tests.” |
| 89 | Dwan | ”Hahn et al [13] compared the funder stated in protocol to publication. These studies indicate that funding is an important factor to consider when investigating publication bias and outcome reporting bias, however more work needs to be done to examine common questions before conclusions regarding the relationship between funding and outcome reporting bias can be drawn.”...”Another recommendation is to conduct empirical evaluations looking at both ORB and study publication bias in RCTs to investigate the relative importance of both i.e. which type of bias is the greater problem. The effects of factors such as funding, i.e. the influence of pharmaceutical industry trials versus non pharmaceutical trials, should also be factored in these empirical evaluations.” |
| 90 | Dwan  (2^nd^ peripheral remark by this author) | ”Systematic reviewers need to ensure they access all possible trial documentation, whether it is publicly available or obtained from the trialists, in order to assess the potential for selective reporting bias for analyses. The Cochrane risk of bias tool is currently being updated, and the revized version will acknowledge the possibility of selective analysis reporting in addition to selective outcome reporting. Selective analysis reporting generally leads to a reported result that may be biased, so sits more naturally alongside other aspects of bias assessment of trials, such as randomisation methods, use of blinding, and patient exclusions. Selective outcome reporting may lead either to bias in a reported result (e.g., if a particular measurement scale is selected from among several) or to non-availability of any data for a particular outcome (e.g., if no measures for an outcome are reported). The latter sits more naturally alongside consideration of “publication bias” (suppression of all information about a trial).” |
| 91 | Elkhadem | ”Quality of included studies was determined using Cochrane Collaboration risk of bias tool, while publication bias was not assessed.”...”The authors' recommendation should be considered with caution. First, three out of eight included studies performed adequate sequence generation, allocation concealment and blinding while the five remaining studies showed a high risk of bias. Nevertheless, all eight studies were included in the meta-analysis, which poses a threat on the validity of the final pooled estimate.” |
| 92 | Elkhadem  (2^nd^ peripheral remark by this author) | ”Quality of reporting trials was assessed using Cochrane Collaboration Risk of Bias tool. Trials with high risk of bias were excluded from meta-analysis. Four split mouth RCTs discussing bond failure risk were excluded due to inadequate or unclear randomisation procedures. These four trials included 1458 brackets in the SEP group and 1460 brackets in the AE group. No sensitivity analysis including trials with high risk of bias was performed; hence their effect on the direction of the results cannot be concluded.” |
| 93 | Engebretson | ”…studies were analysed separately according to risk of bias evaluation. Heterogeneity between studies was calculated in order to determine whether publication bias was significant.”...”For a meta-analysis to be high quality evidence, each of the trials included must also be of high quality, and that means each must have sufficient statistical power on its own.” |
| 94 | Eriksen | ”In addition, most of the positive findings were commercially funded ([16, 67]), which involves a risk of bias. To address this problem, we performed a sensitivity analysis restricted only to studies with a low risk of bias.” |
| 95 | Faggion | ”Most domains were judged to be at unclear risk of bias and therefore it is not possible to determine the degree of (un)biasedness of the described treatment effects. Note that risk of bias and quality of reporting should be considered distinct from each other.”...”Although direct contact with authors of the study might be an attempt for clarifying dubious or lack of information, this does not guarantee the accuracy of information provided (Haahr and Hróbjartsson 2006). We therefore adopted a conservative approach for assessing the domains; that is, we considered “unclear entries” as unclear risk of bias, although many of these domains would probably be scored at high risk of bias.” |
| 96 | Ferreira | ”Taking into account the set of 4,581 studies, only one study (0.22%) was classified as presenting low risk of bias for the four dimensions assessed and 77 potential RCTs (98.72 %) were classified as presenting high risk of bias.” |
| 97 | Fleming | ”As expected, the quality of Cochrane reviews was significantly better than non-Cochrane SRs. An area of particular concern in relation to non-Cochrane reviews was the failure to register reviews at the outset. Registration of Cochrane reviews is mandatory with publication of a protocol a priori. Use of a protocol pre-specifies the objectives and methodology reducing the risk of biased post hoc decisions.”…”In the present review, a problem with classification arose in respect of risk of bias assessment. Inclusion of a quality assessment is advocated in the AMSTAR guidelines as part of a comprehensive review process. However, QUORUM guidelines (Moher et al., 1999), which were advocated as a template for reporting prior to the advent of PRISMA (Liberati et al., 2009), recommended the use of methodological quality assessment rather than risk of bias assessment. Consequently, where assessment of methodological quality was undertaken, this was taken to be synonymous with risk of bias assessment in the present review. It should be noted, however, that risk of bias assessment is considered best reporting practice presently; this approach should therefore be used until the PRISMA guidance is superseded.” |
| 98 | Flodgren | ”CR has three additional domains in risk of bias-table: ”Similar baseline outcome measures”, ”Similar baseline characteristics” and ”Protection against contamination”. CR uses ”Other Bias” for: ”Only less than half of eligible hospitals agreed to participate which creates a greater risk of selection bias since the hospitals that declined to participate were different from the others (small and rural)” (High risk).” |
| 99 | Flores-Mir | ”…a point that I would like to raize is the inclusion of only RCTs. Due to the fact that the quality of the included RCTs was mainly poor, why not to include non-RCTs? Would they provide additional data that may be clinically useful, even considering the increased risk of bias among lower level of evidence studies? Sometimes a well-conducted non-randomized clinical trial can provide sound evidence in comparison to a poorly conducted RCT.” |
| 100 | Forbes | ”An attempt to blind participants and personnel does not always ensure successful blinding in practice. For example, for many blinded drug trials, the side effects of the drugs can reveal group allocation, unless the study compares two rather similar interventions (eg, drugs with similar side effects, or uses an active placebo.6 It has been suggested that it would be useful to ask trial participants at the end of the trial to guess which treatment they have received,7 ,8 and some reviews of such reports have been published.7 ,9 Evidence of correct guesses exceeding 50% would suggest that blinding may have been broken. However, responses may simply reflect the patients' experiences in the trial. A good outcome will tend to be more often attributed to an active treatment, and a poor outcome to a placebo.10 Risk of bias may be high for some outcomes and low for others. For example, knowledge of the assigned intervention may impact on behavioural outcomes (eg, number of visits to their physicians), while not impacting on physiological outcomes or mortality. Thus, assessments of risk of bias resulting from lack of blinding may need to be made separately for different outcomes. Rather than assessing risk of bias for each outcome separately, it is often convenient to group outcomes with similar risks of bias. For example, there may be a common assessment for all subjective outcomes (eg, quality of life) that is different from objective outcomes (eg, blood work).” |
| 101 | Foster | ”Bias — that is, the systematic distortion of the estimates due to poor design, conduct, or analysis of a trial5 — is particularly an issue for pragmatic trials of complex interventions where ‘real world’ estimates are paramount. Maximising generalisability and access to the key target group is important by careful attention to how the trial is communicated to potentially eligible patients, and recruitment methods that avoid reliance on busy clinicians (for example, using Read-Code activated electronic tags, or mailed invitation or population screening surveys of registered patients). Where the unit of randomisation is not the patient (cluster randomized trials) using such methods will also lessen selection bias, as clinicians in the control group may be less enthusiastic in recruiting patients, leading to differential selection bias.”...”The choice of control intervention is a difficult issue in trials of complex interventions. Trials with waiting list controls, usual care, or ongoing ‘stable’ medication provide intervention estimates which reflect the combined specific and non-specific effects that will accrue in practice, and are more likely to show between-group differences,6 but are less likely to motivate eligible patients to participate, and make attrition bias more likely. Conversely, using a ‘credible’ control or comparison intervention can help control for the known non-specific effects of complex interventions, ensure that the trial results are not simply explained by regression to the mean, natural history of the complaint or attention from a health professional, but provide an underestimate of the total effect of the intervention. More than one control group can be useful to tease out some of these issues,7 but equally it can be very difficult to separate fully the specific and non-specific effects of an intervention.8 Performance bias is a particular issue since an integral component of complex interventions is frequently patient–practitioner interaction or the ‘therapeutic relationship’. Complex interventions are rarely in standardized formats like a simple pill, rather most are individualized depending on presenting features, history, and response to the intervention. Factors such as the initial starting ‘dose’, the intensity of treatment progression, the frequency of sessions and contact time, degree of adherence required of the patient, and delivery as monotherapy or in parallel with other interventions may vary.” |
| 102 | Freemantle | ”To avoid bias, monitoring should be conducted in confidence and the results not communicated to study staff, investigators, or patients. Unfortunately, this does not always happen in device trials and investigator led trials. Further guidance is needed to bring all trials of human subjects up to adequate standards for safety monitoring.”...”ICH E9 provides unusually strongly worded guidance: “All staff involved in the conduct of the trial should remain blind to the results of such analyses, because of the possibility that their attitudes to the trial will be modified and cause changes in the characteristics of patients to be recruited or biases in treatment comparisons.2”...”Trials funded by commercial organisations are typically protected by confidentiality agreements, and clinical trials units may choose not to share their standard operating procedures, making it difficult to ascertain current practice in academic trials.” |
| 103 | Frosi | ”Many outcomes were not mentioned in trial reports meaning that clinical judgment was needed to decide whether the outcome of interest was likely to have been measured for a particular trial. A limitation of this study was that we did not contact trialists to determine whether outcomes were measured if they were not mentioned although any uncertainties in classifications were confirmed by contact with review authors. Our decision not to contact trialists in this study was a pragmatic one. The most recent trial published for inclusion in this study was published over 5 years ago (median publication date 1999), meaning that there would be obvious difficulties in locating most trialists. Nevertheless, the reliability of systematic reviews can be improved if more attention is paid to outcome data missing from the source trial reports. If data are missing, reviewers should be encouraged to at least attempt to contact the trialists or study sponsors to confirm whether the outcome was measured and analyzed and, if so, obtain the results and update the review meta-analysis accordingly with the newly obtained data. Reviewers should also be encouraged to complete the Cochrane risk of bias. A new version of the Cochrane risk of bias tool that includes a section on “bias in selection of reported result,” which is informed by the ORBIT study and is set to be launched in 2014 [21]. If obtaining outcome data is not feasible or successful then rather than do nothing, review authors are encouraged to apply a sensitivity analysis to assess the impact of ORB on an individual review.” |
| 104 | Garcia | ”…this review compared the results from the high quality trials to those from the low quality trials. Not surprisingly, the result from only the low quality trials indicated a protective effect against PTB as well as low birth weight from the periodontal treatment. They also noted publication bias that may have favoured reporting of positive trial results, particularly from smaller studies.” |
| 105 | Ghaeminia | ”The heterogeneity among studies was tested and the sensitivity analyses and Intention to Treat analyses were performed. A quality assessment according to the Cochrane Reviewers' Handbook was performed and the authors concluded that the included studies had unclear or high risk of bias and were therefore of poor to medium quality. However, the authors failed to describe possible confounding of their results due to the low quality of the included studies. If bias is present in some of the studies, meta-analysis will simply compound the errors, and produce a ‘false result that may be interpreted as having more credibility’. The authors failed to describe or discuss possible differences in patient characteristics at baseline of the included studies. For example, smoking and poor oral hygiene are significant risk factors for post-operative infection and alveolitis. None of the included studies reported these parameters, which may bias the results, especially if there was no randomisation. If, hypothetically, there were fewer smokers in the coronectomy group compared to the extraction group, the incidence of post-operative infection and alveolitis would be higher in the extraction group. Another important potential bias is the surgeon's skill and experience.”...”When the primary analysis is based on all studies, we would suggest incorporating the assessments of risk of bias into measures of the quality of evidence, for example using the GRADE system. This can help to ensure that judgments of risk of bias, as well as other factors affecting the quality of evidence, such as imprecision and publication bias, are appropriately taken into consideration when interpreting the results.” |
| 106 | Goodman | ”Metabias poses a real challenge for comparative effectiveness research and evidence-based health care because it is typically neither discernable nor explainable from an individual study report, but is manifest only when looking at collections of those studies. These studies are in turn used to create treatment recommendations and practice guidelines.”…” This has led some to explore whether industry sponsorship by itself should be considered a bias, or by our criteria, a metabias (11–13).”...”Without an independent gold standard for the truth, one cannot even say for sure which class of studies is biased...”...”To look askance at the findings of every single-center (or industry-funded) study may be a form of unfair “methodological profiling” because most such studies will be innocent of bias-inducing infractions. On the other hand, if we notice such an effect, can we ignore it? Understanding whether these findings are caused by a procedural problem in the conduct of randomized, controlled trials is critical to knowing how these studies should be handled in evidence syntheses and to formulating remedies. This finding, if confirmed, shows us that we have more work to do in understanding when we can rely on published medical evidence and what to do when we cannot.” |
| 107 | Greenhalgh | ”Box 1: Crisis in evidence based medicine? The evidence based “quality mark” has been misappropriated by vested interests. The volume of evidence, especially clinical guidelines, has become unmanageable. Statistically significant benefits may be marginal in clinical practice. Inflexible rules and technology driven prompts may produce care that is management driven rather than patient centred.”...”Evidence based guidelines often map poorly to complex multimorbidity.”...”To support real evidence based medicine, and in particular to reassure policy makers, clinicians, and the public that research and the guidance derived from it can be trusted, 63 the infrastructure for research and guideline development must show the highest standards of probity. Independent funding of national bodies for medical research is crucial.”...”In relation to producing usable evidence, we need to identify how to balance gold standard systematic reviews with pragmatic, rapid reviews that gain in timeliness and accessibility what they lose in depth and detail.65 In the same vein, we need research on how and in what circumstances to trade detail for brevity in developing guidelines. We need to develop decision aids that support clinicians and patients to clarify the goals of care, raize and answer questions about the quality and completeness of evidence, and understand and contextualize estimates of benefit and harm. We also need to improve both the usefulness and ease of use of these and other evidence based tools (models, scores, algorithms, and so on) including the intellectual, social, and temporal demands they make on users and the resource implications for the healthcare organisation and system.”...”in relation to the collective effort to prevent the misappropriation of the evidence based quality mark, a key research priority remains the study of hidden biases in sponsored research—for example, by refining the statistical techniques for challenging findings that appear too good to be true.”...”…evidence based medicine has not resolved the problems it set out to address (especially evidence biases and the hidden hand of vested interests), which have become subtler and harder to detect. Furthermore, contemporary healthcare’s complex economic, political, technological and commercial context has tended to steer the evidence based agenda towards populations, statistics, risk, and spurious certainty.” |
| 108 | Hamm | ”...there was more uncertainty surrounding identification of problems with allocation concealment, incomplete outcome data, and “other sources of bias” (see Additional file 2). Despite this range of awareness of issues relevant to bias, 94.2% of respondents felt confident in their ability to evaluate the quality of published trials.”...”While most survey respondents indicated that bias is a problem, the interview data suggested that trialists often do not have the knowledge to first, recognize, and second, address bias in their studies. They often mentioned a lack of formal training, instead relying on skills learned on the job.”...”Addressing specific biases, survey and interview respondents reported challenges with blinding most frequently, which included the cost of providing a placebo, difficulties in blinding non-pharmacological interventions, and blinding all relevant parties, including parents.”...”The major barriers to minimizing risk of bias in trials were related to awareness and environment. With little emphasis on research methodology in clinical curricula, many investigators are not adequately prepared to design trials with high levels of internal validity or to recognize and attend to issues as they arize.” |
| 109 | Hamm  (2^nd^ peripheral remark by this author) | ”There is a body of evidence suggesting that pediatric randomized controlled trials (RCTs) are susceptible to methodological limitations, and a substantial proportion of the studies conducted are at a high risk of bias [5]–[14], increasing the likelihood that treatment effects are being exaggerated. In two evaluations that assessed the overall risk of bias of pediatric RCTs using the Cochrane Collaboration’s Risk of Bias tool, more than 90% of studies were at high or unclear risk of bias, and these trials reported larger effect estimates than studies at low risk of bias [12], [14]. Guidance on rigorous trial conduct and reporting is available in abundance, demonstrating the negative impact of design elements such as improper sequence generation, allocation concealment, and blinding [15]–[25]; as is research on specific challenges inherent to trials in child health, for example, recruitment and consent [26]–[30]. However, a research-practice gap persists between what is known about bias and how RCTs are conducted, indicating a need for KT research in this population.” |
| 110 | Hansen | ”There remain additional possible contributors to bias which are not currently standard components for the Cochrane risk of Bias tool.” |
| 111 | Hartling | ”The difficulties in interpreting study design labels and the consequent difficulties in reaching agreement in assigning these labels to individual studies are consistent with those of other researchers. These issues have led some authors to direct systematic reviewers to focus on features of designs rather than on design labels when assessing studies for inclusion and evaluating potential risk of bias.3”...”The appropriate classification of studies by design is a critical step in a systematic review in order to guide inclusion, risk of bias assessments, pooling of studies for analysis, interpretation of results, and grading the body of evidence. We believe that a tool such as the one tested in this study would be useful to guide this process, although application of the requires several considerations in order to optimize agreement and reliability among reviewers.”...”Finally, in order to inform this field more broadly, work is needed to quantify the bias associated with design labels and the differences between studies that they help to identify.” |
| 112 | Hartling  (2^nd^ peripheral remark by this author) | ”...we would recommend that trials not be excluded from SRs and/or meta-analyses based on high or unclear risk of bias assessments. Rather, risk of bias should be explored as a potential source of heterogeneity where there is substantial variation observed in effect estimates across studies.”...”…we found that a high proportion of our sample of trials was at high or unclear risk of bias for many domains [...] only 3% were considered low risk of bias...”...”From an epidemiological perspective, there may be no difference in how typical biases (e.g., selection, performance, detection, attrition, reporting) operate in trials based on population characteristics.” |
| 113 | He | ”The quality assessment involved whether the randomization methods, allocation concealment and blinding were adequate or not based the study reported.”...”Many studies have showed that RCTs not using randomization, allocation concealment or blinding exaggerate estimates of effect to various extents. Compared with the RCTs using blinding, the RCTs not using blinding yield 17% larger estimates of treatment effects and in trials with subjective outcomes, effect estimates are exaggerated by 25%. Compared with the RCTs using adequate allocation concealment, RCTs using unclear or inadequate concealment of allocation exaggerate estimates of effect by 30%-41% [10-14]. These showed that compared with other ”flaws”, unclear or inadequate allocation concealment will cause a larger bias, which highlights the importance of allocation concealment. This study indicates that the adequate allocation concealment takes up the smallest proportion (7%) of the three assessed aspects. Although the adequate randomization methods accounted for a larger proportion (12%) than allocation concealment, there are also some investigations which showed that only 6.8% of the RCTs published in Chinese journals were deemed authentic randomized trials [19]. So the quality of the TCM RCTs in this study may be overstated.” |
| 114 | Herrera | ”The risk of bias was considered as medium to high. In addition, all studies had a limited sample size (from 10 to 33 patients).” |
| 115 | Hopp | ”Other bias refers to any other source of bias that might be specific to the particular study design, related to fraudulent data or other sources of bias.[2]” |
| 116 | Hróbjartsson | ”We encourage extensive pretrial testing of blinding procedures and explicit reporting of who was in the blinded condition and the methods used to ensure blinding.”...”To facilitate communication of blinding procedures in randomized clinical trials, the traditional terminology uses the phrases “single-blind,” “double-blind,” and sometimes “triple-blind.” These terms derive from the very early days of clinical trial development and have become deeply rooted. Unfortunately, they are ambiguous.”...”In general, traditional blinding terminology does not serve as a means of unambiguous scientific communication and should be abandoned, or at least complemented by an explicit reporting of who exactly was blinded.”...”Common phrases used to describe key trial persons can be ambiguous as well (Tabl e 1). A broad category such as “investigator” is imprecize. Confusion may also arize in trials in which the same physical person fulfills the role of health-care provider, outcome assessor, and data analyst; in trials that rely only on patient-reported outcomes; or in trials in which the same person both adjusts dosage and assesses outcomes. Furthermore, outcome adjudicators, i.e., those who decide whether a patient experienced an outcome of interest, are sometimes called “judicial outcome assessors” or “secondary assessors.” These terms may be easily confused with “outcome assessors,” that is, the persons who assess patients and provide outcome data, some-times called “data collectors” or “primary data collectors.” Given the variable terminologies in use, any description of blinding should be very precize with regard to whether and how all key trial persons were blinded.” |
| 117 | Innes | ”Unfortunately, there were only six studies that met the inclusion criteria and their quality was not high. The main limitations seem to be with data collection, both in the methods used and in what was collected/reported. There were no sample size calculations nor was population representative sampling carried out. In fact, most of the studies were carried out in dental schools, with ‘convenience sampling’. Many of the studies did not report exactly where the dental anomaly was, ie whether it was on the cleft side or non-cleft side, and this limits interpretation of the data. In addition, the authors had to impute some of the non-reported data, based on what was reported. One other methodological factor that has to be taken into consideration is that the authors used a controversial approach to the meta-analysis, weighting studies rated as being at moderate risk of bias more than those rated as being at high risk of bias.” FROM ORIGINAL ARTICLE: ”We performed a quality assessment of the remaining studies to control for influence bias, to gain insight into potential comparisons, and to guide interpretation of findings (Higgins and Green, 2005). Selected articles were assessed in accordance with the modified criteria of Loney et al. (1998). Seven criteria were analyzed, and a methodologic scoring system was used to rate the quality of the papers. The authors recommended weights for each item for the scoring system. Thus six criteria were assigned a score of 1 point. Only one was assigned a score of 3 points because it evaluated the distribution of dental anomalies according to the region or according to the arch segment/class of teeth, making 9 the maximum score possible. After this, researchers classified the studies into three categories with scores “A” to “C” according to predetermined criteria for method and performance. To obtain score “A,” low risk of bias, the study should present 8 to 9 points in the methodologic scoring system; to obtain score “B,” moderate risk of bias, the study should present 5 to 7 points; and to obtain score “C,” high risk of bias and poor quality, it should present 1 to 4 points. Studies assigned the higher scores (“A” and “B”) were weighed more heavily when the meta-analysis was performed.” |
| 118 | Jakobsen | “The bias risk domains generation of allocation sequence, allocation concealment, blinding of participants and treatment providers, blinding of outcome assessors, incomplete outcome data (see paragraph below), selective outcome reporting, and industry funding have been shown to be of particular importance [13, 58–64].” |
| 119 | Jo | ”…we found, using the van Tulder scale and the tool, that the number of high-quality articles was significantly higher for articles that had received funding. We also found a significant difference in RCT quality for blinding, which was noted using all three tools. This highlights the importance of using blinding to enhance the quality of an RCT.”...”The results presented here should be interpreted within the study limitations. First, no one representative assessment tool is available for the qualitative analysis of an RCT. Also, there is not one tool that can assess all of the items listed in the CONSORT statement. However, three representatively used tools for quality assessment of RCTs that covered the majority of items within the CONSORT statement were used to supplement this limitation. Second, because of the nature of the manual searching and evaluation used in this study, assessor bias may have influenced the selection and/or assessment process. This limitation was minimized through the use of two reviewers who independently extracted and assessed the RCTs, as well as the use of a third reviewer who moderated any discrepancies.” |
| 120 | Johnston | ”Studies focusing on PROs often use a number of instruments to measure the same, or similar constructs. This situation creates a risk of reporting bias. It is possible for investigators to measure a number of outcomes, and only report those that showed significant effects. Methodologists have long suspected the existence of outcome reporting bias [21,22], and systematic investigations comparing RCT protocols and their subsequent publications have provided estimates of its magnitude [23-25]. Investigators have examined a random sample of 156 completed Cochrane reviews that included 10 or more studies [26]. They found that a median of 46% of the review’s eligible trials (IQR: 20 to 75%, range: 2 to 100%) contributed to the pooled estimates. Thus, approximately half of the RCTs identified by the Cochrane reviews did not contribute to the pooled effect size in their meta-analyses. Furthermore, they found a correlation between effect size and the number of studies included (the fewer the studies, the larger the effect size) and this effect appeared strongest in studies using continuous outcomes (the correlation between the percentage of trials included in a meta-analysis and the SMD was -0.18 (95% CI: -0.35 to -0.01, p = 0.04). When analyses included less than 20% of eligible studies the mean effect size was 0.64 and when they included over 80% of the eligible studies the mean effect size was 0.31. These results demonstrate just how frequently studies fail to provide data for meta-analyses, and provide support for the existence of reporting bias in which investigators are inclined to selectively report results with larger effects. Systematic reviews focusing on PROs should be alert to this problem. When only a small number of eligible studies have reported a particular outcome, particularly if it is a salient outcome that one would expect conscientious investigators to measure, reviewers should note the possibility of reporting bias and consider rating down confidence in estimates of effect in their summary of findings table [27].” |
| 121 | Jørgensen | ”We found that meta-analyses with non-profit or no support are of better methodological quality on average than those with industry support. Lack of allocation concealment and blinding, and high attrition rates in randomized controlled trials may bias results of meta-analyses, but if the authors fail to describe these details, the reader is not able to judge if the meta-analysis is reliable. Most industry-supported meta-analyses failed on these counts; this agrees with results we have published previously [8].”...”Industry support was defined as authorship, provision of grants to authors of the meta-analysis, or other major assistance such as help with the statistical analysis. We did not consider provision of references or unpublished trial reports as support.”...”Our definition of industry support does not distinguish between different amounts of support, and our judgement of support is based on details reported in the meta-analyses. This can theoretically lead to misclassification of the support, as industry support may range from very little to generous, and details about some types of support may be lacking more often than others. However, the definition is operational and we believe that it includes the most important types of industry support. Lack of details or transparency in meta-analyses may also have led to misjudgement of the methodological quality, and it has been argued that the methodological superiority of Cochrane reviews can be explained by the fact that there are no word limits in the Cochrane Library. However, the methodological quality of Cochrane reviews published in regular journals do not seem to differ from Cochrane reviews published in The Cochrane Library [16,17]. Furthermore, important methodological details should always be made available in journals with a word limit, either in the article itself, or in material on the journal's website.” |
| 122 | Kalha | ”The flow diagram effectively summarizes the issue of evidence related to orthodontics with a screening of 1572 records, assessment of 117 full text articles for eligibility with 17 studies being eventually included in a quantitative analysis. The assessment of risk of bias is impressive and adds to the strength of the review.” |
| 123 | Kirkham | ”The reliability of systematic reviews can be improved if more attention is paid to outcome data missing from the source trial reports. If data are missing, reviewers should be encouraged to contact the trialists to confirm whether the outcome was measured and analysed and, if so, obtain the results. If this approach is not feasible or successful, as often is the case, then rather than do nothing, review authors are encouraged to apply a sensitivity analysis to assess the impact of outcome reporting bias on an individual review. If the results are not robust to outcome reporting bias, the review conclusions may need to be adjusted. The multivariate meta-analysis approach offers one such sensitivity analysis to adjust for outcome reporting bias when there is missing trial data for many review outcomes. Our recommendation to reviewers would be to use the multivariate meta-analysis approach if one is reasonably confident about the correlation estimates between outcomes (either from actual data, IPD from a single study or the Pearson estimate) or use an alternative univariate adjustment approach, for example the bound for maximum bias [6] if one is not confident about the correlations between outcomes. Where the multivariate approach is desirable but estimates of correlation are imprecize or clinically unexpected (as the negative correlation was in the beta-lactam example), one can consider clinical or biological reasoning to inform the correlation, or consider sensitivity analyses over a range of sensible values.” |
| 124 | Koretz | ”...none of the trials of early enteral nutrition qualified as low risk of bias. Thus, the trials were analyzed with three other approaches: 1) Meta-analyses of trials containing at least three adequate domains (‘more robust’) were compared to meta-analyses of trials with fewer domains being so graded (‘less robust’). 2) Trials were grouped by Jadad scores13 (e-Table 3) ≥3 and ≤2; meta-analyses were conducted for each group. 3) For each individual domain, meta-analyses of trials judged to be adequate were compared to meta-analyses of trials judged to be not adequate (inadequate or unclear).”...”In our review, the single domain that failed to follow the typical pattern of showing more favorable effects in trials with more bias was intention-to-treat.”...”We required 100% followup for a judgment of adequate intention-to-treat.”...”Our use of a point scale to assess the overall risk of bias assumes that each domain has an equivalent effect. This is probably not true and was the reason for us to look at each domain individually. However, when there are multiple risks of bias, any attempt to demonstrate an effect of bias from only one domain will be confounded by the other residual risks of bias diluting the effect of the single domain being considered, especially when there are few trials in total. This insensitivity in assessing individual domains may have been why systematic error appeared to be less influential on the results involving infectious morbidity.” |
| 125 | Kraglund | ”The designation of high risk of bias was awarded because of lack of blinding in one study, incomplete outcome data in two studies, and selective reporting of data in seven studies.”...”The authors also carried out subgroup analyses and sensitivity analysis to ensure robust conclusions by excluding studies of high or unclear risk of bias.” |
| 126 | Krauth | ”Although a sample size calculation is not a risk of bias criterion, it is an important characteristic to consider in evaluating an overall body of evidence.”...”Although some risk of bias criteria have been investigated primarily in human studies, they warrant consideration for animal studies. Reviews of clinical studies have shown that study funding sources and financial ties of investigators (including university- or industry-affiliated investigators) are associated with favorable research outcomes for the sponsors (Lundh et al. 2011). In that study, favorable research outcomes were defined as either increased effect sizes for drug efficacy studies, or decreased effect sizes for studies of drug harm. Selective reporting of outcomes and failure to publish entire studies is considered an important source of bias in clinical studies; however, little is known about the extent of this bias in animal research (Hart et al. 2012; Rising et al. 2008).”...”Further research should consider potential interactions between criteria for assessing risk of bias. Existing instruments have tested the association of study design criteria on effect size using univariate models. Multiple regression models should be used to ascertain the relationship between a study design criterion and effect size when taking into account other criteria in the model. Covariance between methodological criteria should also be examined. For example, randomized studies may be less likely to omit blinding than nonrandomized studies (van der Worp et al. 2010). Knowing the relative importance of these criteria will provide additional support for inclusion of specific criteria in risk of bias assessment instruments.”...”The most complex instrument had 25 criteria (Agerstrand et al. 2011).”...”As happened for clinical research, reporting of animal research is likely to improve if risk of bias assessments become more common.” |
| 127 | Krauth  (2^nd^ peripheral remark by this author) | ”…we recommended the use of empirically tested criteria and we pointed out criteria that have been shown to be a risk of bias. We caution against gathering judgments on how to assess study quality and propose that evidence should guide such evaluations. We propose an empirically based approach—as opposed to consensus-based opinion of experts—as this would provide a more unbiased evaluation of the data.” |
| 128 | Lambert | ”The risks of bias were difficult to evaluate. The overall quality of the studies was limited due to unclear risk of bias. Due to the small number of studies, it is not possible to assess publication bias. ” |
| 129 | Langford | ”We used the ‘other bias’ domain to note any additional concerns relating to study quality that did not fit into any of the previous five domains. For example, in this domain we included concerns about recruitment bias, baseline imbalances between groups, or selective reporting of subgroup analyses. We assessed the overall quality of the body of evidence for each outcome using the GRADE approach (Schünemann 2011). Using this method, randomized trial evidence can be downgraded from high to moderate, low or very low quality on the basis of five factors: limitations in design or implementation (often indicative of high risk of bias); indirectness of evidence; unexplained heterogeneity; imprecision of results; or high probability of publication bias.” |
| 130 | Li | ”The assessment of the risk of bias and its consideration in the network meta-analysis is far more challenging than in conventional meta-analysis. Risk of bias refers to the problems with the design and execution of individual trials that raize questions about the validity of their findings [6]. A fundamental difference between a conventional pair-wize meta-analysis and network meta-analysis is that a conventional pair-wize meta-analysis yields only one pooled effect estimate whereas a network meta-analysis yields more than one pooled effect estimate. Thus, while bias in the effect estimate from any single trial affects a single pooled effect estimate in a conventional meta-analysis, it may affect several pooled effect estimates obtained in a network meta-analysis. For example (Figure 1), the risk of bias for trials contributing to the direct comparison within a network may be low (e.g., all A vs. C trials described adequate masking), but the risk of bias for trials contributing to the indirect comparison may be high (e.g., some A vs. B or B vs. C trials reported no masking). In addition, the risk of bias may differ across different regions within the network of interventions being examined. Future methodological research should address ways to deal with such variation in risk of bias between direct and indirect comparisons and across the network. Specifically, such research may examine the impact of risk of bias in an individual trial on the network meta-analytic effect estimates, identify the biases specific to the network meta-analysis context that need to be considered, develop methods to assess, summarize and present the variation in risk of bias across the network, and use empirical research to postulate guidance for network meta-analysts on incorporating bias assessments in statistical analyses. Finally, methodological research may also examine whether network meta-analysis offers a potential method for identifying and adjusting for biases within included trials [10,15,23].”...”Factors such as the total number of trials in a network, number of trials with more than two comparison arms, heterogeneity (i.e., clinical, methodological, and statistical variability within direct and indirect comparisons), inconsistency (i.e., discrepancy between direct and indirect comparisons), and bias may influence effect estimates obtained from network meta-analyses. Heterogeneity, inconsistency, and bias may propagate through a network of trials, and may affect the estimates differentially across regions of the network. A range of methods to detect, quantify and deal with heterogeneity, inconsistency, and bias has been proposed [10-12,15,23].” |
| 131 | Lim | ”Abstract: tools for assessing methodological quality or risk of bias in randomized controlled trials (RCTs) and non-randomized studies (NRS) were reviewed. The van Tulder scale and Cochrane's assessment of risk of bias are the two most useful methodological quality evaluation tools for RCTs. Cochrane's tool includes sequence generation, allocation of sequence concealment, blinding, incomplete outcome data, selective outcome reporting, and other potential sources of bias. The Cochrane Collaboration Group recommends the Downs and Black instrument and the Newcastle-Ottawa Scale for evaluating the quality of NRS. In conclusion, this study offers useful information to physicians about tools for assessing the quality of evidence in clinical guidelines. Further research is needed to provide an essential core for evidence-based decision making regarding levels and/or grades of recommendations.” |
| 132 | Louis | ”…three of them had low risk of bias.24 ,25 ,35 When pooled, studies with high risk of bias showed a benefit of IVIg in reducing ET (3 studies, 110 neonates, RR 0.21, 95% CI 0.10 to 0.45, I2 = 0%), whereas studies with low risk of bias did not show statistically significant difference (3 studies, 190 neonates, RR 0.82, 95% CI 0.53 to 1.26, I2 = 0%)” |
| 133 | Lutomski | ”…we support that journal editorial guidelines and reviewers request data on missingness if they are not aptly discussed in an original article.”...”As highlighted by Sterne et al,3 when incorrectly performed or when underlying assumptions are not met, multiple imputation may in fact introduce bias into the analysis that can potentially lead to faulty conclusions.”...”Adapted from earlier work that demonstrated the utility of causal diagrams to theoretically identify confounding,4 Daniel et al5 have demonstrated the same theory can be applied to determine whether a complete case analysis or multiple imputation will produce biased estimates of association.”...”…we would like to emphasize that missing data analysis requires careful consideration to ensure the most accurate results. In this regard, causal diagrams can serve as a useful aid for choosing the preferable method for treating missing data.” |
| 134 | Ma | [Recommends the tool for especially ‘open’ randomized clinical trials.] |
| 135 | MacLennan | ”Background: In instances where randomized controlled trials (RCT) are impossible or have not been conducted, clinical recommendations and decision-making must rely on other evidence. If systematic reviewers decide to include non-randomized studies (NRS), it is imperative to use a standard method to assess and communicate the risk of bias (risk of bias) in NRS. Objectives: To pilot a risk of bias tool for NRS and make it commensurate with GRADE. Methods: An extended version of the Cochrane RCT risk of bias tool was applied to NRS. This included an additional item on the risk of findings of an NRS being explained by confounding. Each pre-specified confounding factor was assessed on the precision of measurement, baseline imbalance, and quality of case-mix adjustment, on 5-point scales. Imbalance was judged by clinical consensus, while other items were assessed by two independent reviewers. Mean 'adjustment' scores per outcome across studies were used to determine the quality of evidence according to GRADE. The tool was applied to 33 NRS retrieved for a systematic review of surgical interventions for localized renal cancer. Results: The initial 5-point scale was unwieldy and lead to disagreement among reviewers. We created scoring guidelines and re-piloted. Risk of bias scores were tabulated rather than aggregated to indicate where likely biases were located. All NRS were rated as either 'low' or 'very low' on GRADE; however, determining an appropriate cut-off required considerable judgement. Conclusions: Compared with risk of bias assessment in RCT, assessment of NRS was more difficult and increased required time and expertize resources. In areas where the quality of studies is known to be very low, the added time and complexity may make the assessment not worthwhile. Presentation of the large amount of information generated by this tool is challenging. Further research needs to strike a balance of making a 'brief' and 'easy' version while addressing complex methodological issues inherent in NRS.” |
| 136 | Matthews | ”The major flaw in this review is the application of an adaptation of the Newcastle-Ottawa Scale (NOS) to determine risk-of-bias for included trials (http://www.ohri.ca/programs/clinical_epidemiology/nosgen.pdf). Risk-of-bias assessment tools are used in systematic reviews in a number of ways - as a threshold for inclusion of studies; as a possible explanation for differences in results between subgroups of studies; by performing sensitivity analyses where only some of the studies are included; or by using a qualitative score as a weight in a meta-analysis of the results. However, empirical research does not support the use of these scales other than to describe the potential biases of each included trial. In fact, the Cochrane Collaboration, the group responsible for the majority of published systematic reviews in health literature, advizes against the use of scales.1” |
| 137 | McCay | ”By singling out industry ties, 1 the BMJ proposes to systematically discriminate against a particular group of people without evidence based justification. This is concerning not only from an ethical perspective but also because it ignores the wider issue of conflicts of interest, introduces bias, deters productive collaborations, marginalizes industry doctors rather than incentivising their academic and ethical rigour, and deprives readers of relevant, diverse healthcare perspectives. Few editorials or clinical reviews exist in a vacuum, entirely free of influence or motivation, and current conflict of interest procedures are insufficient to understand these influences or interpret their impact. Let us improve the scope, detail, and prominence of reporting and discussion of conflicts of interest using the principles of shared decision making to facilitate interpretation of how particular conflicts might affect a viewpoint. Thus readers will be exposed to wider perspectives and be able to make up their own minds, rather than have their access to the full healthcare picture restricted paternalistically.” |
| 138 | Millett | ”A key question that must be asked when looking at all Cochrane reviews is whether the review has been undertaken in a way to avoid bias and whether the data and results from the included studies are valid.10 Conclusions may be distorted if bias is introduced to the review process at any stage. For example, incomplete searching for all relevant studies may lead to publication bias; there is strong association between publication and studies reporting significant or positive findings in medicine11 and also within orthodontics.12 With regard to studies included within the review, these need to be assessed to ensure that they are undertaken and reported in a manner that is free of bias (systematic error).10 Outcome reporting bias (ORB) may also be relevant, e.g. in a study identified as relevant to a review, where data exist on the outcome of interest but only selected findings have been given based on the results.13 Although systematic reviews have emphasized ‘assessment of methodological quality’ of included studies, the Cochrane handbook recommends a focus on ‘risk of bias assessment’ instead.10 The justifications are four-fold: it targets whether the results of included studies should be credible; despite being carried out to the highest standards, a study may have risk of bias due to, e.g. impracticality/impossibility of blinding of participants/study personnel; some quality markers, e.g. a power calculation is unlikely to directly affect risk of bias;14 it overcomes ambiguity between quality of reporting and quality of the underlying research.10”...”There are many tools available; most are scales where quality components are scored and combined to give a summary score; or checklists of specific questions.15 As it is impossible to know the extent or true risk of bias in a given study, possible validation of any proposed tool is limited. The Cochrane-Collaboration recommends neither of the aforementioned methods; rather a domain-based evaluation (Yes/No/Unclear) is used where Yes indicates a ‘low risk of bias’ for the following six components: sequence generation, allocation concealment, blinding of participants/personnel and outcome assessors, incomplete outcome data, selective outcome reporting and other sources of bias.10,16 The issue of bias linked to poor randomization and blinding has been discussed extensively but the discussions about ORB are relatively new.16,17” |
| 139 | Moher | ”Strong evidence of outcome reporting bias was recently reported within clinical trials [23,24]. Our results suggest that some aspect of selective outcome reporting bias might also exist within non-Cochrane reviews. Only about one-quarter of them reported a primary outcome, of which half report statistical significance in favour of this outcome (versus 14.4% for Cochrane reviews). This issue requires further investigation.” |
| 140 | Morrison | ”We found no evidence of systematic bias from the use of language restrictions in SRs/MAs in conventional medicine. There were conflicting findings about the methodological and reporting quality of English-language versus languages other than English trials. These findings do not rule out the potential for language bias when language restrictions are used. Searches should include languages other than English studies when resources and time are available to minimize the risk of a biased summary effect. More research, in different medical specialties, will provide better evidence on the effect of language restriction on systematic reviews.” |
| 141 | Mullan | ”To help reduce reporting bias, and potentially allow for exploration about the impact of author contact on bias and the results of systematic reviews, journals and the Cochrane Collaboration should implement reporting recommendations for author contact in systematic reviews.”...”Author contact adds to the burden of conducting a systematic review. However, it can improve the quality of the review by: (1) confirming the accuracy of the extracted data; and (2) overcoming the data-driven reporting choices that authors and journals may have made. Lack of reporting or incomplete reporting of an important outcome de facto excludes an eligible study from analysis, a contributor to reporting bias if the outcome was in fact measured. Reporting bias can lead to overestimation of the treatment effect [3], and Chan and Altman have found that more than 20% of outcomes measured in parallel group trials were incompletely reported [2]. When are the benefits worth the costs and burden of contacting authors? Arguably, reviewers should contact authors when the review can yield stronger inferences as a result of this process. The extent to which reviewers can make this determination a priori remains uncertain and may represent an area of fruitful methodological investigation.”...”The inclusion of reporting recommendations for author contact in systematic reviews and the enforcement of these requirements by journals and the Cochrane Collaboration may allow for methodological explorations about the impact of author contact on bias and the results of systematic reviews. Essential elements to report include: the number of studies for which authors were contacted, the information requested, any important details of the method of eliciting information, and the response of authors to the request. When pertinent, authors should report the impact that information obtained from authors had on review results (i.e., using sensitivity analyses).” |
| 142 | Murad | ”Ideally, systematic reviewers will evaluate and report the risk of bias for each of the important outcomes measured in each individual study. There is no one correct way to assess the risk of bias.26 Review authors can use detailed checklists or focus on a few key aspects of the study. Different study designs require the use of different instruments (eg, for randomized clinical trials, the Cochrane Risk of Bias tool27). A judgment about the overall risk of bias for all of the included studies may then result in decreasing the confidence in estimates.5” |
| 143 | Murad  (2^nd^ peripheral remark by this author) | ”Although this systematic review has accounted for selection, performance, detection and attrition bias for all the identified studies, grave weakness is still there. As with many systematic reviews, the limited number of well-conducted RCT might reduce the effectiveness of this review in its ability to provide valuable scientific evidence to support or contribute to changes in our conventional clinical practice. Although efforts were made to avoid language bias and to examine the reference lists, no attempt was made to hand-search any journals and the only attempt to identify unpublished trials was made by contacting investigators of the included study.”...”The major weaknesses of this systematic review is that the result is derived from the inclusion of one sole industry sponsored trial, which failed to provide sufficient information about the randomisation procedure. This trial could therefore be considered to fall into the category of poor reporting. The assumption of inadequate randomisation method or bias interference generally indicates inadequate methods.” |
| 144 | Naci | ”While some aspects of clinician–industry interactions may be beneficial, the normalization of such relationships in clinical settings creates the potential for serious risks for patients and health care systems. Yet it may be unrealistic to expect that clinicians can be taught individually how to interact with industry ethically or to detect and avert bias. Social science researchers suggest that the rational choice view of conflict of interest does not reflect the evidence, arguing that judgments are subject to a “self-serving” bias that is both unconscious and unintentional [50]. The problem of self-serving bias suggests that clinician education will not be effective in mitigating unconscious biases, nor will disclosure be an effective means to counteract biases [50]. Further, even clinicians who consciously seek to avoid interactions with industry may fail because of the ubiquitous nature of marketing and promotional materials [51] and the strength of practice and social norms [26]. Although education alone may be ineffective, the ethical implications of such interactions could be problematized for clinicians during professional and continuing education, and issues such as the introduction of bias into clinical decision-making could be addressed at an institutional or regulatory level. ” |
| 145 | Nankervis | ”…only 5% of the recent eczema trials were registered correctly and with enough detail to assess outcome-reporting bias for the primary outcome.”...”The fact that some investigators chose to preregister their trial could be an indicator of trial quality, and this was explored using the four key domains known to be associated with a high risk of bias (Higgins and Altman, 2008). In this sample of eczema RCTs, there was a suggestion that trial quality might be improved in registered trials, but this was only significant for the domain of allocation concealment, and is possibly limited by the modest sample size of our survey.” |
| 146 | Palys | ”The authors are correct that the Jadad score, whether with 13, 11, or 5 points (as is most common), is efficient.1 That is to say that a score for trial quality can be whipped up with little effort. However, would it not be even more efficient to simply toss a coin and award full marks if that coin lands face up?”...” Flawed studies invite biases that distort findings, generally towards more favorable ones that suit the preferences of the experimenters and all of their conflicts of interest. When these studies go on to inform medical decisions, patients are denied the basic right of informed decision making because they are basing their decisions on information that is not only misleading, but also known to be misleading. The emperor's new clothes must finally be exposed for what they are, and the emperor's new clothing inspectors are part of the problem, and not part of the solution. We need new inspectors, ones who will actually inspect the clothing instead of simply tossing a coin (or, equivalently, using the Jadad score). Only then can we expect better clothes (or medical studies, as the case may be).” |
| 147 | Pearson | ”While the rigour of studies included in public health systematic reviews is routinely assessed using a quality appraisal tool,1–3 with the exception of the Cochrane risk of bias tool,1 none assesses potential bias to a subsequent review from missing or incompletely reported results. ”...”The aims of this paper were to demonstrate the use of a formal tool (Outcome Reporting Bias in Trials (ORBIT)10) to appraize studies included in a systematic review of public health interventions for the existence of outcome reporting bias and to assess the impact that this bias (where present) had on the evidence that was used to inform the development of guidance.”...”The aims of this paper were to demonstrate the use of a formal tool (Outcome Reporting Bias in Trials (ORBIT)10) to appraize studies included in a systematic review of public health interventions for the existence of outcome reporting bias and to assess the impact that this bias (where present) had on the evidence that was used to inform the development of guidance. ”...”Retrospective assessment of outcome reporting bias as part of critical appraisal tools used in public health18 19 is also possible but will require development of a tool such as ORBIT to be more suitable for the study designs and reporting found in evaluations of public health interventions.” |
| 148 | Pildal | ”Most conclusions favouring an intervention would lose support if trials with unclear or inadequate allocation concealment were excluded from the meta-analysis. This may seem too radical, especially since the bias associated with these trials appears to be smaller and less consistent than previously thought. Furthermore, the remaining trials might still be affected by other sources of bias, for instance selective reporting of significant outcomes.89 Yet, results of meta-analyses should always be accompanied by sensitivity analyses presenting the results with and without trials with unclear or inadequate bias protection. While sensitivity analyses will allow the reader to gauge the possible impact of bias, decisions still have to be made whether or not the investigated interventions should be implemented. To guide such decisions, further research on the size and direction of different types of bias under different circumstances is warranted. In addition, steps to prevent bias and avoid uncertainty regarding the level of bias protection should be taken. First, the gatekeepers of trial protocols (primarily drug-regulatory authorities and research ethics committees) should insist on description of methods to ensure allocation concealment and sanction only protocols with adequate methods. Secondly, trial protocols should be publicly available to facilitate critical appraisal of trials and thirdly, the CONSORT statement, 91 which requires explicit and appropriate reporting on measures taken to protect a trial against bias, should be broadly enforced.”...”Two thirds of conclusions drawn from meta-analyses loose support if only trials with reported adequate allocation concealment are relied upon. The impact of reported allocation concealment and double-blinding on the treatment effect estimate is smaller and less consistent than previously thought. It would be too radical to routinely only rely on trials with reported adequate allocation concealment.” |
| 149 | Poulsen | ”The experimental design of the trials also differed: nine of the 12 trials used the split-mouth design and the remaining three used a parallel-group design. The methodological problems associated with the split-mouth design have been reviewed recently, 1 and the possibility of a carry-over effect from one side of the mouth to the other has been pointed out as an important risk-of-bias. To what extent this factor has been taken into account in the trials is not reported.” |
| 150 | Purgato | ”25 scales that had been used to assess the validity or ‘quality’ of randomized trials. These scales included between 3 and 57 items and rating scales were likely to include criteria that did not directly relate to study validity (Cipriani et al., 2009). The Cochrane Collaboration recommends simple approaches for assessing methodological quality using a specific tool for evaluating the risk of bias. This tool – developed between 2005 and 2007 by a working group of methodologists – is a domain-based evaluation.”...”Theoretically, it is expected high coherence between the quality of reporting and the methodological quality. In practice, it is possible to see clinical trials with a high methodological quality that are poorly reported, but also high quality study reports of clinical trials with a poor methodological quality.”...”Even though to assess quality of RCTs is a complex issue, in order to have a rough idea of the quality of a RCT we recommend to have a careful look at the table reporting the baseline clinical and sociodemographic characteristics of the patients allocated to the new and reference treatment. It is possible to check how many patients were truly randomized (a small sample size generally correlates with low study quality) and whether there are imbalances in the randomization procedures that may be suggestive of high risk of bias.” |
| 151 | Rasines | ”For the assessment of risk of bias of the cross-sectional surveys with retrospective assessment of exposure, the authors developed their own criteria. None of the studies filled all the considered criteria.” |
| 152 | Reeves | ”Non-randomized studies vary in their design features (Reeves et al., 2008; Higgins et al., 2012). These features make the studies more or less susceptible to bias. In NRS, compared with in RCTs, attrition is often worse (and poorly reported); intervention and outcome assessments are less likely to be conducted according to standardized protocols, and outcomes are rarely assessed blind to the group allocation. Too often, these limitations of NRS are seen as part of doing an NRS with the consequence that their implications for risk of bias to a study (Higgins and Altman, 2008), and the way in which limitations vary across studies, are not properly considered. For example, some users of evidence may consider NRS that investigate long-term outcomes to have ‘better quality’ than randomized trials of short-term outcomes, simply on the basis of their relevance without appraising their risk of bias. Schünemann et al. (2013) describe how such a judgement can be made more systematically.”...”The Cochrane Collaboration has typically sought to summarize reliable evidence rather than the best available evidence (i.e. with an absolute threshold rather than a conditional rule for inclusion). Should it continue to do this? In what circumstances should NRS be included in Cochrane reviews? How acceptable is it for this decision to depend on (i) the anticipated needs of certain stakeholders; (ii) knowledge of how much randomized evidence exists; and (iii) the suspected magnitude of effect size? Should a common tool be used to assess the risk of bias in RCTs and non-randomized studies? What sort of tool would allow differentiation in risks of bias: (i) between RCTs and non-randomized studies, and (ii) between different types of NRS? How should review authors draw distinctions between different types of non-randomized evidence, particularly in relation to determining eligibility for the review? Are design labels (e.g. cohort study) or design features (e.g. prospective identification of participants) preferable? When should review authors be encouraged to use different study designs to assess unintended (e.g. adverse) effects compared with intended effects?” |
| 153 | Reveiz | “Overall, the proportion of adequate reporting varied by methodologic item from the Cochrane Collaboration Risk of Bias tool: random sequence generation (weighted proportion 5.7%, 95% CI 3.0–8.4%), allocation concealment (1.4%, 0–2.8%), blinding (41%, 35–47% including open label RCTs; 8.4%, 4.1–13% excluding open label RCTs), primary outcomes (66%, 60–72%), secondary outcomes (46%,40–52%) and harms outcomes (5%, 2–8%) (Table S1). Weighted proportions were calculated using data from Table 2. Most records reported no useful information for allocation concealment (97.9%) and harm (89.5%), and had insufficient detail for blinding (86.2%, excluding open label RCTs) and primary outcome measures (32%).” |
| 154 | Richards | ”While the authors reported the assessment of risk of as low for seven of the included studies in the discussion, they indicate that if they had included allocation concealment (a key component of bias protection) only one of the studies would be considered to have a low risk of bias. While I agree that this may be a reporting issue rather than a study conduct it does form an important element of assessing potential bias.” |
| 155 | Rubinstein | ”...only in the last period (2010 ) did more than half the studies have a low risk of bias, namely, 68%.”...”…performance bias is but one construct of methodological quality, and studies that do not “blind” their patients or practitioners may still be considered to have a low risk of bias even if other constructs are not entirely conducted well.”...”Blinding (or masking) of subjects in trials of nonpharmacological interventions remains a problem, and SMT is certainly no exception. It is questionable to what degree blinding is relevant. Although adequate blinding is absolutely necessary when comparing an intervention to a sham treatment, this is much less relevant for pragmatic studies where experimental interventions are typically compared with standard therapies, such as “usual care.” – “Therein also belies a problem because it assumes that all criteria weigh equally, and one could argue that randomization, for example, specifically treatment allocation, is perhaps the most important feature of trials.12” |
| 156 | Runnels | ”It was also clear from responses to the survey questions that respondents, a majority of whom had expertize in systematic review methodology, recognized the many limitations to the quality of evidence in primary studies, systematic reviews, and meta-analyses. These perspectives were evident in comments about poor quality protocols; the lack of transparency in clinical trial design; inadequate reporting of outcomes, including adverse events; in the challenges of conducting sub-group analyses; and in addressing the many forms of methods bias in appraisal of evidence. As Ioannidis and others have shown, seemingly rigorous methods have considerable strengths, “but can also lead to wrong or misleading answers” [54], p. 169]. For example, the outcomes of meta-analyses on the same topic may differ widely due to many factors, including that those conducting/sponsoring the meta-analyses may choose different data sources, search strategies, inclusion/exclusion criteria for eligible populations, and have different, sometimes conflicting interests that may influence how results are presented and interpreted [54].” |
| 157 | Saini | ”The classification system used in this study has been presented and applied during a workshop that we developed and delivered at international Cochrane colloquiums. The feedback from this workshop supported the practical application of our classification system, and many participants were able to relate their own experiences to the types of scenarios that are captured in the classification. Following the application of the classification system, the Cochrane risk of bias tool is currently being updated to include the assessment of bias in both randomized controlled trials and non-randomized studies. The proposed new structure of the risk of bias tool considers selective outcome reporting as being analogous to publication bias (non-reporting of whole studies). It is planned that this form of bias will be appraized outside the risk of bias tool…” |
| 158 | Santaguida | “When assessing limitations in studies of medical tests, systematic reviewers should select validated criteria that examine the risk of systematic error. Systematic reviewers should categorize the risk of bias for individual studies as “low,” “medium,” or “high.” Two reviewers should independently assess individual criteria as well as global categorization. Reviewers should establish methods for determining an overall categorization for the study limitations a priori and document these decisions clearly.” |
| 159 | Savović | ”Bias associated with specific reported study design characteristics of RCTs leads to exaggeration of intervention effect estimates and increases in between-trial heterogeneity. For each of the three characteristics assessed, these effects appeared greatest for subjectively assessed outcome measures. Assessments of the risk of bias in trial results should account for these findings. Downweighting trials at high risk of bias in future meta-analyses, based on these empirical findings, could be an alternative to completely excluding such trials from meta-analyses, resulting in a smaller loss of precision.” |
| 160 | Sequeira-Byron | “We suggest that the use of a tool such as AMSTAR in the pre-publication and editorial appraisal of systematic reviews would help to ensure that those that are published are of high quality and are reported clearly, completely and in such a way that this quality is readily apparent.” |
| 161 | Shadrick | ”Cochrane reviews are well known for their methodological rigour and minimisation of bias and this one certainly doesn't disappoint. It describes in comprehensive detail the methodology used and design of the review. The Cochrane Collaboration statistical guidelines were adhered to for data analysis with heterogeneity and bias also thoroughly assessed, although insufficient studies were identified to investigate reporting bias. Considerable effort was undertaken to contact the authors of the studies to confirm details missing from the methodology of the trials. The lack of long-term data and small sample sizes made it difficult for the authors to draw conclusions other than the need for more high quality long-term research into this area.” |
| 162 | Sidney | ”Risk of Bias: Unclear Items: Trends over time for studies scoring items as “unclear” are depicted in Figure 4. In more than half of all studies, 3 items (ie, treatment allocation, selective reporting, and cointerventions) were rated unclear. For many items, there appears to be a trend toward better reporting over time, which is most pronounced for items related to selection bias.”...”Nevertheless, performance bias is but one construct of methodological quality, and studies that do not “blind” their patients or practitioners may still be considered to have a low risk of bias even if other constructs are not entirely conducted well.” |
| 163 | Simon | ”Establish the blind is one thing, but maintaining throughout the follow-up is another. For this, we will sometimes use false adaptations doses. Such adaptations will be centralized. In this case, it is possible to generate false biological results. Using an assessor blinded endpoint is always useful (assessor distinguished therapist). If the assessor is not blind, the bias induced wherever endpoint is now well documented. [8] It will therefore avoid contact between Therapist and the non-blind evaluator. When necessary, the patient will be informed that he must not speak with the evaluator. In addition, it is helpful to use a centralized evaluation further investigations. In all cases, unblinding procedure should be described in the protocol, especially in emergency situations.”...”Entering a final question: should assess the success of the blind? In reality, this assessment of the blind be criticized. [9] Often evaluation focuses more on blind prejudices about the effectiveness of treatment on the blind itself. In addition, the repetition of these evaluations will effectively focus the participants on blind issues. Finally, being able to guess treatment (not certain) is not equivalent to the fact of knowing for sure: this methodological flaw probably not enough to bring the test at an open trial. This is why the 2010 version of Consort statement abandoned blind evaluation of methods [4] (although the latter was explicitly mentioned in recommendation 11 of the 2001 version). Nevertheless, it must accurately describe the method for the blind. Most of the time, these methods are insufficiently described, either in the article or in the protocol of [10] study. For our part, we do the same finding in preeclampsia prevention trials (personal data).” |
| 164 | Smaïl-Faugero | ”...Cochrane review authors describe clinical implications only after describing the quality of evidence and the balance of benefits and harms.” |
| 165 | Sola | ”Background: The Cochrane Handbook claims that the evaluation of the validity of included studies is essential for the analysis, interpretation and conclusions of systematic reviews. Therefore, reviews should include not only a risk of bias assessment but also should discuss how the limitations in the design and execution could affect the validity of their conclusions. Objectives: To evaluate to what extent the risk of bias assessment is properly considered to formulate the conclusions in set of published systematic reviews. Methods: Two reviewers will independently assess a set of systematic reviews used to develop a practice guideline on the surgical management of femoral fractures. We will use AMSTAR (Shea 2007), paying special attention to the relevant items that address whether the reviews documented any formal quality assessment (item 7), and if this assessment was used accurately to formulate the reviews' conclusions (item 8). We will estimate an agreement coefficient between these two items, but will also qualitatively check the extent to which the quality of information was used to draw conclusions in the reviews. Results: So far, we have assessed 15 systematic reviews (5 Cochrane vs. 10 non Cochrane). Although 9 of these reviews properly considered the limits in design and execution of the included trials to formulate their conclusions, the rest (6 reviews, 40%) did not assess the quality of the included studies or did not use the quality assessment to discuss its impact on the review results. At this moment we are increasing the sample of assessed reviews to accurately estimate if these preliminary results could be considered significant. Conclusions: There still exists clear room for improvement in the way that the quality assessment of included studies is used to draw conclusions in systematic reviews. This issue is of special relevance for the usefulness of reviews in the process of knowledge translation, given the importance of bias assessment for allowing an appropriate evaluation of the confidence in the estimates derived from reviews.” |
| 166 | Song | ”Exclusion of non-English language studies appears to result in a particularly high risk of bias in some areas of research such as complementary and alternative medicine.”...”Risk of bias was marked as ‘high’ if the efforts taken to minimize publication bias were partial or insufficient, publication bias was not discussed and the authors’ conclusions were positive. Risk of bias was ‘moderate’ if partial efforts were taken to minimize bias, publication bias was probably considered, and the author’s conclusions might have been positive with cautious interpretation. Risk of bias was ‘low’ if partial or sufficient efforts were taken to minimize bias.”...”Risk of bias was marked as ‘high’ if the efforts taken to minimize publication bias were partial or insufficient, publication bias was not discussed and the authors’ conclusions were positive. Risk of bias was ‘moderate’ if partial efforts were taken to minimize bias, publication bias was probably considered, and the author’s conclusions might have been positive with cautious interpretation. Risk of bias was ‘low’ if partial or sufficient efforts were taken to minimize bias.” |
| 167 | Souza | ”Asymmetrical in funnel plots are linked to publication bias although there are other sources of asymmetry that have to be considered, including other dissemination biases, differences in the quality of smaller studies, the existence of true heterogeneity, and chance. Asymmetry in funnel plots may be an indicator that a more detailed investigation should be carried out on the presence of heterogeneity, such as sensitivity analysis.” |
| 168 | Spuls | ”Systematic reviews should not be restricted to English-language publications, to prevent bias.” |
| 169 | Szczesniak | ”Studies of various quality levels can either mask or have an adverse effect. The quality of included studies, for example the study of Xu et al., is low and could have biased the results, as reported in the Cochrane Collaboration guidelines.2 The authors should have evaluated the methodology more accurately; they declare no publication bias in their funnel plot for mortality; at a closer inspection, however, a marked asymmetry in the lower left-hand corner is evident, suggesting markedly biased results.”...”…we believe the methodology employed in the article biased the results, which are against the current evidence of literature. Therefore, the findings of this meta-analysis should be interpreted with strong caution.” |
| 170 | Tendal | ”Meta-analyses of randomized clinical trials are crucial for making evidence based decisions. However, trial reports often present the same data in multiple forms when reporting different intervention groups, time points, and outcome measures.1 Although this multiplicity has always been a challenge in meta-analyses, its potential as a source of bias has received little attention.”...”Multiplicity of data in trial reports might lead to biased decisions about which data to include in meta-analyses and hence threaten the validity of their results. In this study, we empirically assessed whether selecting between multiple time points, scales, and treatment groups affected SMD results in a randomly selected sample of Cochrane reviews.”...”A typical statement, which allowed for a potentially biased choice regarding the selection of a time point, was: “All outcomes were reported for the short term (up to 12 weeks), medium term (13 to 26 weeks), and long term (more than 26 weeks).”7 ” |
| 171 | Thombs | ”Studies of the accuracy of depression screening tools rarely exclude already diagnosed or treated patients, a potential bias that is not evaluated in systematic reviews and meta-analyses. This may result in inflated accuracy estimates on which clinical practice and preventive care guidelines are often based, a problem that takes on greater importance as the rate of diagnosed and treated depression in the population increases.” |
| 172 | Thombs  (2^nd^ peripheral remark by this author) | “Studies of the accuracy of screening tools for depression rarely exclude patients who already have a diagnosis or are receiving treatment, a potential bias that is not evaluated in systematic reviews and meta-analyses. This can result in inflated accuracy and estimates of the yield of new cases on which clinical practice and preventive care guidelines are often based, a problem that takes on greater importance as the rate of diagnosed and treated depression in the population increases.” |
| 173 | Tricco | ”...the impact of this potential bias can be addressed through sensitivity analyses, which explores whether results of the review are robust to differences in the trials, such as methodology (eg, examining studies with and without allocation concealment analyzed separately) and populations examined.”...”Reviewers should consider both study- and review-level limitations. If the conduct or reporting of included studies is poor, the review conclusions may be biased and this should be stated explicitly. Furthermore, knowledge syntheses themselves can be susceptible to bias.”...”Although much attention has been paid to enhancing the quality of systematic reviews, relatively little attention has been paid to the format for presenting the review. Because the reporting of systematic reviews tends to focus on methodological rigor more than clinical context, they often do not provide crucial information for clinicians. In one study, the researchers found that of systematic reviews published in ACP JC and EBM Journal (journals of secondary publication), less than 15% of these had sufficient information about the intervention to allow clinicians or policy makers to implement it [78].” |
| 174 | Tricco  (2^nd^ peripheral remark by this author) | ”We identified SRs examining several biases, yet further investigation into the following is warranted: 1) place of publication bias, 2) country bias, 3) search bias, 4) citation bias, 5) multiple publication bias, and 6) outcome reporting bias (a SR is planned; Dr. P. Williamson, personal communication). The SRs themselves should be updated, as new evidence may have emerged. Although not a mandate of this review, we believe it is important to explore whether common SR practices do in fact decrease bias, such as having two people independently screen potentially relevant material and scanning the reference lists of the included studies in a SR.”...”Our findings recommend including unpublished material in SRs, updating SRs periodically, searching more than one database, hand searching for additional material, using the Cochrane HSSS to locate RCT reports, and assessing for publication bias. Further examination of the other types of bias identified in our SR is warranted and the existing empirical evidence should be systematically reviewed.” |
| 175 | Tugwell | ”When is the optimal time to introduce new health care technologies into routine care? Many clinical epidemiologists and approval agencies will suggest recommending their introduction only after consistent evidence of benefit is found from two pragmatic, well-designed, and well-executed randomized controlled trials (RCTs).”...”Integrating global research evidence with locally relevant evidence and contextual factors is rarely a transparent process.”...”Surrogate markers are very attractive as endpoints in trials – they have been popular since blood pressure was shown to predict morbidity and mortality, and a reduction in blood pressure was shown to reduce the death and cardiovascular morbidity. They are so much easier to measure, the trials so much easier to power, and hence so much cheaper. However, their indiscriminate use can cause enormous harm.”...”Response shift is the focus of an interesting study by Galenkamp et al. Why does self-rated health not decline as we get older? What could explain why ‘‘older olds'’ have been reported to rate their health more positively than ‘‘younger olds.’’ Given similar levels of limitations and chronic conditions, the authors examined 3 different components known to contribute to response shift (reprioritization, reconceptualization, and recalibration). They conclude that recalibration is probably the most responsible. To assess and to evaluate possible effects arising from web-based data collection on the results of a study, Mayr et al sought to assess and evaluate possible effects arising from selection bias and additional response bias. They did find a selection bias as their sample was found to be better educated, more often living in a partnership, more often female, and older than the general population. They also found a substantial response bias as participants using the internet were younger, better educated, and more often male compared with participants preferring the paper-and-pencil version. Despite this bias, the method of data collection had no direct effect on the results of various self-report instruments after adjusting for the 3 characteristics defining the response bias.” |
| 176 | Turner | ”Review methods: assessing risk of bias: The risk of bias assessment within included studies varied considerably across the samples. For example, although 28% (37/131) of CAM SRs and 17% (30/175) of control SRs used the Cochrane Risk of Bias tool [19], 83% of CAM reviews used a tool identified as relatively less frequently used [20] (e.g., MINORS [21], Downs and Black [22], Zaza [23]). Self-developed tools were used in 4% (5/131) of CAM reviews and 11% (19/175) of control SRs. Of the CAM reviews, 19/131 reviews used more than one tool (Table 3).”...”Risk of bias assessment within included studies varied considerably across the samples; 28% of CAM SRs and 17% of control SRs used the Cochrane Risk of Bias tool [19]. These findings are consistent with other research [27]. Moreover, 83% of CAM reviews used less prominent tools and self-developed tools were used in 4% of CAM reviews and 11% of control SRs. There are a substantial number of methods used to assess the quality of primary studies in both samples of SRs. This is consistent with previous research which reported of 177 reviews, 38% defined a method of quality assessment, within which 74 different methodological items and 26 different scales were identified [21].” |
| 177 | Turner  (2^nd^ peripheral remark by this author) | ”In our analyses, we have modelled total between-study heterogeneity, which is likely to comprize a mixture of variation caused by true diversity among the protocols for the original studies, variation caused by biases and unexplained variation. Assuming that a conventional random-effects model will be used in many future meta-analyses, it is appropriate to focus on total between-study heterogeneity in our predictive findings. However, it would be preferable to separate variation attributable to biases from other sources of between-study variation. In later versions of the CDSR, this will become possible once the recently introduced Cochrane risk-of-bias tool21 has been implemented in a large number of systematic reviews. Our existing hierarchical model for the data from all available meta-analyses could be extended to incorporate the bias model proposed by Welton et al.22 This would allow us to adjust for the bias attributable to a potential source (e.g. inadequate allocation concealment) in all studies judged to be at high risk. In principle, the model could be extended further to adjust for multiple sources of bias simultaneously. Results from this analysis could provide useful information about the degree to which one would expect between-study heterogeneity to reduce, on average, if meta-analysts chose to adjust for known sources of bias, for example, by using empirical evidence or elicited opinion on biases.22,23” |
| 178 | Turner  (3^rd^ peripheral remark by this author) | ”When at least two adequately powered studies are available in meta-analyses reported by Cochrane reviews, underpowered studies often contribute little information, and could be left out if a rapid review of the evidence is required. However, underpowered studies made up the entirety of the evidence in most Cochrane reviews.” |
| 179 | Van Driel | ”Our study shows that searching for unpublished trials in Cochrane reviews does not give a high yield and that the methodological quality of unpublished trials raizes concern. We found that only 11.9% of all recent Cochrane reviews included references to unpublished data. In an earlier literature review of 150 meta-analyses indexed in MEDLINE (1988–1999), 30.7% included unpublished data in their analysis [8]. Egger et al. report that only 7% of all trials included in their sample of 159 meta-analyses were unpublished [10] The attention on publication bias in the past decade and recommendations to search for unpublished material to minimize the risk of publication bias [9], [16] and [17] apparently have not resulted in more frequent inclusion of unpublished data in Cochrane reviews. Egger et al. found that funnel plot asymmetry, as a proxy of publication bias, was reduced when unpublished trials were included, but excluding them from the meta-analysis had only relatively small effects on the estimates of treatment and their precision [10]. However, the funnel plot may not be the most reliable method to detect publication bias [18].”...”Our review shows that less than 10% of Cochrane reviews include unpublished data and references to unpublished studies make up only a small proportion of all included studies. The fact that a third of these “unpublished” references could be located as journal publications suggests that not including them in the review before formal publication would merely delay the evidence synthesis. The poor methodological transparency and quality of the trials that remain unpublished is an important concern for the validity of the reviews. Cochrane reviewers should be aware of this issue and perhaps they could add to the transparency of their review by performing sensitivity analyses of trial quality. Some articles of high quality may remain unpublished because they show negative or indifferent results [40]. However, extensive searching has not uncovered these trial reports in Cochrane Reviews and reviewers need to weigh the importance of searching for (additional) unpublished studies in each individual review against the risk of introducing bias. It may be better to invest in improving (reporting of) methodological quality and regular updating of existing reviews that include good quality trials than in extensive searches for unpublished data.” |
| 180 | Viswanathan | ”While we were able to identify a set of questions for the most common observational study designs, we were not able to establish consensus on required items for each type of design, as we had originally intended. Whether our suggested approach applies to other quasi-experimental designs such as controlled clinical trials or pre-post studies of public health interventions also requires empirical assessment. More work is required to establish consensus on type of study designs and specific sources of bias for each design.” |
| 181 | Vollenweider | ”We believe that a way forward would be not only to inform investigators what ought to be carried out and reported (as is laid out in the CONSORT statement) but also to have a better explanation of how the features of trial design help reduce bias. A clinical trial is no different from any epidemiological study; the primary concern should be minimization of confounding, selection bias and measurement error that lead to information bias. There seems to be too little awareness of the problems of selection bias and missing data. A number of studies have found that many trials do not report or use inappropriate methods to conceal the random allocation [38]. Similarly, we found that many trials do not report on the handling of missing data and ways to deal with it. It is difficult to quantify the bias that results from different ways of (not) dealing with missing data because individual patient data from many different trials would be needed to investigate this. To improve reporting on missing data, editors and reviewers should require investigators to follow the CONSORT statement and report on their efforts to minimize bias from missing data and to report when they were unable to do so explicitly.” |
| 182 | Wong | ”the findings from this review should be interpreted with caution, because the methodologic assessment of the quality of the included studies showed an unclear risk of bias in five out of the ten included papers, and a high risk of bias in four studies.” |
| 183 | Yavchitz | ”However, despite these initiatives, the quality of reporting of abstracts remains questionable [9, 10, 22–24]. A recent study showed that despite systematic reviews including primary studies with high risk of bias, just over half included a risk of bias assessment in the interpretation of results in the abstract [13]. Consequently, adding a limitations section could be useful to enhance readers’ awareness and improve their interpretation. However, a limitations section in the abstract is recommended by only a few journals and for systematic reviews in the PRISMA statement for abstracts [12].” |

**References**

| 1. | Armijo-Olivo, Susan, Maria Ospina, Bruno R. da Costa, Matthias Egger, Humam Saltaji, Jorge Fuentes, Christine Ha, and Greta G. Cummings. “Poor Reliability between Cochrane Reviewers and Blinded External Reviewers When Applying the Cochrane Risk of Bias tool in Physical Therapy Trials.” PloS One 9, no. 5 (2014): e96920. doi:10.1371/journal.pone.0096920. |
| --- | --- |
| 2. | Bero, Lisa A. “Why the Cochrane Risk of Bias tool Should Include Funding Source as a Standard Item.” The Cochrane Database of Systematic Reviews 12 (2013): ED000075. |
| 3. | Boutron, Isabelle, and Philippe Ravaud. “Classification Systems to Improve Assessment of Risk of Bias.” Journal of Clinical Epidemiology 65, no. 3 (March 2012): 236–38. doi:10.1016/j.jclinepi.2011.09.006. |
| 4. | De Bruin, Marijn. “Risk of Bias in Randomized Controlled Trials of Health Behaviour Change Interventions: Evidence, Practices and Challenges.” Psychology & Health 30, no. 1 (January 2, 2015): 1–7. doi:10.1080/08870446.2014.960653. |
| 5. | Hartling, Lisa, Michele P. Hamm, Andrea Milne, Ben Vandermeer, P. Lina Santaguida, Mohammed Ansari, Alexander Tsertsvadze, Susanne Hempel, Paul Shekelle, and Donna M. Dryden. “Testing the Risk of Bias tool Showed Low Reliability between Individual Reviewers and across Consensus Assessments of Reviewer Pairs.” Journal of Clinical Epidemiology 66, no. 9 (September 2013): 973–81. doi:10.1016/j.jclinepi.2012.07.005. |
| 6. | Hróbjartsson, Asbjørn, Isabelle Boutron, Lucy Turner, Douglas G. Altman, and David Moher. “Assessing Risk of Bias in Randomized Clinical Trials Included in Cochrane Reviews: The Why Is Easy, the How Is a Challenge.” The Cochrane Database of Systematic Reviews 4 (2013): ED000058. doi:10.1002/14651858.ED000058. |
| 7. | Ivers, Noah M, Andrea C Tricco, Monica Taljaard, Ilana Halperin, Lucy Turner, David Moher, and Jeremy M Grimshaw. “Quality Improvement Needed in Quality Improvement Randomized Trials: Systematic Review of Interventions to Improve Care in Diabetes.” BMJ Open 3, no. 4 (April 9, 2013). doi:10.1136/bmjopen-2013-002727. |
| 8. | Jefferson, Tom, Mark A Jones, Peter Doshi, Chris B Del Mar, Rokuro Hama, Matthew J Thompson, Igho Onakpoya, and Carl J Heneghan. “Risk of Bias in Industry-Funded Oseltamivir Trials: Comparison of Core Reports versus Full Clinical Study Reports.” BMJ Open 4, no. 9 (September 30, 2014). doi:10.1136/bmjopen-2014-005253. |
| 9. | Katikireddi, Srinivasa Vittal, Matt Egan, and Mark Petticrew. “How Do Systematic Reviews Incorporate Risk of Bias Assessments into the Synthesis of Evidence? A Methodological Study.” Journal of Epidemiology and Community Health 69, no. 2 (February 1, 2015): 189–95. doi:10.1136/jech-2014-204711. |
| 10. | Morissette, Kate, Andrea C Tricco, Tanya Horsley, Maggie H Chen, and David Moher. “Blinded versus Unblinded Assessments of Risk of Bias in Studies Included in a Systematic Review.” In Cochrane Database of Systematic Reviews. John Wiley & Sons, Ltd, 1996. http://onlinelibrary.wiley.com/doi/10.1002/14651858.MR000025.pub2/abstract. |
| 11. | Moustgaard, Helene, Segun Bello, Franklin G. Miller, and Asbjørn Hróbjartsson. “Subjective and Objective Outcomes in Randomized Clinical Trials: Definitions Differed in Methods Publications and Were Often Absent from Trial Reports.” Journal of Clinical Epidemiology 67, no. 12 (December 2014): 1327–34. doi:10.1016/j.jclinepi.2014.06.020. |
| 12. | Roseman, M., E. H. Turner, J. Lexchin, J. C. Coyne, L. A. Bero, and B. D. Thombs. “Reporting of Conflicts of Interest from Drug Trials in Cochrane Reviews: Cross Sectional Study.” BMJ 345, no. aug16 3 (August 16, 2012): e5155–e5155. doi:10.1136/bmj.e5155. |
| 13. | Savović, Jelena, Laura Weeks, Jonathan A. C. Sterne, Lucy Turner, Douglas G. Altman, David Moher, and Julian P. T. Higgins. “Evaluation of the Cochrane Collaboration’s tool for Assessing the Risk of Bias in Randomized Trials: Focus Groups, Online Survey, Proposed Recommendations and Their Implementation.” Systematic Reviews 3 (2014): 37. doi:10.1186/2046-4053-3-37. |
| 14. | Sterne, Jonathan A. C. “Why the Cochrane Risk of Bias tool Should Not Include Funding Source as a Standard Item.” The Cochrane Database of Systematic Reviews 12 (2013): ED000076. |
| 15. | Vale, Claire L., Jayne F. Tierney, and Sarah Burdett. “Can Trial Quality Be Reliably Assessed from Published Reports of Cancer Trials: Evaluation of Risk of Bias Assessments in Systematic Reviews.” BMJ (Clinical Research Ed.) 346 (2013): f1798. |
| 16. | Abou-Setta A, Dryden D, Hamm M, Moher D, Klassen T, Hartling L. Assessment of trial risk of bias among Cochrane reviews: a cross-sectional analysis. Poster presentation at the 19th Cochrane Colloquium; 2011 Oct 19-22; Madrid, Spain. Cochrane Database of Systematic Reviews, Supplemen. |
| 17. | Akl, Elie A., Xin Sun, Jason W. Busse, Bradley C. Johnston, Matthias Briel, Sohail Mulla, John J. You, et al. “Specific Instructions for Estimating Unclearly Reported Blinding Status in Randomized Trials Were Reliable and Valid.” Journal of Clinical Epidemiology 65, no. 3 (March 2012): 262–67. doi:10.1016/j.jclinepi.2011.04.015. |
| 18. | Anette Blümle, Erik von Elm, Gerd Antes, Joerg J. Meerpohl. Measurement and assessment of study quality and reporting quality. Zeitschrift für Evidenz, Fortbildung und Qualität im Gesundheitswesen Volume 108, Issues 8–9, 2014, Pages 495–503 |
| 19. | Armijo-Olivo, Susan, Carla R. Stiles, Neil A. Hagen, Patricia D. Biondo, and Greta G. Cummings. “Assessment of Study Quality for Systematic Reviews: A Comparison of the Cochrane Collaboration Risk of Bias tool and the Effective Public Health Practice Project Quality Assessment tool: Methodological Research.” Journal of Evaluation in Clinical Practice 18, no. 1 (February 2012): 12–18. doi:10.1111/j.1365-2753.2010.01516.x. |
| 20. | Armijo-Olivo, Susan, Greta G. Cummings, Jorge Fuentes, Humam Saltaji, Christine Ha, Annabritt Chisholm, Dion Pasichnyk, and Todd Rogers. “Identifying Items to Assess Methodological Quality in Physical Therapy Trials: A Factor Analysis.” Physical Therapy 94, no. 9 (September 1, 2014): 1272–84. doi:10.2522/ptj.20130464. |
| 21. | Armijo-Olivo, Susan, Jorge Fuentes, Maria Ospina, Humam Saltaji, and Lisa Hartling. “Inconsistency in the Items Included in tools Used in General Health Research and Physical Therapy to Evaluate the Methodological Quality of Randomized Controlled Trials: A Descriptive Analysis.” BMC Medical Research Methodology 13 (September 17, 2013): 116. doi:10.1186/1471-2288-13-116. |
| 22. | Armijo-Olivo, Susan, Jorge Fuentes, Todd Rogers, Lisa Hartling, Humam Saltaji, and Greta G Cummings. “How Should We Evaluate the Risk of Bias of Physical Therapy Trials?: A Psychometric and Meta-Epidemiological Approach towards Developing Guidelines for the Design, Conduct, and Reporting of RCTs in Physical Therapy (PT) Area: A Study Protocol.” Systematic Reviews 2 (September 26, 2013): 88. doi:10.1186/2046-4053-2-88. |
| 23. | Armstrong, Rebecca, Elizabeth Waters, Maureen Dobbins, John N. Lavis, Mark Petticrew, and Robin Christensen. “Knowledge Translation Strategies for Facilitating Evidence-Informed Public Health Decision Making among Managers and Policy-Makers.” In Cochrane Database of Systematic Reviews. John Wiley & Sons, Ltd, 1996. http://onlinelibrary.wiley.com/doi/10.1002/14651858.CD009181/abstract. |
| 24. | Bafeta, Aïda, Agnes Dechartres, Ludovic Trinquart, Amélie Yavchitz, Isabelle Boutron, and Philippe Ravaud. “Impact of Single Centre Status on Estimates of Intervention Effects in Trials with Continuous Outcomes: Meta-Epidemiological Study.” BMJ : British Medical Journal 344 (2012). doi:10.1136/bmj.e813. |
| 25. | Barkhordarian, Andre, Peter Pellionisz, Mona Dousti, Vivian Lam, Lauren Gleason, Mahsa Dousti, Josemar Moura, and Francesco Chiappelli. “Assessment of Risk of Bias in Translational Science.” Journal of Translational Medicine 11 (August 8, 2013): 184. doi:10.1186/1479-5876-11-184. |
| 26. | Berkman, Nancy D., P. Lina Santaguida, Meera Viswanathan, and Sally C. Morton. “The Empirical Evidence of Bias in Trials Measuring Treatment Differences,” September 2014. http://www.ncbi.nlm.nih.gov/books/NBK253183/. |
| 27. | Bero, Lisa. “Bias Related to Funding Source in Statin Trials.” BMJ (Clinical Research Ed.) 349 (2014): g5949. |
| 28. | Buchberger, Dr Barbara, E. von Elm, G. Gartlehner, H. Huppertz, G. Antes, J. Wasem, and J. J. Meerpohl. “Bewertung des Risikos für Bias in kontrollierten Studien.” Bundesgesundheitsblatt - Gesundheitsforschung - Gesundheitsschutz 57, no. 12 (December 1, 2014): 1432–38. doi:10.1007/s00103-014-2065-6. |
| 29. | Cho, Hee Ju, Jae Hoon Chung, Jung Ki Jo, Dong Hyuk Kang, Jeong Man Cho, Tag Keun Yoo, and Seung Wook Lee. “Assessments of the Quality of Randomized Controlled Trials Published in International Journal of Urology from 1994 to 2011.” International Journal of Urology 20, no. 12 (2013): 1212–19. doi:10.1111/iju.12150. |
| 30. | Chung, Vincent CH, Robin ST Ho, Xinyin Wu, Daisy HY Fung, Xin Lai, Justin CW Wu, and Samuel YS Wong. “Are Meta-Analyses of Chinese Herbal Medicine Trials Trustworthy and Clinically Applicable? A Cross-Sectional Study.” Journal of Ethnopharmacology 162 (March 13, 2015): 47–54. doi:10.1016/j.jep.2014.12.028. |
| 31. | Crocetti, Michael T., Diane D. Amin, and Roberta Scherer. “Assessment of Risk of Bias Among Pediatric Randomized Controlled Trials.” Pediatrics 126, no. 2 (August 1, 2010): 298–305. doi:10.1542/peds.2009-3121. |
| 32. | Da Costa, Bruno R., Nina M. Resta, Brooke Beckett, Nicholas Israel-Stahre, Alison Diaz, Bradley C. Johnston, Matthias Egger, Peter Jüni, and Susan Armijo-Olivo. “Effect of Standardized Training on the Reliability of the Cochrane Risk of Bias Assessment tool: A Study Protocol.” Systematic Reviews 3 (2014): 144. doi:10.1186/2046-4053-3-144. |
| 33. | De Bruin, Marijn, Jim McCambridge, and Jan M. Prins. “Reducing the Risk of Bias in Health Behaviour Change Trials: Improving Trial Design, Reporting or Bias Assessment Criteria? A Review and Case Study.” Psychology & Health 30, no. 1 (January 2, 2015): 8–34. doi:10.1080/08870446.2014.953531. |
| 34. | Dechartres A, Altman DG, Trinquart L, Boutron I, and Ravaud P. “Association between Analytic Strategy and Estimates of Treatment Outcomes in Meta-Analyses.” JAMA 312, no. 6 (August 13, 2014): 623–30. doi:10.1001/jama.2014.8166. |
| 35. | Faggion, Clovis M., Fahd Huda, and Jason Wasiak. “Use of Methodological tools for Assessing the Quality of Studies in Periodontology and Implant Dentistry: A Systematic Review.” Journal of Clinical Periodontology 41, no. 6 (2014): 625–31. doi:10.1111/jcpe.12251. |
| 36. | Gordon H. Guyatt, Andrew D. Oxman, Gunn Vist, Regina Kunz, Jan Brozek, Pablo Alonso-Coello, Victor Montori, Elie A. Akl, Ben Djulbegovic, Yngve Falck-Ytter, Susan L. Norris, John W. Williams Jr., David Atkins, Joerg Meerpohl, Holger J. Schünemann, GRADE guidelines: 4. Rating the quality of evidence—study limitations (risk of bias), Journal of Clinical Epidemiology, Volume 64, Issue 4, April 2011, Pages 407-415, ISSN 0895-4356, http://dx.doi.org/10.1016/j.jclinepi.2010.07.017. |
| 37. | Hartling L, Bialy L, Armijo-Olivo S, Ha C, Lacaze-Masmonteil T, Vandermeer B, Dryden DM. Modifications to the Risk of Bias tool: a case study of 204 trials. Oral presentation at the 19th Cochrane Colloquium; 2011 Oct 19-22; Madrid, Spain. Cochrane Database of Systematic Reviews, Supplement. CMR-16534. |
| 38. | Hartling, L., Bond, K., Vandermeer, B., Seida, J., Dryden, D. M., & Rowe, B. H. (2011). Applying the Risk of Bias tool in a Systematic Review of Combination Long-Acting Beta-Agonists and Inhaled Corticosteroids for Persistent Asthma. PLoS ONE, 6(2), e17242. doi:10.1371/journal.pone.0017242 |
| 39. | Hartling, L., M. Hamm, T. Klassen, A.-W. Chan, M. Meremikwu, V. Moyer, S. Scott, D. Moher, M. Offringa, and for the StaR Child Health Group. “Standard 2: Containing Risk of Bias.” PEDIATRICS 129, no. Supplement (June 1, 2012): S124–31. doi:10.1542/peds.2012-0055E. |
| 40. | Hartling, Lisa, Maria Ospina, Yuanyuan Liang, Donna M Dryden, Nicola Hooton, Jennifer Krebs Seida, and Terry P Klassen. “Risk of Bias versus Quality Assessment of Randomized Controlled Trials: Cross Sectional Study.” BMJ : British Medical Journal 339 (2009). doi:10.1136/bmj.b4012. |
| 41. | Hayden, Jill A., Danielle A. van der Windt, Jennifer L. Cartwright, Pierre Côté, and Claire Bombardier. “Assessing Bias in Studies of Prognostic Factors.” Annals of Internal Medicine 158, no. 4 (February 19, 2013): 280–86. doi:10.7326/0003-4819-158-4-201302190-00009. |
| 42. | Hempel, Susanne, Jeremy NV Miles, Marika J Booth, Zhen Wang, Sally C Morton, and Paul G Shekelle. “Risk of Bias: A Simulation Study of Power to Detect Study-Level Moderator Effects in Meta-Analysis.” Systematic Reviews 2 (November 28, 2013): 107. doi:10.1186/2046-4053-2-107. |
| 43. | Higgins, Julian P T, Douglas G Altman, Peter C Gøtzsche, Peter Jüni, David Moher, Andrew D Oxman, Jelena Savović, Kenneth F Schulz, Laura Weeks, and Jonathan A C Sterne. “The Cochrane Collaboration’s tool for Assessing Risk of Bias in Randomized Trials.” BMJ : British Medical Journal 343 (2011). doi:10.1136/bmj.d5928. |
| 44. | Hopewell, Sally, Isabelle Boutron, Douglas G. Altman, and Philippe Ravaud. “Incorporation of Assessments of Risk of Bias of Primary Studies in Systematic Reviews of Randomized Trials: A Cross-Sectional Study.” BMJ Open 3, no. 8 (August 1, 2013): e003342. doi:10.1136/bmjopen-2013-003342. |
| 45. | Ijaz, Sharea, Jos H. Verbeek, Christina Mischke, and Jani Ruotsalainen. “Inclusion of Nonrandomized Studies in Cochrane Systematic Reviews Was Found to Be in Need of Improvement.” Journal of Clinical Epidemiology 67, no. 6 (June 2014): 645–53. doi:10.1016/j.jclinepi.2014.01.001. |
| 46. | Jo, Jung Ki, Jae Hoon Chung, Kyu Shik Kim, Jeong Woo Lee, Seung Wook Lee, and International Evidence-Based Medicine Research (IEMR) Group. “Randomized Controlled Trials in the Journal of Sexual Medicine: A Quality Assessment and Relevant Clinical Impact.” The Journal of Sexual Medicine 11, no. 4 (2014): 894–900. doi:10.1111/jsm.12455. |
| 47. | Johnson, Blair T., Robert E. Low, and Hayley V. MacDonald. “Panning for the Gold in Health Research: Incorporating Studies’ Methodological Quality in Meta-Analysis.” Psychology & Health 30, no. 1 (January 2, 2015): 135–52. doi:10.1080/08870446.2014.953533. |
| 48. | Kirkham, J. J, K. M Dwan, D. G Altman, C. Gamble, S. Dodd, R. Smyth, and P. R Williamson. “The Impact of Outcome Reporting Bias in Randomized Controlled Trials on a Cohort of Systematic Reviews.” BMJ 340, no. feb15 1 (February 15, 2010): c365–c365. doi:10.1136/bmj.c365. |
| 49. | Kirkham, Jamie J., Doug G. Altman, and Paula R. Williamson. “Bias Due to Changes in Specified Outcomes during the Systematic Review Process.” PLoS ONE 5, no. 3 (March 22, 2010): e9810. doi:10.1371/journal.pone.0009810. |
| 50. | Lee, Jeong Woo, Jae Hoon Chung, Jung Ki Jo, and Seung Wook Lee. “Analysis of Randomized Controlled Trials in Rheumatology International from 1981 to 2012: Methodological Assessment.” Rheumatology International 34, no. 9 (September 1, 2014): 1187–93. doi:10.1007/s00296-014-2963-9. |
| 51. | Liu, Yali, Shengping Yang, Junjie Dai, Yongteng Xu, Rui Zhang, Huaili Jiang, Xianxia Yan, and Kehu Yang. “Risk of Bias tool in Systematic Reviews/Meta-Analyses of Acupuncture in Chinese Journals.” PLoS ONE 6, no. 12 (December 9, 2011). doi:10.1371/journal.pone.0028130. |
| 52. | Lundh, Andreas, and Peter C Gøtzsche. “Recommendations by Cochrane Review Groups for Assessment of the Risk of Bias in Studies.” BMC Medical Research Methodology 8 (April 21, 2008): 22. doi:10.1186/1471-2288-8-22. |
| 53. | Lundh, Andreas, Sergio Sismondo, Joel Lexchin, Octavian A Busuioc, and Lisa Bero. “Industry Sponsorship and Research Outcome.” In Cochrane Database of Systematic Reviews. John Wiley & Sons, Ltd, 1996. http://onlinelibrary.wiley.com/doi/10.1002/14651858.MR000033.pub2/abstract. |
| 54. | Manchikanti, Laxmaiah, Joshua A. Hirsch, Steven P. Cohen, James E. Heavner, Frank J. E. Falco, Sudhir Diwan, Mark V. Boswell, et al. “Assessment of Methodologic Quality of Randomized Trials of Interventional Techniques: Development of an Interventional Pain Management Specific Instrument.” Pain Physician 17, no. 3 (June 2014): E263–90. |
| 55. | O’Connor, Annette M., and Jan M. Sargeant. “Critical Appraisal of Studies Using Laboratory Animal Models.” ILAR Journal 55, no. 3 (January 1, 2014): 405–17. doi:10.1093/ilar/ilu038. |
| 56. | Oremus, Mark, Carolina Oremus, Geoffrey B. C. Hall, and Margaret C. McKinnon. “Inter-Rater and Test–retest Reliability of Quality Assessments by Novice Student Raters Using the Jadad and Newcastle–Ottawa Scales.” BMJ Open 2, no. 4 (January 1, 2012): e001368. doi:10.1136/bmjopen-2012-001368. |
| 57. | Robertson C, Ramsay C, Gurung T, Mowatt G, Pickard R, Sharma P. Assessing the quality of non-randomized comparative studies: our experience of using the Cochrane Collaboration's risk of bias tool. Poster presentation at the 19th Cochrane Colloquium; 2011 Oct 19-22; Madrid, Spain. Cochrane Database of Systematic Reviews, Supplement. |
| 58. | Robinson, Karen A, Evelyn P Whitlock, Maya E Oneil, Johanna K Anderson, Lisa Hartling, Donna M Dryden, Mary Butler, et al. “Integration of Existing Systematic Reviews into New Reviews: Identification of Guidance Needs.” Systematic Reviews 3 (June 23, 2014): 60. doi:10.1186/2046-4053-3-60. |
| 59. | Roseman M, Milette K, Bero LA, and et al. “REporting of Conflicts of Interest in Meta-Analyses of Trials of Pharmacological Treatments.” JAMA 305, no. 10 (March 9, 2011): 1008–17. doi:10.1001/jama.2011.257. |
| 60. | Sargeant, J. M., and A. M. O’Connor. “Conducting Systematic Reviews of Intervention Questions II: Relevance Screening, Data Extraction, Assessing Risk of Bias, Presenting the Results and Interpreting the Findings.” Zoonoses and Public Health 61 (2014): 39–51. doi:10.1111/zph.12124. |
| 61. | Savovic J, Altman D, Higgins J, Moher D, Sterne J, Turner L, Weeks L. Risk of bias tool evaluation: process and results. Oral presentation at the Joint Cochrane and Campbell Colloquium; 2010 Oct 18-22; Keystone, Colorado, USA. Database of Systematic Reviews, Supplement |
| 62. | Shamliyan, Tatyana, Robert L. Kane, and Stacy Dickinson. “A Systematic Review of tools Used to Assess the Quality of Observational Studies That Examine Incidence or Prevalence and Risk Factors for Dizeases.” Journal of Clinical Epidemiology 63, no. 10 (October 2010): 1061–70. doi:10.1016/j.jclinepi.2010.04.014. |
| 63. | Shrier I. http://methods.cochrane.org/sites/methods.cochrane.org/files/Why%20Obs%20and%20DAG%20and%20Meta-analysis.pdf |
| 64. | Sinha, Yashwant K., Jonathan C. Craig, Premala Sureshkumar, Andrew Hayen, and Jo-anne E. Brien. “Risk of Bias in Randomized Trials of Pharmacological Interventions in Children and Adults.” The Journal of Pediatrics 165, no. 2 (August 2014): 367–71.e1. doi:10.1016/j.jpeds.2014.03.058. |
| 65. | Turner, Lucy, Isabelle Boutron, Asbjørn Hróbjartsson, Douglas G Altman, and David Moher. “The Evolution of Assessing Bias in Cochrane Systematic Reviews of Interventions: Celebrating Methodological Contributions of the Cochrane Collaboration.” Systematic Reviews 2 (September 23, 2013): 79. doi:10.1186/2046-4053-2-79. |
| 66. | Voss, Peer H., and Eva A. Rehfuess. “Quality Appraisal in Systematic Reviews of Public Health Interventions: An Empirical Study on the Impact of Choice of tool on Meta-Analysis.” Journal of Epidemiology and Community Health 67, no. 1 (January 1, 2013): 98–104. doi:10.1136/jech-2011-200940. |
| 67. | Wells, George A, Beverley Shea, Julian PT Higgins, Jonathan Sterne, Peter Tugwell, and Barnaby C Reeves. “Checklists of Methodological Issues for Review Authors to Consider When Including Non-Randomized Studies in Systematic Reviews.” Research Synthesis Methods 4, no. 1 (2013): 63–77. doi:10.1002/jrsm.1077. |
| 68. | Zeng, Xiantao, Yonggang Zhang, Joey S.W. Kwong, Chao Zhang, Sheng Li, Feng Sun, Yu-Ming Niu, and Liang Du. “The Methodological Quality Assessment tools for Pre-Clinical and Clinical Studies, Systematic Review and Meta-Analysis, and Clinical Practice Guideline: A Systematic Review.” Journal of Evidence-Based Medicine, 2015, n/a – n/a. doi:10.1111/jebm.12141. |
| 69. | Bala, Malgorzata M., Elie A. Akl, Xin Sun, Dirk Bassler, Dominik Mertz, Filip Mejza, Per Olav Vandvik, et al. “Randomized Trials Published in Higher vs. Lower Impact Journals Differ in Design, Conduct, and Analysis.” Journal of Clinical Epidemiology 66, no. 3 (March 2013): 286–95. doi:10.1016/j.jclinepi.2012.10.005. |
| 70. | Balk, Ethan M., Mei Chung, Nira Hadar, Kamal Patel, Winifred W. Yu, Thomas A. Trikalinos, and Lina Kong Win Chang. Accuracy of Data Extraction of Non-English Language Trials with Google Translate. AHRQ Methods for Effective Health Care. Rockville (MD): Agency for Healthcare Research and Quality (US), 2012. http://www.ncbi.nlm.nih.gov/books/NBK95238/. |
| 71. | Bassler, Dirk, Victor M. Montori, Matthias Briel, Paul Glasziou, Stephen D. Walter, Tim Ramsay, and Gordon Guyatt. “Reflections on Meta-Analyses Involving Trials Stopped Early for Benefit: Is There a Problem and If So, What Is It?” Statistical Methods in Medical Research 22, no. 2 (April 1, 2013): 159–68. doi:10.1177/0962280211432211. |
| 72. | Berger, Vance W. “Internal Validity and the Risk of Bias: A Case for a Comprehensive Review.” Journal of Anesthesia 26, no. 5 (June 1, 2012): 802–3. doi:10.1007/s00540-012-1420-8. |
| 73. | Berger, Vance W., and Anh-Chi Do. “Allocation Concealment Continues to Be Misunderstood.” Journal of Clinical Epidemiology 63, no. 4 (April 1, 2010): 468–69. doi:10.1016/j.jclinepi.2009.09.004. |
| 74. | Bialy, Liza, Ben Vandermeer, Thierry Lacaze-Masmonteil, Donna M. Dryden, and Lisa Hartling. “A Meta-Epidemiological Study to Examine the Association between Bias and Treatment Effects in Neonatal Trials.” Evidence-Based Child Health: A Cochrane Review Journal 9, no. 4 (2014): 1052–59. doi:10.1002/ebch.1985. |
| 75. | Chaimani et al. Effects of study precision and risk of bias in networks of interventions: a network meta-epidemiological study. meta-epidemiological study 2013 |
| 76. | Chase, Jonathan M. “The Shadow of Bias.” PLoS Biology 11, no. 7 (July 2013). doi:10.1371/journal.pbio.1001608. |
| 77. | Chess, Laura E, and Joel Gagnier. “Risk of Bias of Randomized Controlled Trials Published in Orthopaedic Journals.” BMC Medical Research Methodology 13 (June 9, 2013): 76. doi:10.1186/1471-2288-13-76. |
| 78. | Chiappelli, Francesco, André Barkhordarian C Phil, Rashi Arora, Linda Phi, Amy Giroux, Molly Uyeda, Jason Kung, and Manisha Ramchandani. “Reliability of Quality Assessments in Research Synthesis: Securing the Highest Quality Bioinformation for HIT.” Bioinformation 8, no. 14 (July 21, 2012): 691–94. doi:10.6026/97320630008691. |
| 79. | Christensen R. Is Phytalgic® a goldmine for osteoarthritis patients or is there something fishy about this nutraceutical? A summary of findings and risk-of-bias assessment. Arthritis Research & Therapy 2010, 12:105 |
| 80. | Chung, Jae Hoon, Dong Hyuk Kang, Jung Ki Jo, and Seung Wook Lee. “Assessing the Quality of Randomized Controlled Trials Published in the Journal of Korean Medical Science from 1986 to 2011.” Journal of Korean Medical Science 27, no. 9 (September 2012): 973–80. doi:10.3346/jkms.2012.27.9.973. |
| 81. | Chung, Mei, Ethan M Balk, Stanley Ip, Gowri Raman, Winifred W Yu, Thomas A Trikalinos, Alice H Lichtenstein, Elizabeth A Yetley, and Joseph Lau. “Reporting of Systematic Reviews of Micronutrients and Health: A Critical appraisal1234.” The American Journal of Clinical Nutrition 89, no. 4 (April 2009): 1099–1113. doi:10.3945/ajcn.2008.26821. |
| 82. | Da Costa, Bruno R., Roger Hilfiker, and Matthias Egger. “PEDro’s Bias: Summary Quality Scores Should Not Be Used in Meta-Analysis.” Journal of Clinical Epidemiology 66, no. 1 (January 1, 2013): 75–77. doi:10.1016/j.jclinepi.2012.08.003. |
| 83. | Davey, Jonathan, Rebecca M Turner, Mike J Clarke, and Julian PT Higgins. “Characteristics of Meta-Analyses and Their Component Studies in the Cochrane Database of Systematic Reviews: A Cross-Sectional, Descriptive Analysis.” BMC Medical Research Methodology 11 (November 24, 2011): 160. doi:10.1186/1471-2288-11-160. |
| 84. | Dechartres, Agnes, Isabelle Boutron, Ludovic Trinquart, Pierre Charles, and Philippe Ravaud. “Single-Center Trials Show Larger Treatment Effects Than Multicenter Trials: Evidence From a Meta-Epidemiologic Study.” Annals of Internal Medicine 155, no. 1 (July 5, 2011): 39–51. doi:10.7326/0003-4819-155-1-201107050-00006. |
| 85. | Doi, Suhail A. R., and Jan J. Barendregt. “Not PEDro’s Bias: Summary Quality Scores Can Be Used in Meta-Analysis.” Journal of Clinical Epidemiology 66, no. 8 (August 2013): 940–41. doi:10.1016/j.jclinepi.2013.03.001. |
| 86. | Dossing, Anna, Simon Tarp, Daniel E Furst, Christian Gluud, Joseph Beyene, Bjarke B Hansen, Henning Bliddal, and Robin Christensen. “Interpreting Trial Results Following Use of Different Intention-to-Treat Approaches for Preventing Attrition Bias: A Meta-Epidemiological Study Protocol.” BMJ Open 4, no. 9 (September 25, 2014). doi:10.1136/bmjopen-2014-005297. |
| 87. | Duclos, P., D. N. Durrheim, A. L. Reingold, Z. A. Bhutta, K. Vannice, and H. Rees. “Developing Evidence-Based Immunization Recommendations and GRADE.” Vaccine 31, no. 1 (December 17, 2012): 12–19. doi:10.1016/j.vaccine.2012.02.041. |
| 88. | Duvendack, Maren, Jorge Garcia Hombrados, Richard Palmer-Jones, and Hugh Waddington. “Assessing ‘what Works’ in International Development: Meta-Analysis for Sophisticated Dummies.” Journal of Development Effectiveness 4, no. 3 (September 1, 2012): 456–71. doi:10.1080/19439342.2012.710642. |
| 89. | Dwan, Kerry, Douglas G. Altman, Juan A. Arnaiz, Jill Bloom, An-Wen Chan, Eugenia Cronin, Evelyne Decullier, et al. “Systematic Review of the Empirical Evidence of Study Publication Bias and Outcome Reporting Bias.” PLoS ONE 3, no. 8 (August 28, 2008). doi:10.1371/journal.pone.0003081. |
| 90. | Dwan, Kerry, Douglas G. Altman, Mike Clarke, Carrol Gamble, Julian P. T. Higgins, Jonathan A. C. Sterne, Paula R. Williamson, and Jamie J. Kirkham. “Evidence for the Selective Reporting of Analyses and Discrepancies in Clinical Trials: A Systematic Review of Cohort Studies of Clinical Trials.” PLoS Medicine 11, no. 6 (June 24, 2014). doi:10.1371/journal.pmed.1001666. |
| 91. | Elkhadem A. Question: How effective is fluoride toothpaste in preventing dental caries in the primary dentition of preschool children? Dos Santos AP, Nadanovsky P, de Oliveira BH. A systematic review and meta-analysis of the effects of fluoride toothpastes on the prevention of dental caries in the primary dentition of preschool children. Community Dent Oral Epidemiol 2012; doi: 10.1111/j.1600-0528.2012.00708.x. [Epub ahead of print] PubMed PMID: 22882502. |
| 92. | ElKhadem A. Question: When bonding orthodontic appliances are 1-step bonding approaches more effective than 2-step approaches? Fleming PS, Johal A, Pandis N. Self-etch primers and conventional acid-etch technique for orthodontic bonding: a systematic review and meta-analysis. Am J Orthod Dentofacial Orthop. 2012; 142: 83–94. |
| 93. | Engebretson S. Question: Does non-surgical periodontal treatment improve glycaemic control in diabetic patients? Corbella S, Francetti L, Taschieri S, De Siena F, Fabbro MD. Effect of periodontal treatment on glycemic control of patients with diabetes: A systematic review and meta-analysis. J Diabetes Investig 2013; 4: 502–509. |
| 94. | Eriksen, P., Bartels, E. M., Altman, R. D., Bliddal, H., Juhl, C. and Christensen, R. (2014), Risk of Bias and Brand Explain the Observed Inconsistency in Trials on Glucosamine for Symptomatic Relief of Osteoarthritis: A Meta-Analysis of Placebo-Controlled Trials. Arthritis Care Res, 66: 1844–1855. doi: 10.1002/acr.22376 |
| 95. | Faggion, Clovis Mariano, Nikolaos Nikitas Giannakopoulos, and Stefan Listl. “Risk of Bias of Animal Studies on Regenerative Procedures for Periodontal and Peri-Implant Bone Defects – a Systematic Review.” Journal of Clinical Periodontology 38, no. 12 (2011): 1154–60. doi:10.1111/j.1600-051X.2011.01783.x. |
| 96. | Christiane Alves FerreiraI; Carlos Alfredo Salles LoureiroII; Humberto SaconatoIII; Álvaro Nagib AtallahIV. Assessing the risk of bias in randomized controlled trials in the field of dentistry indexed in the Lilacs (Literatura Latino-Americana e do Caribe em Ciências da Saúde) database. Sao Paulo Med. J. vol.129 no.2 São Paulo Mar. 2011 |
| 97. | Fleming, Padhraig S., Jadbinder Seehra, Argy Polychronopoulou, Zbys Fedorowicz, and Nikolaos Pandis. “Cochrane and Non-Cochrane Systematic Reviews in Leading Orthodontic Journals: A Quality Paradigm?” The European Journal of Orthodontics, April 16, 2012, cjs016. doi:10.1093/ejo/cjs016. |
| 98. | Flodgren G, Parmelli E, Doumit G, Gattellari M, O’Brien MA, Grimshaw J, Eccles MP. Local opinion leaders: effects on professional practice and health care outcomes. Cochrane Database of Systematic Reviews 2011, Issue 8. Art. No.: CD000125. DOI: 10.1002/14651858.CD000125.pub4. |
| 99. | Flores-Mir C. Question: How effective are orthodontic appliances in distalising upper first molars in children and adolescents? Jambi S, Thiruvenkatachari B, O'Brien KD, Walsh T. Orthodontic treatment for distalising upper first molars in children and adolescents. Cochrane Database Syst Rev 2013; 10: Art. No.: CD008375. DOI: 10.1002/14651858.CD008375.pub2. |
| 100. | Forbes, Dorothy. “Blinding: An Essential Component in Decreasing Risk of Bias in Experimental Designs.” Evidence Based Nursing 16, no. 3 (July 1, 2013): 70–71. doi:10.1136/eb-2013-101382. |
| 101. | Foster, Nadine, and Paul Little. “Methodological Issues in Pragmatic Trials of Complex Interventions in Primary Care.” The British Journal of General Practice 62, no. 594 (January 2012): 10–11. doi:10.3399/bjgp12X616238. |
| 102. | Freemantle, N., and F. V. d. Werf. “Keeping Patients Safe While Avoiding Bias in Randomized Trials.” BMJ 344, no. may14 1 (May 14, 2012): e3297–e3297. doi:10.1136/bmj.e3297. |
| 103. | Frosi, Giacomo, Richard D. Riley, Paula R. Williamson, and Jamie J. Kirkham. “Multivariate Meta-Analysis Helps Examine the Impact of Outcome Reporting Bias in Cochrane Rheumatoid Arthritis Reviews.” Journal of Clinical Epidemiology. Accessed March 1, 2015. doi:10.1016/j.jclinepi.2014.11.017. |
| 104. | Garcia R. Question: Does the treatment of periodontitis and gingivitis during pregnancy reduce the number of preterm births? Polyzos NP, Polyzos IP, Zavos A, Valachis A, Mauri D, Papanikolaou EG, Tzioras S, Weber D, Messinis IE. Obstetric outcomes after treatment of periodontal dizease during pregnancy: systematic review and meta-analysis. BMJ 2010; 341: c701 |
| 105. | Ghaeminia H. Question: In patients with impacted third molars does coronectomy when compared to conventional removal result in fewer complications? Long H, Zhou Y, Liao L, Pyakurel U, Wang Y, Lai W. Coronectomy vs. total removal for third molar extraction: a systematic review. J Dent Res 2012; 91: 659–665. |
| 106. | Goodman, Steven, and Kay Dickersin. “Metabias: A Challenge for Comparative Effectiveness Research.” Annals of Internal Medicine 155, no. 1 (July 5, 2011): 61–62. doi:10.7326/0003-4819-155-1-201107050-00010. |
| 107. | Greenhalgh, T., J. Howick, N. Maskrey, and for the Evidence Based Medicine Renaissance Group. “Evidence Based Medicine: A Movement in Crisis?” BMJ 348, no. jun13 4 (June 13, 2014): g3725–g3725. doi:10.1136/bmj.g3725. |
| 108. | Hamm, Michele P, Shannon D Scott, Terry P Klassen, David Moher, and Lisa Hartling. “Do Health Care Institutions Value Research? A Mixed Methods Study of Barriers and Facilitators to Methodological Rigor in Pediatric Randomized Trials.” BMC Medical Research Methodology 12 (October 18, 2012): 158. doi:10.1186/1471-2288-12-158. |
| 109. | Hamm, Michele P., Terry P. Klassen, Shannon D. Scott, David Moher, and Lisa Hartling. “Education in Health Research Methodology: Use of a Wiki for Knowledge Translation.” PLoS ONE 8, no. 5 (May 31, 2013). doi:10.1371/journal.pone.0064922. |
| 110. | Hansen, Julie B., Carsten B. Juhl, Isabelle Boutron, Peter Tugwell, Elizabeth A. T. Ghogomu, Jordi Pardo Pardo, Tamara Rader, et al. “Assessing Bias in Osteoarthritis Trials Included in Cochrane Reviews: Protocol for a Meta-Epidemiological Study.” BMJ Open 4, no. 10 (2014): e005491. doi:10.1136/bmjopen-2014-005491. |
| 111. | Hartling, Lisa, Kenneth Bond, Krystal Harvey, P. Lina Santaguida, Meera Viswanathan, and Donna M. Dryden. Developing and Testing a tool for the Classification of Study Designs in Systematic Reviews of Interventions and Exposures. AHRQ Methods for Effective Health Care. Rockville (MD): Agency for Healthcare Research and Quality (US), 2010. http://www.ncbi.nlm.nih.gov/books/NBK52670/. |
| 112. | Hartling, Lisa, Michele P. Hamm, Ricardo M. Fernandes, Donna M. Dryden, and Ben Vandermeer. “Quantifying Bias in Randomized Controlled Trials in Child Health: A Meta-Epidemiological Study.” PLoS ONE 9, no. 2 (February 4, 2014). doi:10.1371/journal.pone.0088008. |
| 113. | He, Jia, Liang Du, Guanjian Liu, Jin Fu, Xiangyu He, Jiayun Yu, and Lili Shang. “Quality Assessment of Reporting of Randomization, Allocation Concealment, and Blinding in Traditional Chinese Medicine RCTs: A Review of 3159 RCTs Identified from 260 Systematic Reviews.” Trials 12, no. 1 (May 13, 2011): 122. doi:10.1186/1745-6215-12-122. |
| 114. | Herrera. Question: Does photodynamic therapy (PDT) improve periodontal outcomes compared to scaling and root planing (SRP) or no treatment? Azarpazhooh A, Shah PS, Tenenbaum HC, Goldberg MB. The effect of photodynamic therapy for periodontitis: A Systematic Review and Meta-Analysis. J Periodontol 2010; 81: 4–14. |
| 115. | Hopp, Lisa. “Risk of Bias Reporting in Cochrane Systematic Reviews.” International Journal of Nursing Practice, 2014, n/a – n/a. doi:10.1111/ijn.12252. |
| 116. | Hróbjartsson, A, and I Boutron. “Blinding in Randomized Clinical Trials: Imposed Impartiality.” Clinical Pharmacology & Therapeutics 90, no. 5 (November 2011): 732–36. doi:10.1038/clpt.2011.207. |
| 117. | Innes N. Question: Are individuals born with isolated oral clefts more likely to have dental anomalies than unaffected individuals? Tannure PN, Oliveira CA, Maia LC, Vieira AR, Granjeiro JM, De Castro Costa M. Prevalence of dental anomalies in nonsyndromic individuals with cleft lip and palate: A systematic review and meta-analysis. Cleft Palate Craniofac J 2012; 49: 194–200. |
| 118. | Jakobsen, Janus Christian, Jørn Wetterslev, Per Winkel, Theis Lange, and Christian Gluud. “Thresholds for Statistical and Clinical Significance in Systematic Reviews with Meta-Analytic Methods.” BMC Medical Research Methodology 14, no. 1 (November 21, 2014). doi:10.1186/1471-2288-14-120. |
| 119. | Jo, Jung Ki, Jae Hoon Chung, Kyu Shik Kim, Soo Hyun Song, and Seung Wook Lee. “Reporting of Randomized Controlled Trials in Andrology Journals: A Quality Assessment.” The Journal of Sexual Medicine, 2015, n/a – n/a. doi:10.1111/jsm.12784. |
| 120. | Johnston, Bradley C, Donald L Patrick, Jason W Busse, Holger J Schünemann, Arnav Agarwal, and Gordon H Guyatt. “Patient-Reported Outcomes in Meta-Analyses – Part 1: Assessing Risk of Bias and Combining Outcomes.” Health and Quality of Life Outcomes 11 (July 1, 2013): 109. doi:10.1186/1477-7525-11-109. |
| 121. | Jørgensen, Anders W, Katja L Maric, Britta Tendal, Annesofie Faurschou, and Peter C Gøtzsche. “Industry-Supported Meta-Analyses Compared with Meta-Analyses with Non-Profit or No Support: Differences in Methodological Quality and Conclusions.” BMC Medical Research Methodology 8 (September 9, 2008): 60. doi:10.1186/1471-2288-8-60. |
| 122. | Kalha AS. Question: What is the best orthodontic treatment approach for prominent upper front teeth? Thiruvenkatachari B, Harrison JE, Worthington HV, O'Brien KD. Orthodontic treatment for prominent upper front teeth (Class II malocclusion) in children. Cochrane Database Syst Rev 2013; 11: Art. No.: CD003452. DOI: 10.1002/14651858.CD003452.pub3. |
| 123. | Kirkham, Jamie J., Richard D. Riley, and Paula R. Williamson. “A Multivariate Meta-Analysis Approach for Reducing the Impact of Outcome Reporting Bias in Systematic Reviews.” Statistics in Medicine 31, no. 20 (2012): 2179–95. doi:10.1002/sim.5356. |
| 124. | Koretz, Ronald L., and Timothy O. Lipman. “The Presence and Effect of Bias in Trials of Early Enteral Nutrition in Critical Care.” Clinical Nutrition 33, no. 2 (April 2014): 240–45. doi:10.1016/j.clnu.2013.06.006. |
| 125. | Kraglund F. Question: Are triclosan/copolymer containing fluoride toothpastes more effective than fluoride toothpastes for control of caries, plaque and gingivitis in children and adults? Riley P, Lamont T. Triclosan/copolymer containing toothpastes for oral health. Cochrane Database Syst Rev 2013; 12: Art. No.: CD010514. DOI: 10.1002/14651858.CD010514.pub2. |
| 126. | Krauth, David, Tracey J. Woodruff, and Lisa Bero. “Instruments for Assessing Risk of Bias and Other Methodological Criteria of Published Animal Studies: A Systematic Review.” Environmental Health Perspectives 121, no. 9 (September 2013): 985–92. doi:10.1289/ehp.1206389. |
| 127. | Krauth, David, Tracey J. Woodruff, and Lisa Bero. “Instruments for Assessing Risk of Bias and Other Methodological Criteria: Krauth et Al. Respond.” Environmental Health Perspectives 122, no. 3 (March 2014): A67. doi:10.1289/ehp.1307727R. |
| 128. | Lambert. Question: What is the effectiveness of fluoride supplements (tablets, drops, lozenges and chewing gums) for preventing dental caries in children? Tubert-Jeannin S, Auclair C, Amsallem E, et al. Fluoride supplements (tablets, drops, lozenges or chewing gums) for preventing dental caries in children. Cochrane Database Syst Rev 2011; 12: CD007592 |
| 129. | Langford, Rebecca, Christopher P Bonell, Hayley E Jones, Theodora Pouliou, Simon M Murphy, Elizabeth Waters, Kelli A Komro, Lisa F Gibbs, Daniel Magnus, and Rona Campbell. “The WHO Health Promoting School Framework for Improving the Health and Well-Being of Students and Their Academic Achievement.” In Cochrane Database of Systematic Reviews. John Wiley & Sons, Ltd, 1996. http://onlinelibrary.wiley.com/doi/10.1002/14651858.CD008958.pub2/abstract. |
| 130. | Li, Tianjing, Milo A Puhan, Swaroop S Vedula, Sonal Singh, and Kay Dickersin. “Network Meta-Analysis-Highly Attractive but More Methodological Research Is Needed.” BMC Medicine 9 (June 27, 2011): 79. doi:10.1186/1741-7015-9-79. |
| 131. | Lim, Sun Mi, Ein Soon Shin, Sun Hee Lee, Kyung Hwa Seo, Yu Min Jung, and Ji Eun Jang. “tools for assessing quality and risk of bias by levels of evidence.” Journal of the Korean Medical Association 54, no. 4 (2011): 419. doi:10.5124/jkma.2011.54.4.419. |
| 132. | Louis, Deepak, Kiran More, Sapna Oberoi, and Prakesh S. Shah. “Intravenous Immunoglobulin in Isoimmune Haemolytic Dizease of Newborn: An Updated Systematic Review and Meta-Analysis.” Archives of Dizease in Childhood - Fetal and Neonatal Edition 99, no. 4 (July 1, 2014): F325–31. doi:10.1136/archdischild-2013-304878. |
| 133. | Lutomski JE, Donders AT, and Melis RF. “CAusal Diagrams to Better Understand Missingness.” JAMA Pediatrics 168, no. 2 (February 1, 2014): 187–187. doi:10.1001/jamapediatrics.2013.3650. |
| 134. | MA Jie,LIU Ying,ZHONG Lai-ping,ZHANG Chen-ping,ZHANG Zhi-yuan. Comparison between Jadad scale and Cochrane collaboration's tool for assessing risk of bias on the quality and risk of bias evaluation in randomized controlled trials (Department of Oral & Maxillofacial-Head & Neck Oncology,Ninth People's Hospital,College of Stomatology,Shanghai Jiao Tong University School of Medicine;Shanghai Key Laboratory of Stomatology.Shanghai 200011,China, China Journal of Oral and Maxillofacial Surgery 2012-05 |
| 135. | MacLennan S, Imamura M, Dahm P, Neuberger M, Reeves B, MacLennan G, Omar MI, McClinton S, Griffiths L, N'Dow J. Assessing risk of bias in non-randomized studies and incorporating GRADE: initial experience with a new Cochrane 'Risk of bias' tool under development. Poster presentation at the 19th Cochrane Colloquium; 2011 Oct 19-22; Madrid, Spain. Cochrane Database of Systematic Reviews, Supplement. |
| 136. | Matthews D. Weak evidence to support benefit of periodontal maintenance therapy in prevention of tooth loss. Chambrone L, Chambrone D, Lima LA, Chambrone LA. Predictors of tooth loss during long-term periodontal maintenance: a systematic review of observational studies. J Clin Periodontol. 2010; 37: 675–684. |
| 137. | McCay, L. “Ban against Industry Ties Introduces Bias and Obscures Whole View.” BMJ 343, no. sep06 4 (September 6, 2011): d5602–d5602. doi:10.1136/bmj.d5602. |
| 138. | Millett, Declan. “Bias in Systematic Reviews?” Journal of Orthodontics 38, no. 3 (September 1, 2011): 158–60. doi:10.1179/14653121141407. |
| 139. | Moher, David, Jennifer Tetzlaff, Andrea C Tricco, Margaret Sampson, and Douglas G Altman. “Epidemiology and Reporting Characteristics of Systematic Reviews.” PLoS Medicine 4, no. 3 (March 2007). doi:10.1371/journal.pmed.0040078. |
| 140. | Morrison, Andra, Julie Polizena, Don Husereau, Kristen Moulton, Michelle Clark, Michelle Fiander, Monika Mierzwinski-Urban, Tammy Clifford, Brian Hutton, and Danielle Rabb. “THE EFFECT OF ENGLISH-LANGUAGE RESTRICTION ON SYSTEMATIC REVIEW-BASED META-ANALYSES: A SYSTEMATIC REVIEW OF EMPIRICAL STUDIES.” International Journal of Technology Assessment in Health Care 28, no. 02 (April 2012): 138–44. doi:10.1017/S0266462312000086. |
| 141. | Mullan, Rebecca J., David N. Flynn, Bo Carlberg, Imad M. Tleyjeh, Celia C. Kamath, Matthew L. LaBella, Patricia J. Erwin, Gordon H. Guyatt, and Victor M. Montori. “Systematic Reviewers Commonly Contact Study Authors but Do so with Limited Rigor.” Journal of Clinical Epidemiology 62, no. 2 (February 2009): 138–42. doi:10.1016/j.jclinepi.2008.08.002. |
| 142. | Murad M, Montori VM, Ioannidis JA, and et al. “How to Read a Systematic Review and Meta-Analysis and Apply the Results to Patient Care: Users’ Guides to the Medical Literature.” JAMA 312, no. 2 (July 9, 2014): 171–79. doi:10.1001/jama.2014.5559. |
| 143. | Murad M. No available evidence to assess the effectiveness of bonded amalgams. Fedorowicz Z, Nasser M, Wilson N. Adhesively bonded versus non-bonded amalgam restorations for dental caries. Cochrane Database Syst Rev 2009; issue 4. |
| 144. | Naci, Huseyin, Sofia Dias, and A E Ades. “Industry Sponsorship Bias in Research Findings: A Network Meta-Analysis of LDL Cholesterol Reduction in Randomized Trials of Statins.” BMJ : British Medical Journal 349 (October 3, 2014). doi:10.1136/bmj.g5741. |
| 145. | Nankervis, Helen, Akerke Baibergenova, Hywel C. Williams, and Kim S. Thomas. “Prospective Registration and Outcome-Reporting Bias in Randomized Controlled Trials of Eczema Treatments: A Systematic Review.” Journal of Investigative Dermatology 132, no. 12 (December 2012): 2727–34. doi:10.1038/jid.2012.231. |
| 146. | Palys, Kaitlin E., and Vance W. Berger. “A Note on the Jadad Score as an Efficient tool for Measuring Trial Quality.” Journal of Gastrointestinal Surgery 17, no. 6 (December 12, 2012): 1170–71. doi:10.1007/s11605-012-2106-0. |
| 147. | Pearson, Mark, and Jaime Peters. “Outcome Reporting Bias in Evaluations of Public Health Interventions: Evidence of Impact and the Potential Role of a Study Register.” Journal of Epidemiology and Community Health 66, no. 4 (April 1, 2012): 286–89. doi:10.1136/jech.2010.122465. |
| 148. | Pildal, J., A. Hróbjartsson, K. J. Jørgensen, J. Hilden, D. G. Altman, and P. C. Gøtzsche. “Impact of Allocation Concealment on Conclusions Drawn from Meta-Analyses of Randomized Trials.” International Journal of Epidemiology 36, no. 4 (August 1, 2007): 847–57. doi:10.1093/ije/dym087. |
| 149. | Poulsen S. Available evidence does not support use of oxalates for dentine hypersensitivity. Cunha-Cruz J, Stout JR, Heaton LJ, Wataha JC; Northwest PRECEDENT Dentin hypersensitivity and oxalates: a Systematic Review. J Dent Res 2011; 90: 304–310. Epub 2010 Dec 29. |
| 150. | Purgato, Marianna. “Assessing Risk of Bias in Randomized Controlled Trials.” Epidemiologia E Psichiatria Sociale 19, no. 4 (2010): 296–97. |
| 151. | Rasines G. Using a fluoridated supplement with a high fluoride concentration in children aged under 6 years may increase the risk of fluorosis. Wong MCM, Glenny AM, Tsang BWK, Lo ECM, Worthington HV, Marinho VCC. Topical fluoride as a cause of dental fluorosis in children. Cochrane Database Syst Rev 2010; issue 1 |
| 152. | Reeves, Barnaby C., Julian P. T. Higgins, Craig Ramsay, Beverley Shea, Peter Tugwell, and George A. Wells. “An Introduction to Methodological Issues When Including Non-Randomized Studies in Systematic Reviews on the Effects of Interventions.” Research Synthesis Methods 4, no. 1 (2013): 1–11. doi:10.1002/jrsm.1068. |
| 153. | Reveiz, Ludovic, An-Wen Chan, Karmela Krleža-Jerić, Carlos Eduardo Granados, Mariona Pinart, Itziar Etxeandia, Diego Rada, Monserrat Martinez, Xavier Bonfill, and Andrés Felipe Cardona. “Reporting of Methodologic Information on Trial Registries for Quality Assessment: A Study of Trial Records Retrieved from the WHO Search Portal.” PLoS ONE 5, no. 8 (August 31, 2010): e12484. doi:10.1371/journal.pone.0012484. |
| 154. | Richards D. Limited evidence suggests that mouthrinses may be effective in reducing oral malodour |
| 155. | Rubinstein, Sidney M., Rik van Eekelen, Teddy Oosterhuis, Michiel R. de Boer, Raymond W. J. G. Ostelo, and Maurits W. van Tulder. “The Risk of Bias and Sample Size of Trials of Spinal Manipulative Therapy for Low Back and Neck Pain: Analysis and Recommendations.” Journal of Manipulative and Physiological Therapeutics 37, no. 8 (October 2014): 523–41. doi:10.1016/j.jmpt.2014.07.007. |
| 156. | Runnels, Vivien, Sari Tudiver, Marion Doull, and Madeline Boscoe. “The Challenges of Including Sex/gender Analysis in Systematic Reviews: A Qualitative Survey.” Systematic Reviews 3 (April 10, 2014): 33. doi:10.1186/2046-4053-3-33. |
| 157. | Saini, Pooja, Yoon K Loke, Carrol Gamble, Douglas G Altman, Paula R Williamson, and Jamie J Kirkham. “Selective Reporting Bias of Harm Outcomes within Studies: Findings from a Cohort of Systematic Reviews.” BMJ : British Medical Journal 349 (November 21, 2014). doi:10.1136/bmj.g6501. |
| 158. | Santaguida, P. Lina, Crystal M. Riley, and David B. Matchar. “Chapter 5: Assessing Risk of Bias as a Domain of Quality in Medical Test Studies.” Journal of General Internal Medicine 27, no. 1 (May 31, 2012): 33–38. doi:10.1007/s11606-012-2030-8. |
| 159. | Savović, J., He Jones, Dg Altman, Rj Harris, P. Jűni, J. Pildal, B. Als-Nielsen, et al. “Influence of Reported Study Design Characteristics on Intervention Effect Estimates from Randomized Controlled Trials: Combined Analysis of Meta-Epidemiological Studies.” Health Technology Assessment (Winchester, England) 16, no. 35 (September 2012): 1–82. doi:10.3310/hta16350. |
| 160. | Sequeira-Byron, Patrick, Zbys Fedorowicz, Vanitha A. Jagannath, and Mohammad Owaize Sharif. “An AMSTAR Assessment of the Methodological Quality of Systematic Reviews of Oral Healthcare Interventions Published in the Journal of Applied Oral Science (JAOS).” Journal of Applied Oral Science 19, no. 5 (October 2011): 440–47. doi:10.1590/S1678-77572011000500002. |
| 161. | Shadrick V. Facemask therapy between ages six to ten years may lead to short term improvements for Class III malocclusions. Watkinson S, Harrison JE, Furness S, Worthington HV. Orthodontic treatment for prominent lower front teeth (Class III malocclusion) in children. Cochrane Database Syst Rev 2013; 9: Art. No. CD003451. DOI: 10.1002/14651858.CD003451.pub2. |
| 162. | Sidney et al. The Risk of Bias and Sample Size of Trials of Spinal Manipulative Therapy for Low Back and Neck Pain: Analysis and Recommendations. Journal of Manipulative and Physiological Therapeutics. Volume 37, Issue 8, October 2014, Pages 523–541 |
| 163. | Simon, E. -G., C. -J. Arthuis, and F. Perrotin. “L’aveugle Dans Les Essais Contrôlés Randomisés.” Gynécologie Obstétrique & Fertilité 41, no. 2 (February 2013): 144–46. doi:10.1016/j.gyobfe.2012.12.005. |
| 164. | Smaïl-Faugeron, Violaine, Hélène Fron-Chabouis, and Frédéric Courson. “Methodological Quality and Implications for Practice of Systematic Cochrane Reviews in Pediatric Oral Health: A Critical Assessment.” BMC Oral Health 14 (April 9, 2014): 35. doi:10.1186/1472-6831-14-35. |
| 165. | Sola I, Martinez L, Roque M, Bonfill X. Do systematic reviews really accurately use the risk of bias assessment to draw conclusions? Poster presentation at the Joint Cochrane and Campbell Colloquium; 2010 Oct 18-22; Keystone, Colorado, USA. |
| 166. | Song, F, S Parekh, L Hooper, Yk Loke, J Ryder, Aj Sutton, C Hing, Cs Kwok, C Pang, and I Harvey. “Dissemination and Publication of Research Findings: An Updated Review of Related Biases.” Health Technol Assess 14, no. 8 (February 2010). doi:10.3310/hta14080. |
| 167. | Souza, João P, Cynthia Pileggi, and José G Cecatti. “Assessment of Funnel Plot Asymmetry and Publication Bias in Reproductive Health Meta-Analyses: An Analytic Survey.” Reproductive Health 4 (April 16, 2007): 3. doi:10.1186/1742-4755-4-3. |
| 168. | Spuls, Phyllis I., and Alexander Nast. “Evaluation of and Perspectives on Guidelines: What Is Important?” Journal of Investigative Dermatology 130, no. 10 (October 2010): 2348–49. doi:10.1038/jid.2010.247. |
| 169. | Szczesniak, Anna, Bartosz Gorny, Mariusz Kowalewski, Michalina Kolodziejczak, and Natalia Kotlarek. “Things to Avoid in Meta-Analysis.” European Journal of Preventive Cardiology 20, no. 3 (June 1, 2013): 513–513. doi:10.1177/2047487313484123. |
| 170. | Tendal, Britta, Eveline Nüesch, Julian P T Higgins, Peter Jüni, and Peter C Gøtzsche. “Multiplicity of Data in Trial Reports and the Reliability of Meta-Analyses: Empirical Study.” BMJ : British Medical Journal 343 (2011). doi:10.1136/bmj.d4829. |
| 171. | Thombs B, Arthurs E, El-Baalbaki G, Meijer A, Ziegelstein R, Steele R. Forecasting yesterday's weather: the risk of spectrum bias from the inclusion of already diagnosed/treated patients in studies of depression screening tools. Oral presentation at the 19th Cochrane Colloquium; 2011 Oct 19-22; Madrid, Spain [abstract]. Cochrane Database of Systematic Reviews, Supplement |
| 172. | Thombs, B. D., E. Arthurs, G. El-Baalbaki, A. Meijer, R. C. Ziegelstein, and R. J. Steele. “Risk of Bias from Inclusion of Patients Who Already Have Diagnosis of or Are Undergoing Treatment for Depression in Diagnostic Accuracy Studies of Screening tools for Depression: Systematic Review.” BMJ 343, no. aug18 1 (August 18, 2011): d4825–d4825. doi:10.1136/bmj.d4825. |
| 173. | Tricco, Andrea C., Jennifer Tetzlaff, and David Moher. “The Art and Science of Knowledge Synthesis.” Journal of Clinical Epidemiology 64, no. 1 (January 2011): 11–20. doi:10.1016/j.jclinepi.2009.11.007. |
| 174. | Tricco, Andrea C., Jennifer Tetzlaff, Margaret Sampson, Dean Fergusson, Elize Cogo, Tanya Horsley, and David Moher. “Few Systematic Reviews Exist Documenting the Extent of Bias: A Systematic Review.” Journal of Clinical Epidemiology 61, no. 5 (May 2008): 422–34. doi:10.1016/j.jclinepi.2007.10.017. |
| 175. | Tugwell, Peter, André Knottnerus, and Leanne Idzerda. “How Many Risks-of-Bias and Comorbidity Scales Do We Need?” Journal of Clinical Epidemiology 65, no. 9 (September 2012): 919–20. doi:10.1016/j.jclinepi.2012.07.001. |
| 176. | Turner, Lucy, James Galipeau, Chantelle Garritty, Eric Manheimer, L. Susan Wieland, Fatemeh Yazdi, and David Moher. “An Evaluation of Epidemiological and Reporting Characteristics of Complementary and Alternative Medicine (CAM) Systematic Reviews (SRs).” PLoS ONE 8, no. 1 (January 14, 2013). doi:10.1371/journal.pone.0053536. |
| 177. | Turner, Rebecca M, Jonathan Davey, Mike J Clarke, Simon G Thompson, and Julian PT Higgins. “Predicting the Extent of Heterogeneity in Meta-Analysis, Using Empirical Data from the Cochrane Database of Systematic Reviews.” International Journal of Epidemiology 41, no. 3 (June 2012): 818–27. doi:10.1093/ije/dys041. |
| 178. | Turner, Rebecca M., Sheila M. Bird, and Julian P. T. Higgins. “The Impact of Study Size on Meta-Analyses: Examination of Underpowered Studies in Cochrane Reviews.” PLoS ONE 8, no. 3 (March 27, 2013). doi:10.1371/journal.pone.0059202. |
| 179. | Van Driel, Mieke L., An De Sutter, Jan De Maeseneer, and Thierry Christiaens. “Searching for Unpublished Trials in Cochrane Reviews May Not Be Worth the Effort.” Journal of Clinical Epidemiology 62, no. 8 (August 2009): 838–44.e3. doi:10.1016/j.jclinepi.2008.09.010. |
| 180. | Viswanathan, Meera, Nancy D. Berkman, Donna M. Dryden, and Lisa Hartling. Assessing Risk of Bias and Confounding in Observational Studies of Interventions or Exposures: Further Development of the RTI Item Bank. AHRQ Methods for Effective Health Care. Rockville (MD): Agency for Healthcare Research and Quality (US), 2013. http://www.ncbi.nlm.nih.gov/books/NBK154461/. |
| 181. | Vollenweider, Daniela, Cynthia M Boyd, and Milo A Puhan. “High Prevalence of Potential Biases Threatens the Interpretation of Trials in Patients with Chronic Dizease.” BMC Medicine 9 (June 13, 2011): 73. doi:10.1186/1741-7015-9-73. |
| 182. | Wong. Systemic nucleoside antiviral agents may be effective in prevention of recurrent herpes labialis. Rahimi H, Mara T, Costella J, Speechley M, Bohay R. Effectiveness of antiviral agents for the prevention of recurrent herpes labialis: a systematic review and meta-analysis. Oral Surg Oral Med Oral Pathol Oral Radiol. 2012; 113: 618–627. |
| 183. | Yavchitz, Amélie, Philippe Ravaud, Sally Hopewell, Gabriel Baron, and Isabelle Boutron. “Impact of Adding a Limitations Section to Abstracts of Systematic Reviews on Readers’ Interpretation: A Randomized Controlled Trial.” BMC Medical Research Methodology 14, no. 1 (November 24, 2014). doi:10.1186/1471-2288-14-123. |
